# Analyzing molecular typing and clinical application of immunogenic cell death-related genes in hepatocellular carcinoma

**DOI:** 10.1186/s12885-023-10992-2

**Published:** 2023-06-08

**Authors:** Cai-Feng Lin, Zhi-Wen Chen, Feng-Ping Kang, Jian-Fei Hu, Long Huang, Cheng-Yu Liao, Jian-Lin Lai, Yi Huang, Zu-Wei Wang, Yi-Feng Tian, Shi Chen

**Affiliations:** 1 Shengli Clinical Medical College of Fujian Medical University, Fuzhou, 350001 Fujian Province People’s Republic of China; 2 Department of Hepatopancreatobiliary Surgery, Fujian Provincial Hospital, Fujian Medical University, No. 134, East Street, Fuzhou, 350001 Fujian Province People’s Republic of China; 3 Center for Experimental Research in Clinical Medicine, Fujian Provincial Hospital, Fuzhou, 350001 Fujian Province People’s Republic of China; 4 Department of Hepatobiliary Surgery, Shengli Clinical Medical College of Fujian Medical University, No. 134, East Street, Fuzhou, 350001 Fujian Province PR China; 5 Shengli Clinical Medical College of Fujian Medical University, Fujian Medical University, Fuzhou, 350001 China

**Keywords:** Hepatocellular carcinoma_1_, Immunogenic cell death_2_, Tumor microenvironment_3_, Prognostic model_4_, Immunotherapy_5_

## Abstract

**Background:**

Hepatocellular carcinoma (HCC) is considered one of the most common cancers, characterized by low early detection and high mortality rates, and is a global health challenge. Immunogenic cell death (ICD) is defined as a specific type of regulated cell death (RCD) capable of reshaping the tumor immune microenvironment by releasing danger signals that trigger immune responses, which would contribute to immunotherapy.

**Methods:**

The ICD gene sets were collected from the literature. We collected expression data and clinical information from public databases for the HCC samples in our study. Data processing and mapping were performed using R software to analyze the differences in biological characteristics between different subgroups. The expression of the ICD representative gene in clinical specimens was assessed by immunohistochemistry, and the role of the representative gene in HCC was evaluated by various in vitro assays, including qRT-PCR, colony formation, and CCK8 assay. Lasso-Cox regression was used to screen prognosis-related genes, and an ICD-related risk model (ICDRM) was constructed. To improve the clinical value of ICDRM, Nomograms and calibration curves were created to predict survival probabilities. Finally, the critical gene of ICDRM was further investigated through pan-cancer analysis and single-cell analysis.

**Results:**

We identified two ICD clusters that differed significantly in terms of survival, biological function, and immune infiltration. As well as assessing the immune microenvironment of tumors in HCC patients, we demonstrate that ICDRM can differentiate ICD clusters and predict the prognosis and effectiveness of therapy. High-risk subpopulations are characterized by high TMB, suppressed immunity, and poor survival and response to immunotherapy, whereas the opposite is true for low-risk subpopulations.

**Conclusions:**

This study reveals the potential impact of ICDRM on the tumor microenvironment (TME), immune infiltration, and prognosis of HCC patients, but also a potential tool for predicting prognosis.

**Supplementary Information:**

The online version contains supplementary material available at 10.1186/s12885-023-10992-2.

## Background

Hepatocellular carcinoma (HCC), the sixth most common type of cancer and the fourth leading cause of cancer-related death, is the most common primary liver cancer [1]. More than 700,000 new cases of liver cancer are diagnosed worldwide each year, half of which are in China [2]. Several treatments are generally available for early diagnosis, including surgical resection, radiofrequency ablation, transarterial chemoembolization, or liver transplantation. However, a diagnosis of HCC at an advanced stage usually renders many treatment strategies ineffective, and immunotherapy has demonstrated powerful antitumor effects in these patients [3]. Cancer immunotherapy uses the immune system to induce an antitumor immune response. Stress-induced regulatory cell death can trigger an inflammatory response that may culminate in the activation of adaptive immunity driven by cytotoxic T lymphocytes (CTL) and the establishment of long-term immune memory [4].

In vitro, the treatment of cancer cell lines with anthracyclines, oxaliplatin, photodynamic therapy, or gamma irradiation followed by subcutaneous transplantation into homozygous immunoreactive mice acted as cancer vaccines without any adjuvant or immunostimulatory substance [5]. The underlying mechanism is that these exogenous injuries activate tumor cells through signaling molecules that produce damage-associated molecular patterns (DAMPs), leading to the development of ICD. Key components include calreticulin, which is exposed on the cell surface. High Mobility Histone 1 (HMGB1) is secreted by tumor cells, and ATP molecules are released from the cells and heat shock proteins. These danger signals activate the innate immune system, which further enhances adaptive immunity [6], highlighting the critical role of the immune system in cancer treatment, and TME is considered to play a crucial role in HCC development [7].

This study first investigated ICD-associated genes(ICDs) expression in the TCGA-HCC cohort. Two clusters of ICDs were defined by an unsupervised clustering algorithm and analyzed for differences in functional annotation and immune infiltration between the two clusters. We then used the ICDs to construct ICDRM for predicting survival and immunotherapy response. The stability and reliability of ICDRM were further validated on different platform datasets. Exploring ICDs’ characteristics may help develop more precise cancer treatment strategies. Finally, further investigated the critical gene of ICDRM.

## Methods

### Acquisition of gene sets and study population

**Fig. 1:**
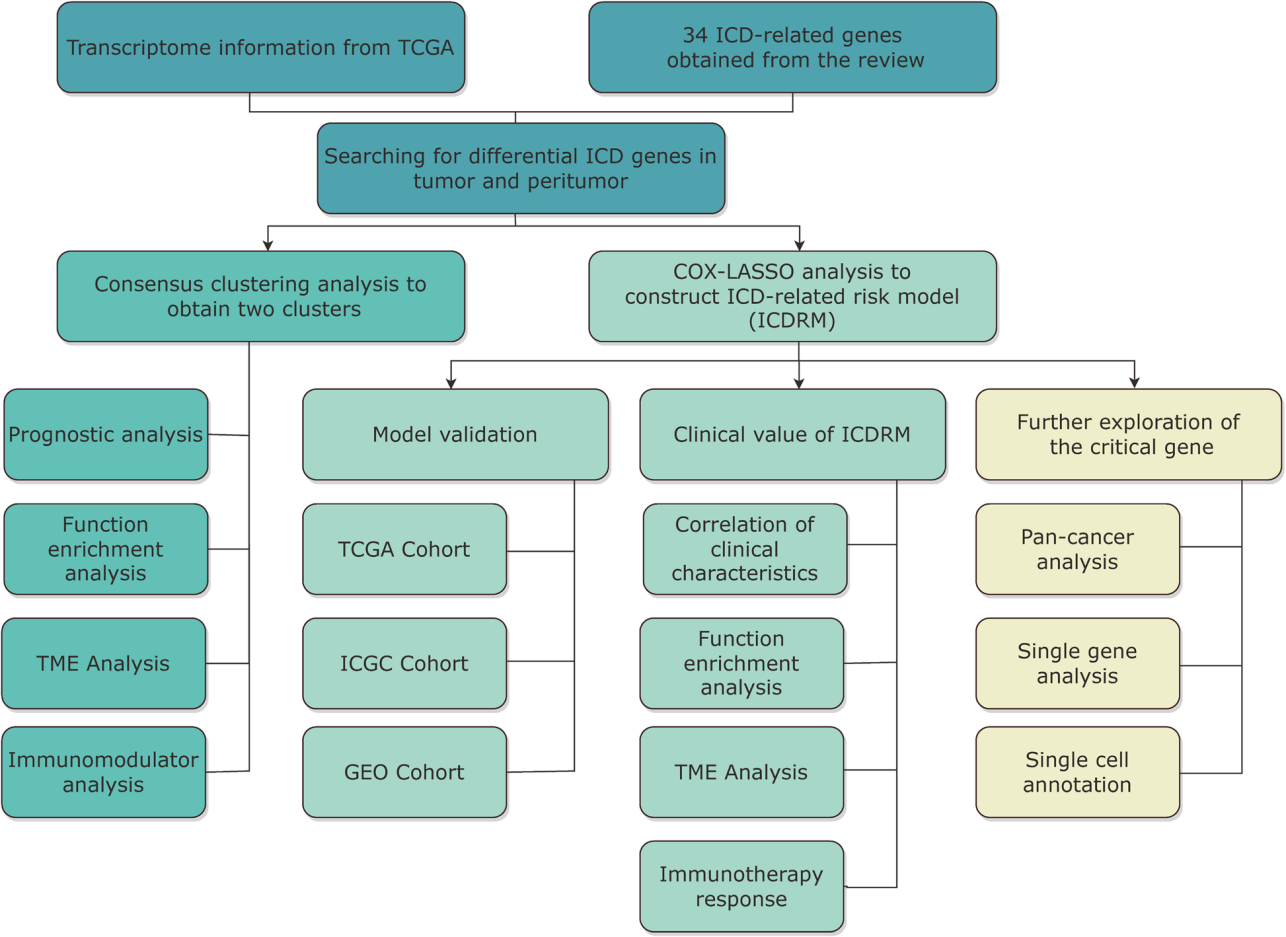
Landscape of this study workflow

A total of 34 ICDs were identified through literature [8]. Sequencing data and clinical information for HCC samples were collected from publicly available databases. From the TCGA database (https://portal.gdc.cancer.gov/), 374 HCC specimens were sequenced, and clinical information matched the training set. For the validation set, 115 HCC specimens from the GEO database(GSE76427) (https://www.ncbi.nlm.nih.gov/geo/) and 273 HCC specimens from the ICGC database (https://dcc.icgc.org/) [9]. We drew a flow chart of the entire research procedure (Fig. 1). Ten patients with HCC were recruited from Fujian Provincial Hospital with the approval of the ethics committee, and the surgically removed tissue was rapidly cryopreserved using − 80 °C liquid nitrogen.

### Immunohistochemistry (IHC) staining

HSP90AA1 (Heat Shock Protein 90 Alpha Family Class A Member 1) protein levels were analyzed in tumor and peritumor using standard immunohistochemical methods. Briefly, slides were coated with primary antibody against HSP90AA1 (mouse ID:ab79849, Abcam) and incubated overnight at 4 °C, then incubated with secondary antibody for 30 min at room temperature. The slides were stained with DAB solution for 10 min, and the intensity of HSP90AA1 staining was scored, shown below. 0, 1, 2, 3 representing negative, weak, moderate, and strong; The evaluation of the tumor cell positivity rate was as follows. 1, 2, 3, 4 representing 0-25%, 26-50%, 51-75% and 76-100%. The staining intensity was multiplied by the positive rate score to obtain the IHC score. Two pathologists reviewed sections.

### Hepatocellular carcinoma cell lines

Fujian Provincial Hospital provided the THLE-2 hepatocyte line and the HepG2, Hep3B, and Huh-7 cell lines. Cells were frozen in liquid nitrogen, stored in a humidified incubator (5% CO2, 37 °C), DMEM medium containing 1% antibiotics (100 U/ml cyanide and 100 ug/ml streptomycin sulfate, Sigma, USA), and 10% heat-inactivated fetal bovine serum (FBS, Gibco, USA) was used for culture.

### Plasmid construction and cell transfection

We constructed and maintained the BAX knockout plasmid (BAX-sh) in our laboratory (Fujian, China). The THLE-2 cell line was used as an internal control. BAX-sh was transfected into human HCC cells using LipofectamineTM2000. Handle the HSP90AA1 (Heat Shock Protein 90 Alpha Family Class A Member 1) protein similarly.

### RNA isolation and qRT-PCR

RNA was isolated from HCC cells and tissues and matched non-cancerous tissues using the TRIzol Reagent (Thermo Fisher Scientific, Waltham, MA, USA) following the manufacturer’s protocol. Reverse transcription was performed using the PrimeScript RT Reagent Kit (Takara, Dalian, China). Real-time PCR reactions were conducted using the StepOnePlus™ Real-Time PCR System (Thermo Fisher Scientific, MA, US) with temperature cycling settings as recommended by the manufacturer. The sequences of primers can be found in Supplementary Table S 1.

### Colony formation assay

The cancer cells were placed in 12-well tissue culture plates for one week, thus allowing the cells to form colonies. Specimens were treated with 10% neutral formalin. Staining was performed with 0.5% crystal violet solution, and the dye extract used 10% acetic acid. The visible colonies were quantified by counting them under a light microscope. The number of colonies was measured in triplicate wells for each treatment group.

### CCK8 assay

Cell Counting Kit-8 (CCK8) was used to analyze cell viability. Cells were seeded in 100 µL medium at a 5 × 10^3^/well density and cultured in 96-well microplates (Corning, USA). Subsequently, treatment of cells with different concentrations of Tan-I. After 24 h of processing, add 10 µL CCK-8 reagent to each well and incubate for 2 h. Experiments were all performed with three replications. The absorbance of the cells at 450 nm was read using a microplate reader. The absorbance was then used to quantify the proliferation of cells.

### Wound healing

We used 24-well plates to grow the wound-healing cells. Use a sterile instrument for scratching the cells to test wound healing. The healing of the cell scratches was observed by inverted microscopy, photographed, and the distance of cell migration was recorded at two-time spots, 0 and 24 h. The specific procedures were consistent with a previous study [10]. Photographs were taken under phase-contrast microscopy (Olympus, Tokyo, Japan).

### Characterization of ICD clusters

Consensus clustering is an effective dimensionality reduction method, an algorithm for identifying the members of a typology and their number in a data set [11, 12]. Patients were classified by expression data of ICDs using a consensus clustering method [13]. To assess the clinical value of ICD clusters, we correlated the clusters with other clinical characteristics (e.g., Age, Gender, TNM stage including T, N, and M) were compared [14]. Among the cohorts, survival differences were compared using Kaplan-Meier survival plots [15].

### Enrichment analysis and immune landscape of different clusters

Differentially expressed genomes (DEGs) are calculated in R using the package “limma”. The enrichment of genomes based on DEGs is analyzed using the R package “clusterProfiler“ [16]. Normalized enrichment scores (NES) for pathway and functional annotations were calculated according to the gene set variation analysis method of the R package “GSVA”, and the NES-based heat map showed the biological functional differences between different clusters [17]. Human genome reference documents were downloaded from the MSigDB database (http://www.gsea-msigdb.org/gsea/msigdb), followed by gene set variance analysis (GSVA) and gene set enrichment analysis (GSEA). Significantly different entries were obtained by setting *P* < 0.05 and FDR < 0.25. Meanwhile, Gene Ontology (GO) and Kyoto Encyclopedia of Genes and Genomes (KEGG) enrichment analysis was used to explore the signaling pathways involved and the functions possessed by the DEGs, and which functions or pathways contribute most to the changes in phenotype. Various algorithms, including GSEA, single sample GSEA (ssGSEA), and CIBERSORT, were used to quantify the level of immune infiltration in TCGA [18]. We compared differences in the expression of immune-related genes between clusters, And the set of immune-related genes was acquired through literature [19].

### Analysis of somatic mutations and copy number variations

We downloaded somatic mutations and copy number variations (CNV) data from TCGA. Differences in mutant forms between clusters were analyzed through the R package “maftools“ [20], and the OncoPrint map of different clusters of mutations was generated with the R package “ComplexHeatmap“ [21].

### ICDRM construction and characterization

A differential expression analysis using the R package “limma” was used to screen ICDs differentially expressed between tumors and peritumoral, followed by a Lasso-Cox regression analysis. The ICDRM risk score calculation method is as follows:

$$Risk\;Force\;=\;\sum_{i=1}^n\;Coe\;genei\;\ast\;Exp\;of\;genei$$

Coe genei is the abbreviation of gene coefficient in this research, and the Exp of genei is the expression of genes. We randomly assigned the TCGA-HCC cohort samples to the risk-scoring model in a 1:1 ratio, differentiating between training and test sets. The ICGC and GEO datasets were used as external data to assess the reliability and stability of the model prediction of patient survival at 1, 3, and 5 years. Factors associated with prognosis were obtained by regression analysis combining ICDRM risk score and other clinical characteristics, and forest plots of clinical prognostic factors were constructed. Nomograms were built through the R package “RMS” using factors associated with prognosis. Calibration plots were plotted to show the agreement between the 1, 3, and 5 years endpoint events predicted by the Nomogram and the actual outcome [10, 22].

### Immunotherapy and drug prediction

Immunotherapy is widely used in HCC, and to better differentiate patients treated with immunotherapy, The Cancer Imaging Archive (TCIA) database (https://tcia.at/home) is used to help predict immunotherapy response [23]. Gene expression data of the TCGA-HCC cohort were uploaded to TCIA, and the immunophenotype score (IPS) of each sample was downloaded. The method used to compare IPS differences between high and low-risk subpopulations was the Wilcox test. Notably, IPS is a prognostic indicator to predict the effect of anti-PD-1 and anti-CTLA-4 therapy [24, 25]. To research potential drugs for the treatment of HCC patients, we computed DEGs between high-risk and low-risk subpopulations (*P* > 0.05, |log2FC| > 1) using the R package “limma”. The difference in semi-inhibitory concentrations (IC50) between common chemotherapeutic drugs and targeted drugs in high and low-risk subpopulations was calculated by the R package “pRRophetic“ [26]. The therapeutic efficacy of different drugs in different subpopulations was compared [27]. The first 150 upregulated and down-regulated DEGs were submitted to the cMAP database (https://clue.io/) [28] and drugs with a | median tau score| > 80 were set as drugs effective for HCC treatment. The value of ICDRM in predicting therapeutic drug candidates is achieved by the two prediction methods described above. PubChem [29] (https://pubchem.ncbi.nlm.nih.gov/) presents the Structures of drugs in three dimensions.

### Further investigated the key gene of ICDRM

SangerBox [30] (http://vip.sangerbox.com/) demonstrated genes’ expression and prognostic, predictive power in pan-cancer. We used the LinkedOmics [31] database (http://www.linke-domics.orglo-gin.php) to identify co-expressed genes for the critical gene in TCGA. Gene enrichment analysis of GO, KEGG, and tissue using the Metascape [32] database (https://metascape.org). Correlations between genes and immune cells were analyzed via the TIMER [33] database (https://cistrome.shinyapps.io/timer/). Gene annotation at the single-cell level is presented by the Tumor Immunization Single-Cell Center (TISCH) [34]website (http://tisch.comp-genomics.org/). The differentiation trajectories of critical cell fractions and their marker genes are presented through the TIGER [35] database (http://tiger.canceromics.org/). Regarding immunotherapy, the ICBatlas [36] database (http://bioinfo.life.hust.edu.cn/ICBatlas/) was used to explore differences in gene expression in different treatment cohorts.

### Statistics analysis

This study used R [31] software (version 4.1.3) for statistical analysis and graphing. GraphPad Prism 8.0 and SPSS 23.0 were also applied for statistical analysis. Differences between the indicated groups were compared using the Student’s t-test and one-way analysis of variance (ANOVA) followed by Fisher’s least significant difference (LSD) test. Correlations were evaluated by Pearson correlation analysis. A *P*-value < 0.05 was considered to indicate a statistically significant result (* *P* < 0.05; ** *P* < 0.01; *** *P* < 0.001). Other related R packages are free for download from the Bioconductor or the R website [37, 38].

## Results

### Consensus clustering identified two ICD-associated clusters

**Fig. 2:**
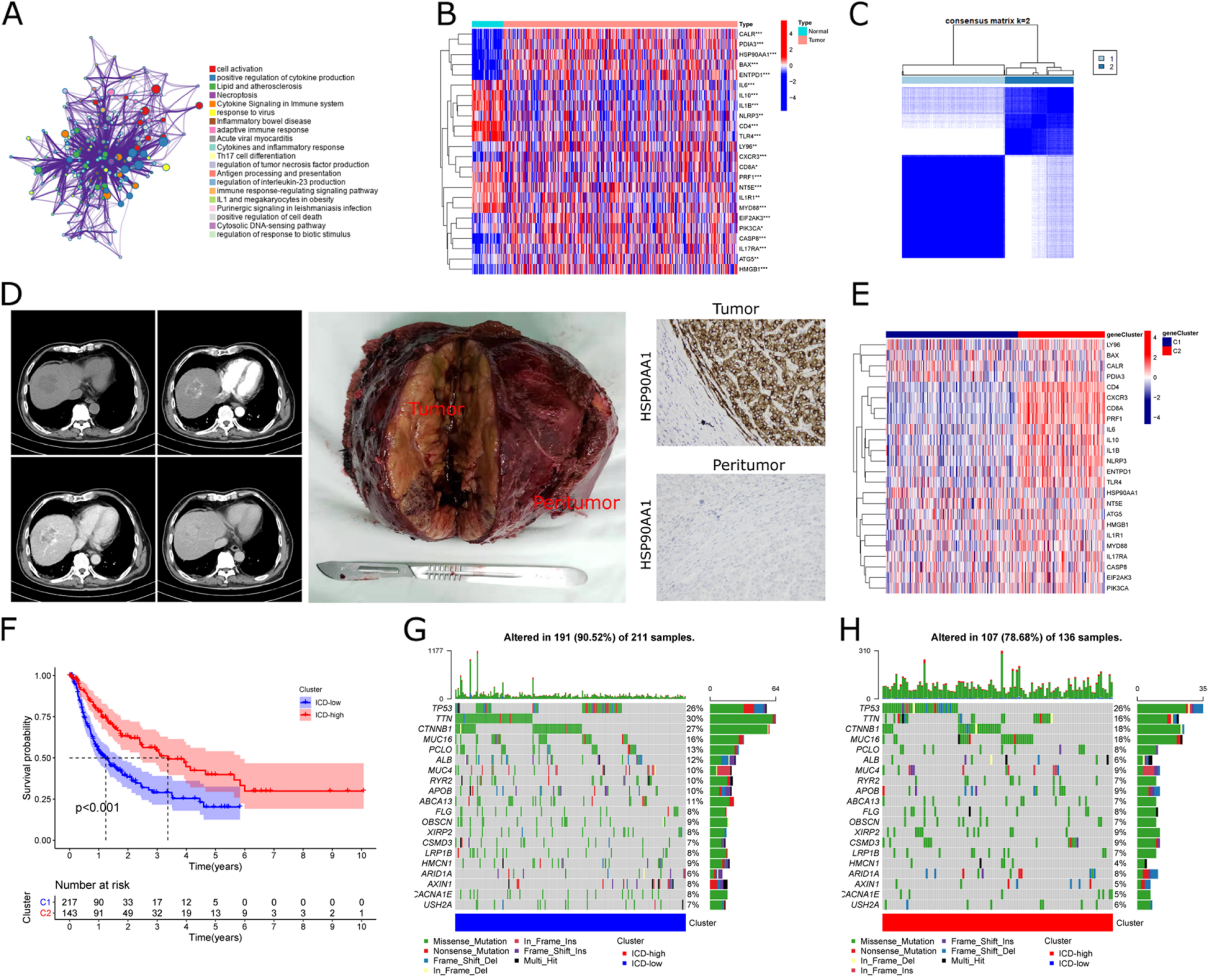
Genotyping of ICD-related genes. **A** Enriched ontology clusters of ICD genes analyzed by Metascape, colored by cluster-ID. **B** Heatmap shows 34 ICD gene expression differences among tumor and peritumor samples in TCGA. **C** Total of 374 HCC patients was identified into two clusters according to the consensus clustering matrix (k = 2). **D** Validation of HSP90AA1 expression in clinical tumor tissues by representative images of hepatocellular carcinoma and IHC of tumor and peritumor tissue. **E** Heatmap shows ICD-related gene expression differences between ICD-low and ICD-high clusters in TCGA. **F** Kaplan-Meier survival curves for the two clusters (*P* < 0.001). **G**, **H** Comparison of the mutation landscape between ICD-low and ICD-high clusters

We used the Metascape database to visualize the extensive association between ICDs, and enrichment analysis showed an association with signaling pathways of multiple immune-related pathways (Fig. 2A). We also analyzed the differential expression of these genes in tumor and peritumor tissues. Most ICDs showed differential expression (Fig. 2B), and the set of differentially expressed ICDs was used for further analysis. We performed immunohistochemical experiments on the significantly differentially expressed gene, HSP90AA1, to verify the differential gene expression in tumor and peritumor tissues. The levels of HSP90AA1 in tumor and peritumor tissues were compared in HCC patients by immunohistochemical (IHC) staining, which showed that HSP90AA1 was overexpressed in tumor tissues (Fig. 2D).

TCGA-HCC cohorts were clustered into clusters C1 and C2 based on unsupervised clustering methods (Fig. 2C). In clusters C1 and C2, gene expression for ICDs is shown as a heatmap (Fig. 2E). According to Kaplan-Meier (KM) analysis, in terms of overall survival(OS), there was a significant difference among C1 and C2 clusters (*P <* 0.001) (Fig. 2F). Overall, the expression of ICDs was high in cluster C2 and low in cluster C1. Therefore, we defined cluster C1 as ICD-low and C2 as ICD-high.

In addition, the ICD-low cluster is associated with a poor prognosis, whereas the ICD-high clusters have a better prognosis. Regarding the differences in somatic mutations between the two clusters (Fig. 2G H), we observed no difference between the two clusters of genes that were altered most frequently, but the relative frequencies were different. For example, the frequency of TNN mutations in the ICD-low clusters is 30%. In contrast, the frequency of TNN mutations in the ICD-high clusters is 16%, demonstrating that the essence of the difference in ICD clusters is the difference in copy number rather than gene mutations. Then we examined the differences in copy number variation (CNV) mutations between the two clusters (Fig. S 1A-B) and found a notable difference in the genomic background and expression levels of ICDs between ICD-low and ICD-high clusters. Our results suggest that CNV may have a regulatory role in the expression of ICD clusters.

**Fig. 3:**
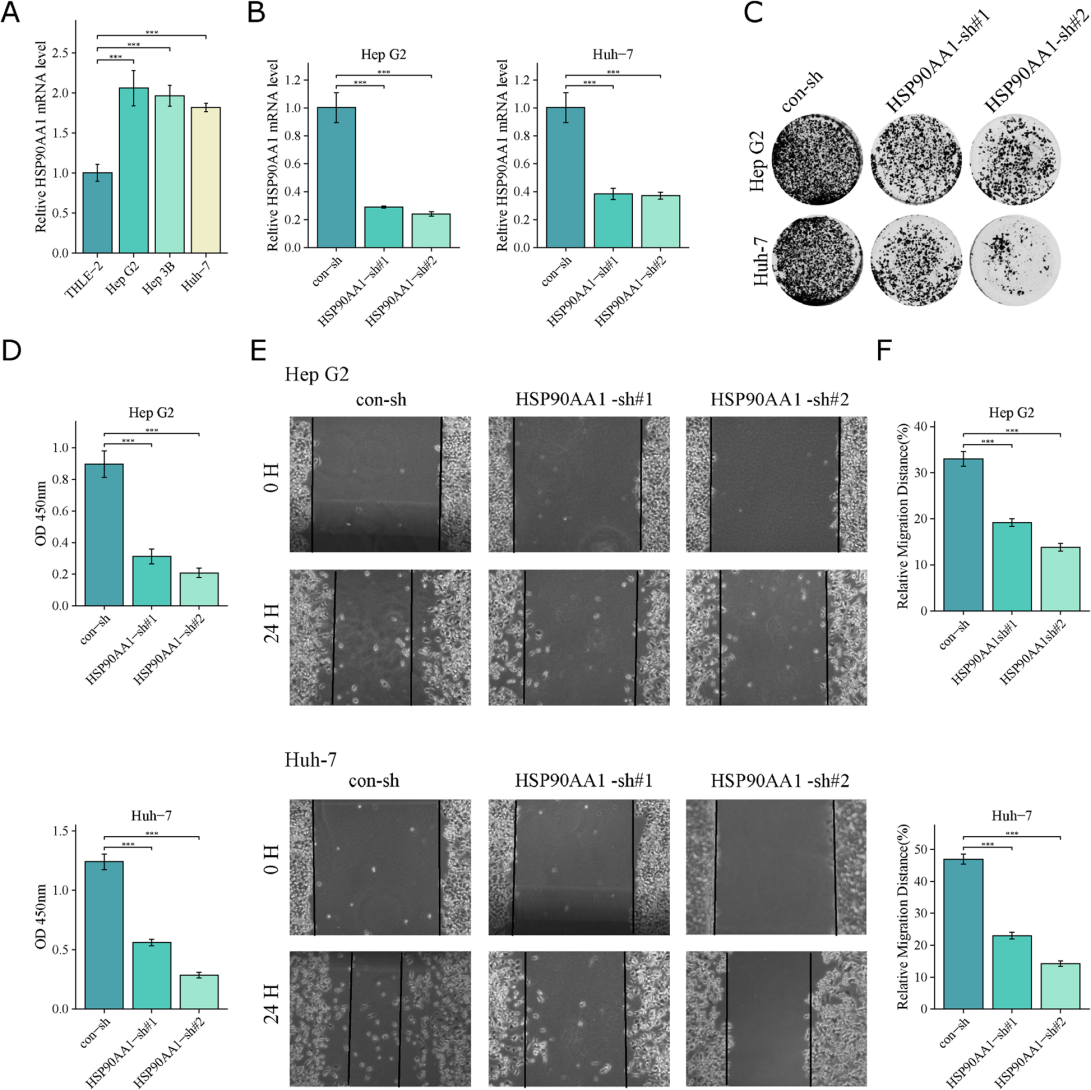
Expression of HSP90AA1 in HCC cells and its function.** A.** mRNA levels of HSP90AA1 in THLE-2 and HCC cells. **B** mRNA levels of HSP90AA1 in HepG2 and Huh7 HCC cells after HSP90AA1 was knocked down. **C**, **D** A colony formation assay was used to explore the function of HSP90AA1 in HCC cells. Their representative images are shown in **C**. **E**, **F** Knockdown of HSP90AA1 inhibits HCC cell migration. Wound healing assays were used to assess the migration of HepG2 and Huh7 cells after the HSP90AA1 knockdown. Representative images are shown in **E** (* *P* < 0.05, ** *P* < 0.01, ***P < 0.001). All experiments were repeated at least three times

In addition, various in vitro experiments were conducted to assess the role of HSP90AA1. Figure 3 A shows the expression of HSP90AA1 in HCC cell lines and normal hepatocytes (THLE-2); the mRNA expression of HSP90AA1 was found to be increased in HCC cells compared to THLE-2 cells. In addition, we knocked down the expression of HSP90AA1 in HepG2 and Huh7 cells (Fig. 3B). Knockdown of HSP90AA1 inhibited the growth (Fig. 3C and D) and migration (Fig. 3E F) of HCC cells. The oncogenic role of HSP90AA1 in HCC has been reported in previous studies [39–41], and our results are the same as the previous study.

Furthermore, we also did a functional verification of BAX, the most differentially significant ICD-associated gene in HCC. The role of BAX in HCC has been reported in previous studies [42–44]. Figure S 2A shows the expression of BAX in HCC cell lines and normal hepatocytes (THLE-2); the mRNA expression of BAX was found to be increased in HCC cells compared to THLE-2 cells. In addition, we knocked down the expression of BAX in HepG2 and Huh7 cells (Fig. S 2B). Knockdown of BAX inhibited the growth (Fig. S 2C-D) and migration (Fig. S 2E-F) of HCC cells.

### Enrichment analysis and Immune landscape of ICD clusters

**Fig. 4:**
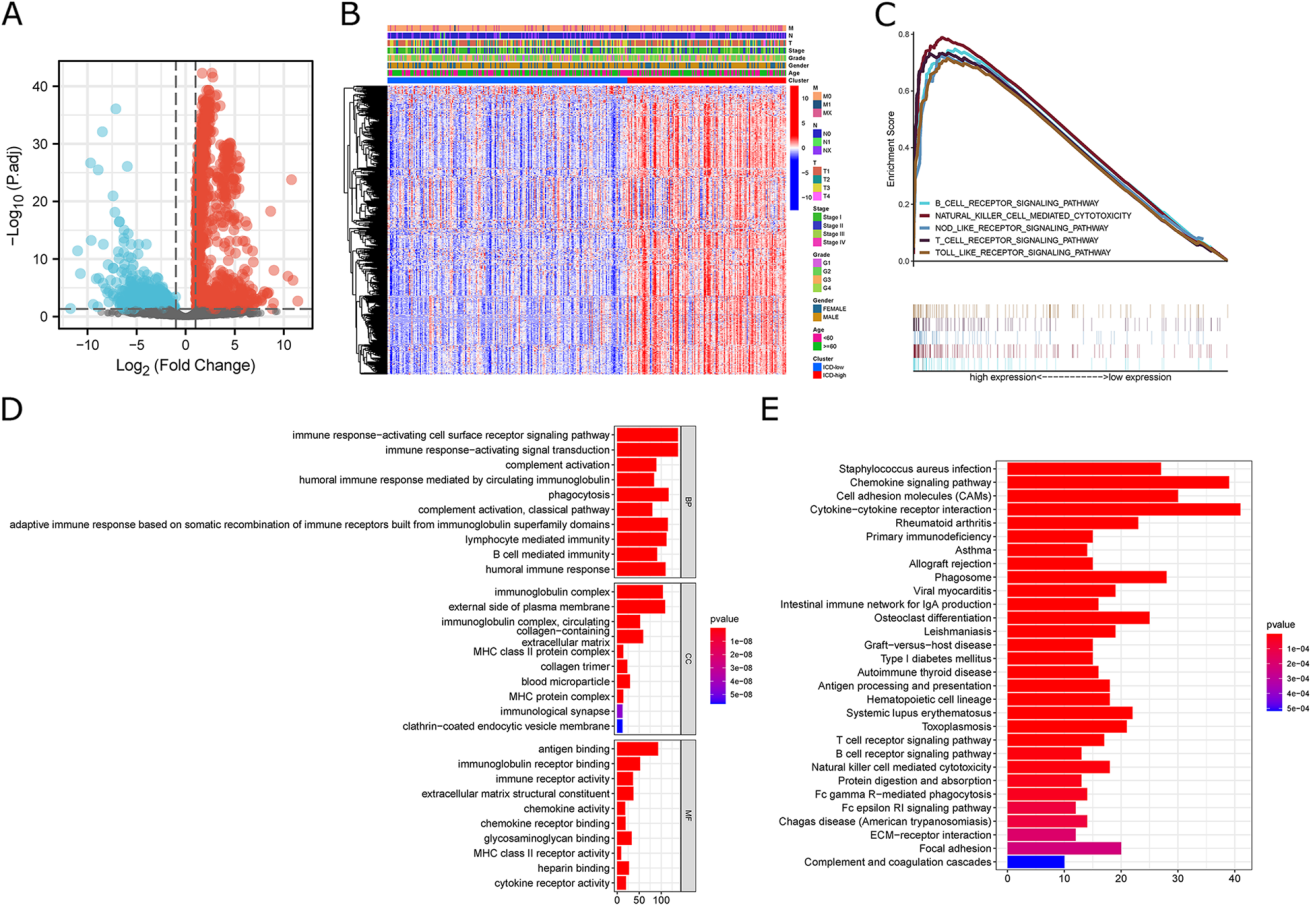
Identification of DEGs and signal pathways in different clusters.** A** Volcano plot presents the DEGs between ICD-low and ICD-high clusters with a threshold of |log2 FC| > 1 and *P* < 0.05 in TCGA. **B** Heatmap shows the relationship between subpopulations and clinical features. **C** GSEA analysis shows the signal pathways between ICD-low and ICD-high clusters. **D**, **E** Bar Chart presents the GO and KEGG enrichment analysis of DEGs.

We used the R package “limma” for further analysis of the TCGA-HCC cohort, setting |logFC|>1.0 and *P* < 0.05. There were 678 DEGs calculated between ICD-low and ICD-high clusters (Fig. 4A, Supplementary Table S 2), and the Heatmap showed no significant association between ICD clusters and clinical features (Fig. 4B). GSEA enrichment analysis revealed that DEGs upregulated in ICD-high groups were markedly enriched in immune-associated pathways, including T cell, B cell, and NK cell signaling pathways (Fig. 4C), and the other pathways are shown in Supplementary Table S 3. In addition, GO enrichment analysis shows the biological process (BP) of upregulated DEGs in the ICD-high clusters were associated with immune response activation; cellular component (CC) is mainly related to the outer side of the plasma membrane; cellular function (MF) is primarily related to antigen binding, immunoglobulin receptor binding, and immune receptor activity (Fig. 4D). KEGG enrichment analysis revealed ICD-high clusters associated with immunization processes such as natural killer cell-mediated cytotoxicity, T cell receptor signaling pathway, and B cell receptor signaling pathway (Fig. 4E).

**Fig. 5:**
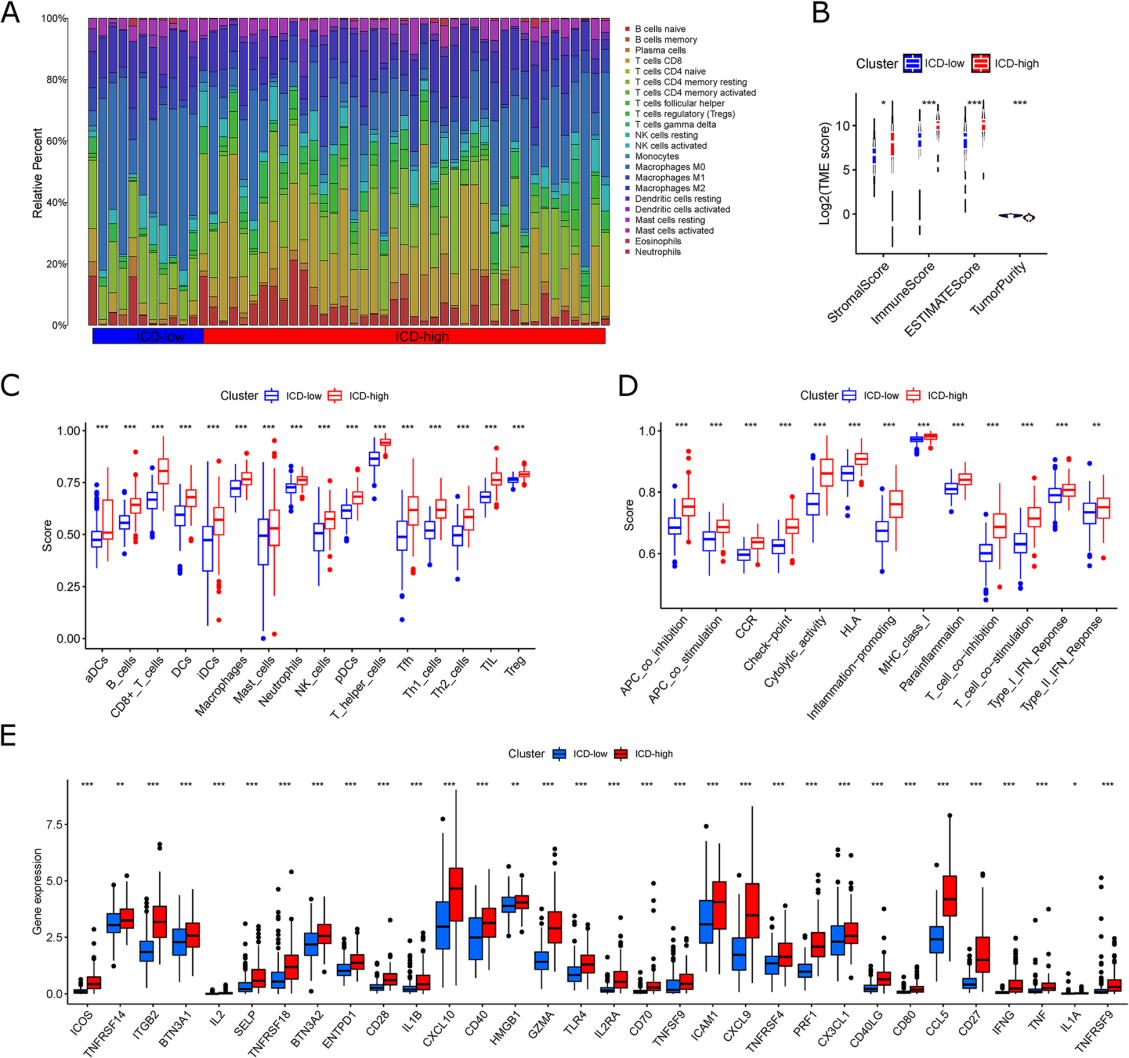
The immune landscape of ICD-low and ICD-high clusters.** A** Differences in the immune cell infiltration using the CIBERSORT method. **B** Differences in the estimations of a stromal score, immune score, and tumor purity score using the CIBERSORT method. **C** Differences in the immune cell subtypes using the ssGSEA method. **D** Differences in the immune cells-related functions using the ssGSEA method. **E** Differences in the expression of immune stimulatory-related genes using the ssGSEA method

CIBERSORT and ssGSEA algorithms were used to compare the infiltration of immune cells between the two clusters based on the TCGA-HCC cohort. First, the infiltration component of 22 immune cells for each sample was calculated using the CIBERSORT algorithm. The percentages of NK-activated cells, CD4 memory-activated T cells, and CD8 T cells, representing immune activation, were relatively increased in the ICD-high clusters; the percentages of M2 macrophages, CD4 memory-resting T cells, and NK-resting cells, representing immunosuppression, were relatively decreased in the ICD-high clusters (Fig. 5A). The R package “Estimate” generates a tumor stromal score and an immune score, which are combined to produce an index called the “Estimate Score” and a score based on the Estimate algorithm to infer tumor purity. In clusters with ICD-high, stromal and immune scores are high. Still, tumor purity is low (Mann-Whitney u-test) (Fig. 5B). Comparative analysis of immune cell infiltration using the ssGSEA algorithm revealed that the ICD-high clusters was significantly higher than ICD-low clusters in all immune cell infiltration levels and immune function scores (Fig. 5C and D). In addition, we performed a comparison of the expression of genes associated with immune activation between the two clusters, and the expression was significantly upregulated in the ICD-high clusters (Fig. 5E), as were the results observed for the differential expression of RNA modification-related genes, MHC, chemokines, and receptor genes (Fig. S 3A-D). The above analysis results suggest that the ICD-high clusters represent immune-activated hot tumor phenotypes, while the ICD-low clusters represent immune-suppressed cold tumor phenotypes.

### ICDRM building and validation

**Fig. 6:**
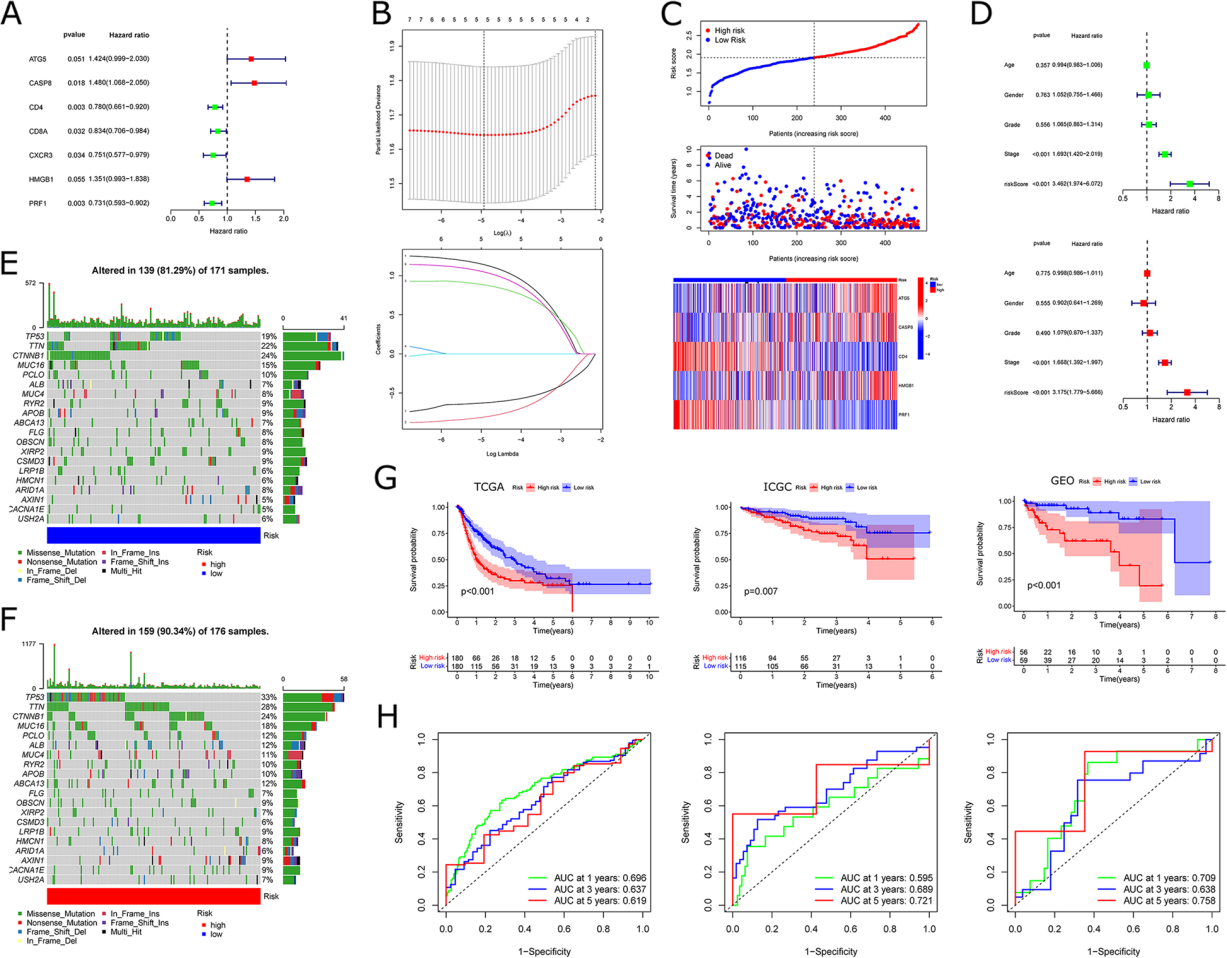
Construction and validation of ICDRM.** A** Univariate Cox analysis evaluates the predictive value of ICDRM in terms of OS. **B** Lasso-Cox analysis identified 5-genes most associated with OS in TCGA. **C** Risk scores distribution, survival status of each patient, and heatmap of prognostic 5-genes signature in TCGA. **D** Univariate and multivariate Cox analyses evaluate the independent prognostic value of ICDRM in TCGA. **E**, **F** Comparison of the mutation landscape between subpopulations with high and low-risk scores. **G** Kaplan–Meier analyses demonstrate the prognostic significance of ICDRM in the TCGA, ICGC, and GSE76427 datasets. **H** AUC of time-dependent ROC curves verified the predictive performance of ICDRM in TCGA, ICGC, and GSE76427 datasets

To obtain the characteristic genes most associated with prognosis, we screened 7-ICD genes that had an association with OS in HCC patients by univariate COX analysis (setting *P* < 0.1); 5-ICD-associated genes were identified to build ICDRM after incorporating variables into the LASSO regression model (Fig. 6A and B). Subpopulations at high-risk had much higher mortality states than those at low risk. ATG5, CASP8, and HMGB1 were highly expressed in the high-risk subpopulations, whereas CD4 and PRF1 expressed were lower (Fig. 6C). In a multivariate Cox analysis, the poor prognosis was significantly correlated with tumor stage and ICDRM. Multifactorial analysis showed that tumor stage and ICDRM risk score were independent risk factors of HCC (Fig. 6D). Different somatic mutations and CNV were constructed between high-risk and low-risk subpopulations based on the TCGA-HCC cohort (Fig. 6E-F). Our findings revealed differences in the genomic background and expression levels of ICDs between Risk-low and Risk-high subpopulations, suggesting the involvement of somatic mutations and CNV in HCC tumorigenesis (Fig. S 4A, B). The top 20 driver genes that were altered most frequently in both subpopulations were similar. However, the high-risk subpopulations had significantly more changed cell copy numbers than the low-risk subpopulations. Kaplan-Meier survival analysis indicates that high-risk subpopulations are linked to poorer prognosis, with statistically significant differences; the ICGC and GEO datasets demonstrated the effectiveness of ICDRM (Fig. 6G). In the TCGA cohort, the survival AUC was 0.696, 0.637, and 0.619 at 1, 3, and 5 years; in the ICGC cohort, the survival AUC was 0.595, 0.689, and 0.721 at 1, 3, and 5 years; and in the GSE76427 cohort, the survival AUC was 0.709, 0.638, and 0.758 at 1, 3, and 5 years (Fig. 6H).

**Fig. 7:**
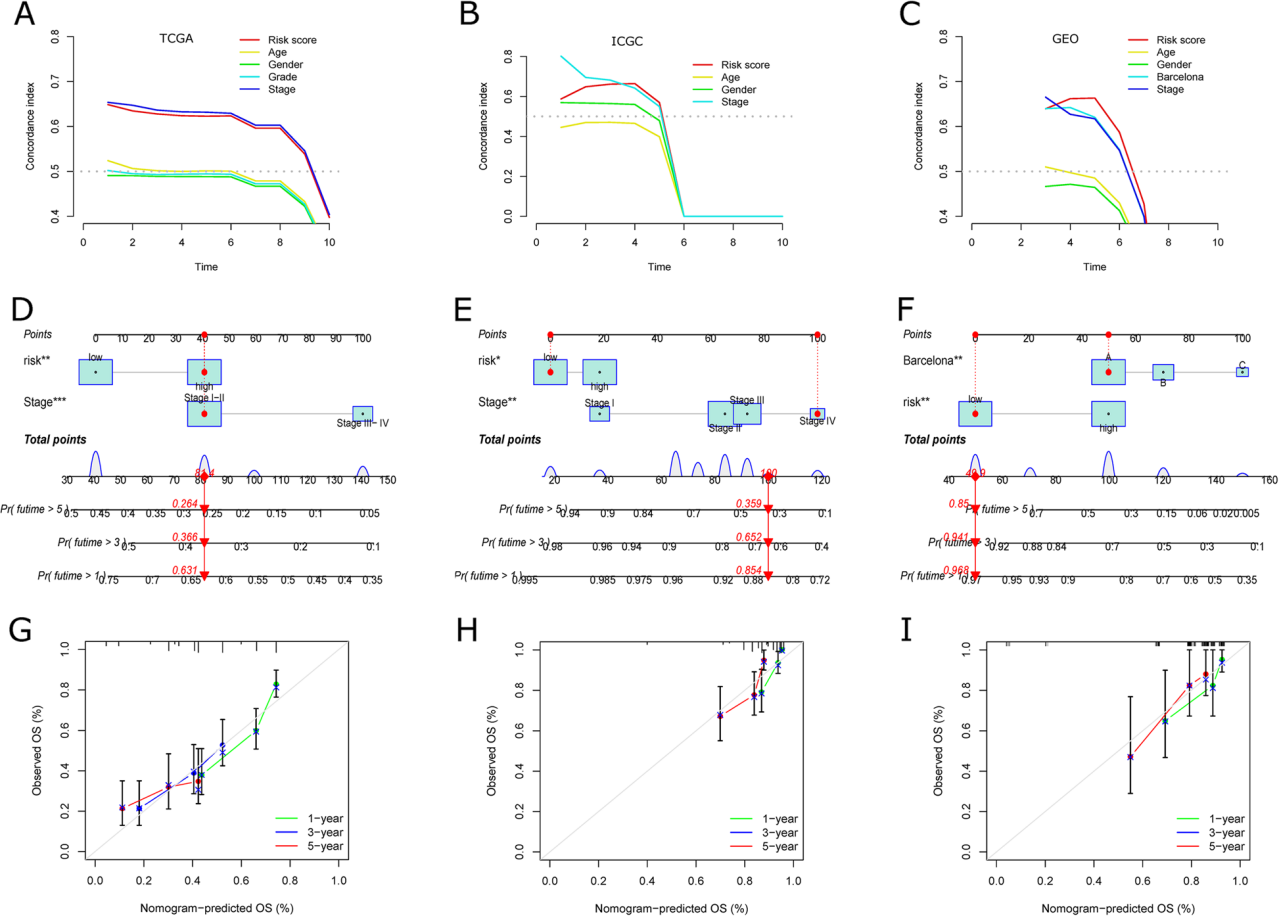
Prognostic value of ICDRM in TCGA, ICGC, and GEO datasets.** A-C** Concordance index (C-index) of ICDRM. **D-F** Construction of Nomograms with the ICDRM and tumor stages. **G-I** Calibration curves to evaluate the precision of Nomograms

In the datasets of three different platforms (TCGA, ICGC, and GSE76427), ICDRM had a higher concordance index (C-index) compared to other clinical features (Fig. 7A-C), demonstrating the predictive efficiency of ICDRM. Therefore, we developed a nomogram including tumor staging and risk scores in the TCGA-HCC cohort, the ICGC-HCC cohort, and the GSE76427 dataset as two external validation groups (Fig. 7D-F). Calibration curves were plotted to observe the agreement between the predicted and actual nomogram at 1, 3, and 5 years OS (Fig. 7G and I). The results suggest that ICDRM, including the 5-ICDs mentioned above, has a stable prognostic power.

Somatic mutations data from the TGCA-HCC cohort were used to calculate TMB scores, and the high TMB groups were significantly associated with worse prognosis (*P* = 0.035) (Fig. S 5A), which is consistent with recent studies showing that TMB can be used as a prognostic marker for many kinds of tumors [45]. We compared whether ICDRM predicted overall survival better than TMB in HCC patients. The results showed: patients with a high ICDRM risk score have a worse prognosis (*P* < 0.001), as does the indicator TMB risk score, which is statistically significant. It is interesting to note that the survival curves of the patients with higher TMB were similar to those of the patients with lower TMB in the high-risk (ICDRM) subpopulations. The study’s results suggest that TMB status in high-risk subpopulations does not impact patient overall survival. Thus, these results indicate that ICDRM may have greater prognostic significance than TMB (Fig. S 5B).

### Enrichment analysis and immune landscape of ICDRM subpopulations

**Fig. 8:**
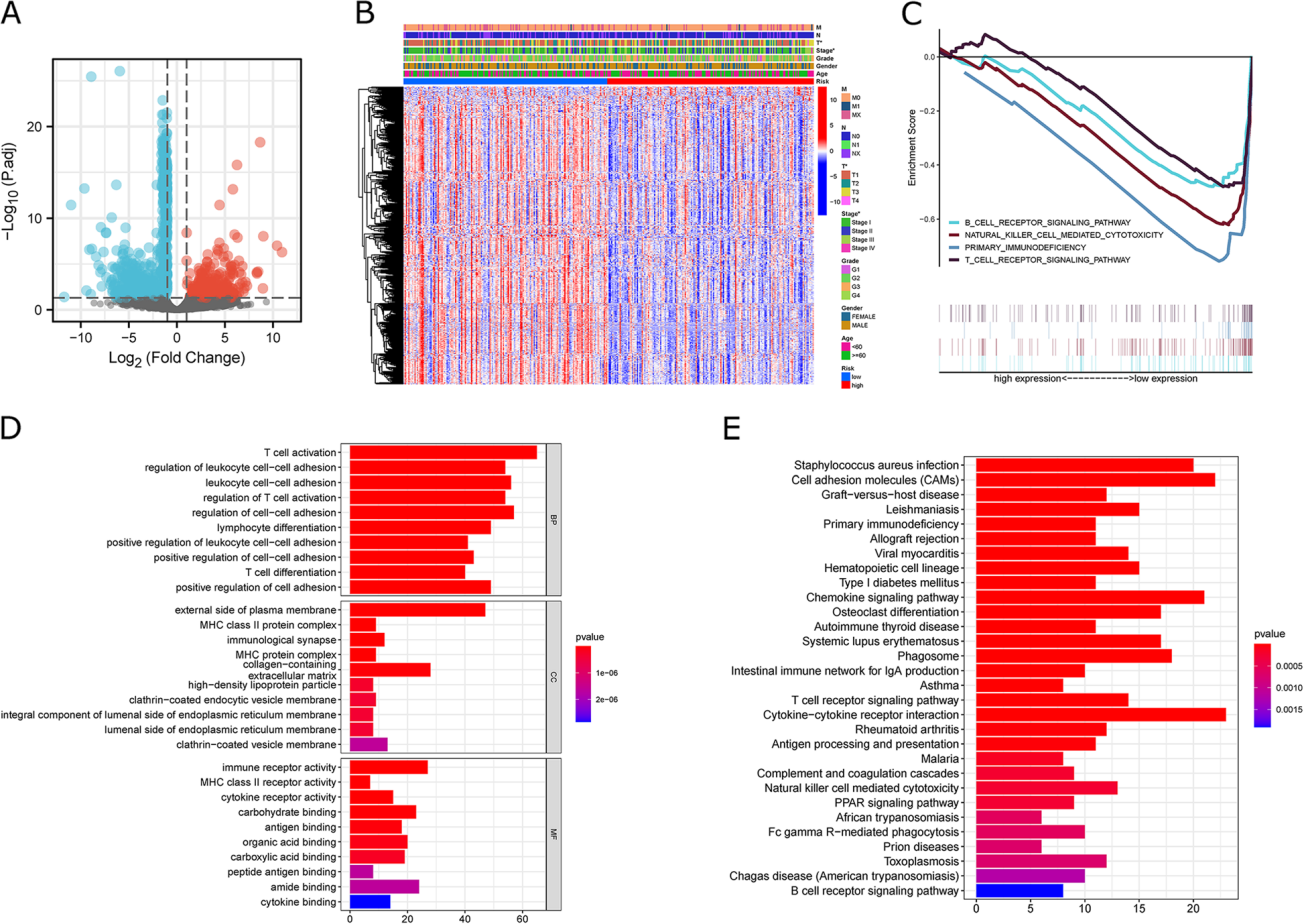
Identification of DEGs and signal pathways in different ICDRM subpopulations.** A** Volcano plot presents DEGs between risk-high and risk-low subpopulations with a threshold of |log2 FC| > 1 and *P* < 0.05 in TCGA. **B** Heatmap shows the relationship between subpopulations and clinical features. **C** GSEA analysis shows the signal pathways between risk-high and risk-low subpopulations. **D**, **E** Bar Chart presents the GO and KEGG enrichment analysis of DEGs.

We used the R package “limma” to further analyze the TCGA-HCC cohort, setting |logFC|>1.0 and *P* < 0.05. There were 1189 DEGs between high-risk and low-risk subpopulations (Fig. 8A, Supplementary Table S 4). Heatmap revealed a remarkable association of ICDRM risk score with tumor stage, especially T stage (Fig. 8B). Several immune-related pathways were notably overrepresented in low-risk subpopulations based on GSEA enrichment analyses, including T-cell receptor signaling pathway, B-cell receptor signaling pathway, and NK-cell receptor signaling pathway (Fig. 8C), and other pathways are shown in Supplementary Table S 5. In low-risk subpopulations, BP of upregulated DEGs was associated with T-cell activation, based on GO enrichment analysis; CC is mainly associated with the outer side of the plasma membrane; MF is primarily associated with immunoglobulin receptor binding, antigen binding, and carbohydrate-binding (Fig. 8D). KEGG enrichment analysis revealed the low-risk subpopulations associated with immune pathways, including NK cell, T cell, and B cell receptor pathways, etc. (Fig. 8E).

**Fig. 9:**
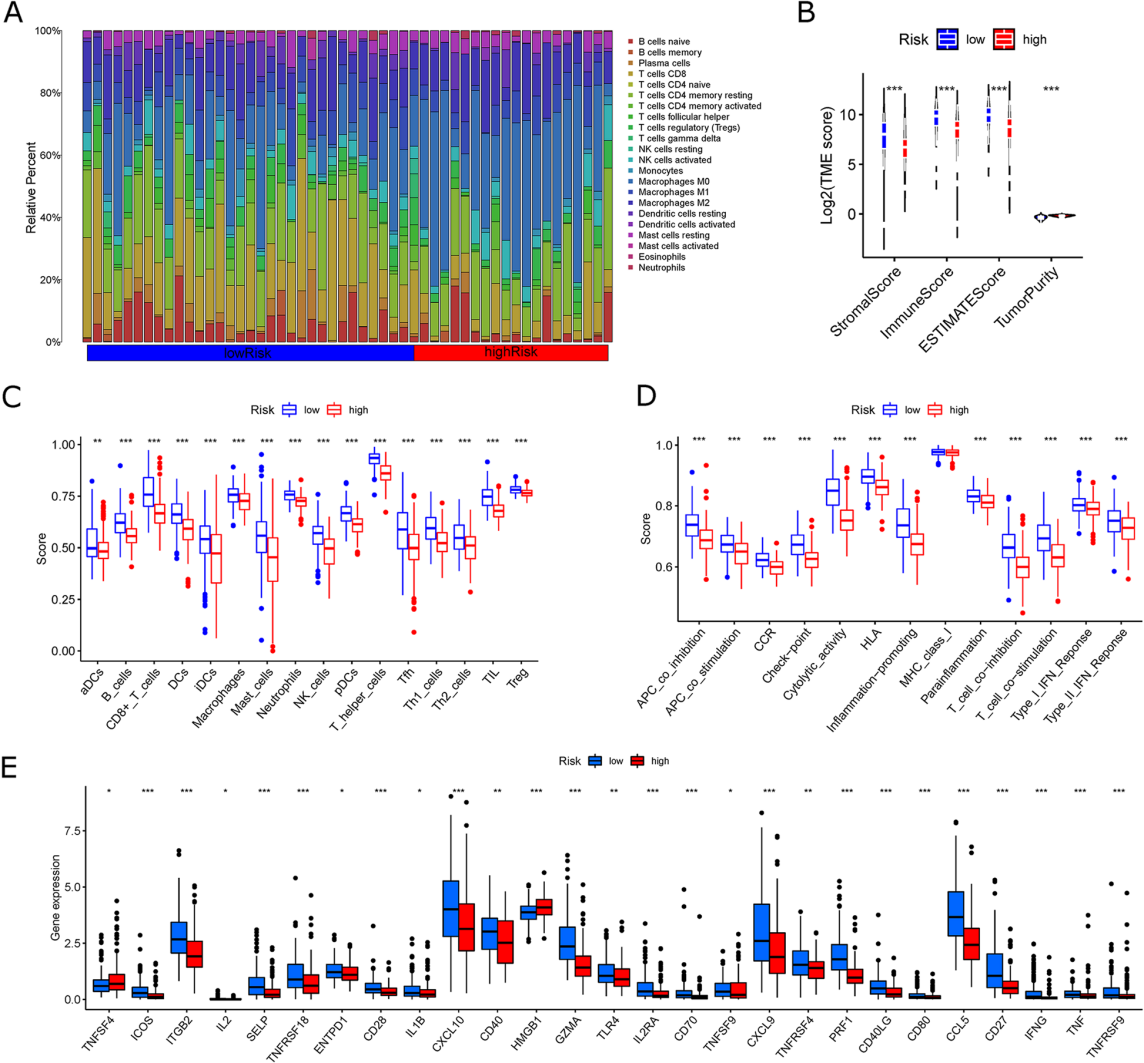
The immune landscape of risk-high and risk-low subpopulations.** A** Differential analysis of immune cell infiltration using the CIBERSORT method. **B** Differential analysis of the estimations for a stromal, immune, and tumor purity score using the CIBERSORT method. **C** Differential analysis of immune cell subtypes using the ssGSEA method. **D** Differential analysis of immune cell-related functions using the ssGSEA method. **E** Differences in the expression of immune stimulatory-related genes

Our study examined the differences in immune cells infiltrating from each subpopulation using both CIBERSORT and ssGSEA algorithms based on the TCGA-HCC cohort [46–48]. Comparative results using the CIBERSORT algorithm indicate that low-risk subpopulations have a proportionally higher number of immune cells with activated immune cell activity. Conversely, the high-risk subpopulations had more immune cells that suppressed immune function (Fig. 9A). Compared to the high-risk subpopulations, the low-risk subpopulations had higher stromal and immune scores and lowered tumor purity scores (Mann-Whitney u-test) (Fig. 9B). In comparisons of the low-risk subpopulation and the high-risk subpopulation using the ssGSEA algorithm, the low-risk subpopulation had significantly greater levels of immune cell infiltrations and immune function scores. (Figure 9C and D). Meanwhile, we constructed a differential heatmap of ICDRM subpopulations and ICD clusters for immune cell infiltration in TCGA (Fig. S 5C). Most patients in the high-risk subpopulations were in the ICD-low clusters, whereas most patients in the low-risk subpopulations were in the ICD-high clusters.

Moreover, the high-risk subpopulations exhibited a cold tumor immunosuppressive phenotype, while the low-risk subpopulations exhibited a hot tumor immunosuppressive phenotype. We also compared the expression differences of genes related to immune activation between the high-risk and low-risk subpopulations. We found most of these genes were markedly upregulated in low-risk subpopulations. In contrast, the opposite trend was observed in the high-risk subpopulations (Fig. 9E). We followed the same results in the differential expression of the following genes: RNA modification-related genes, MHC, chemokines, and receptor genes (Fig. S 6A-D). The above analyses suggest that the ICDRM can discriminate ICD clusters and thus further assess variation in the tumor immune microenvironment of patients with HCC.

### Association of ICDRM with immunotherapy response and drug sensitivity

**Fig. 10:**
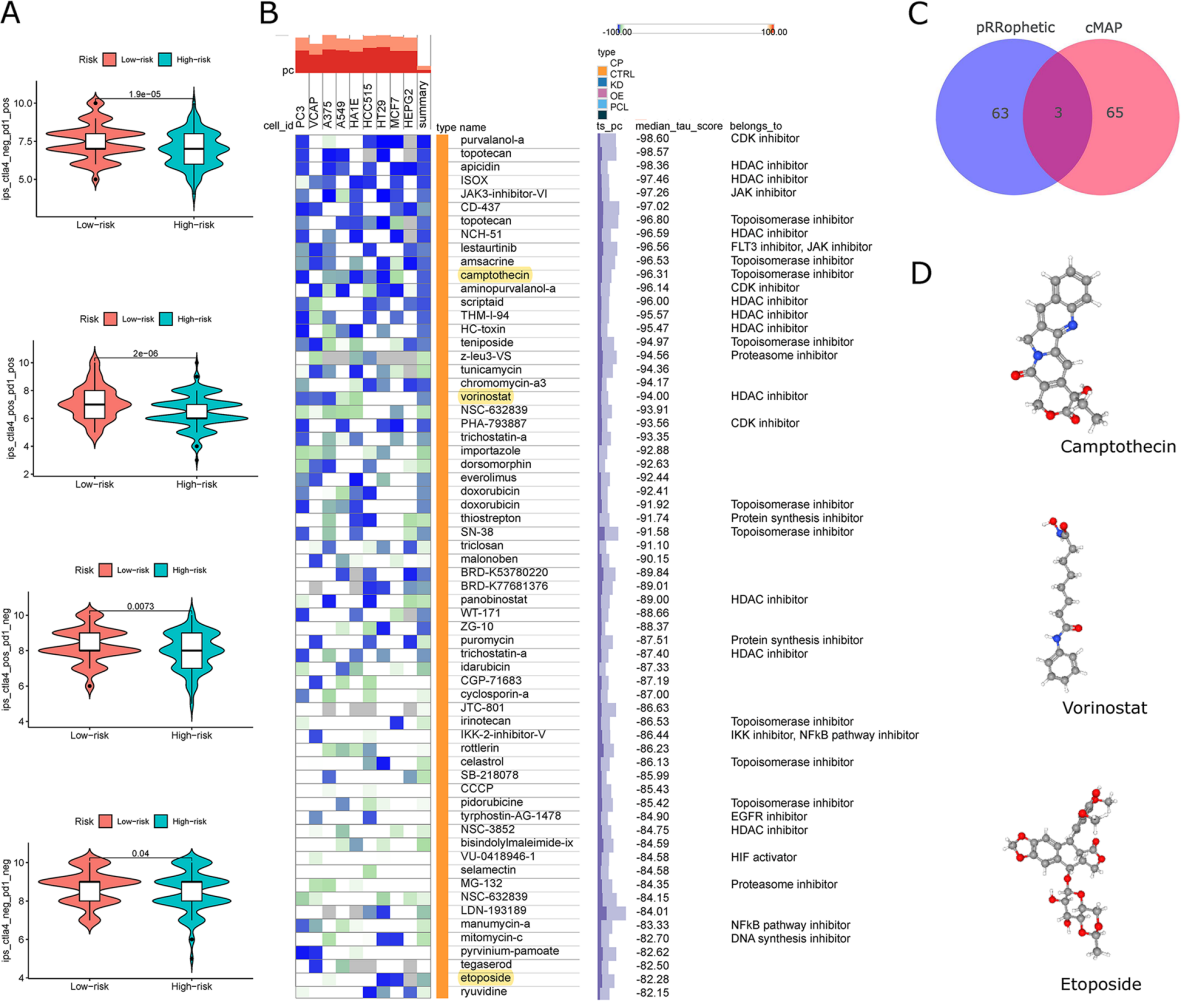
Drug prediction depends on ICDRM.** A** The relationship between PD-1 and CTLA-4 responsiveness differences between risk-low and risk-high subpopulations in TCGA. **B** Drugs predicted by the cMAP database, setting median tau score > -80. **C** Venn diagram for the drugs predicted by the R package “pRRophetic” and the cMAP database. **D** Using the PubChem website to predict the 3D structure of these intersection drugs

Recent studies suggest that IPS based on immunogenicity may help predict response to immunotherapy. The response rates of different subgroups of anti-PD-1 or anti-CTLA-4 antibodies used in the TCIA database were examined. There was a higher level of IPS in low-risk subpopulations. Immunotherapy was likely to be more effective in these subgroups (Fig. 10A). Assessing the value of ICDRM in predicting the sensitivity of chemotherapeutic agents or targeted drugs, 68 drugs effective for HCC treatment were obtained by setting the median tau score > -80 as the cut-off value through the cMAP database (Fig. 10B). Predictions using the R package “pRRophetic” produced 66 commonly used chemotherapeutic or targeted agents more sensitive to the low-risk subpopulations in the TCGA-HCC cohort. The results showed that more drugs were more sensitive to the low-risk subpopulations (Fig. S 7). Taking the intersection of the drugs obtained by the two prediction methods, three common drugs were identified: Camptothecin, Vorinostat, and Etoposide. **(**Fig. 10C, Supplementary Table S 6). Furthermore, we find their 3D structures in PubChem, which may help to design better ICD inducers (Fig. 10D).

We also performed an exhaustive Spearman correlation analysis between ICDRM risk score and immune cells. The results showed that ICDRM was positively correlated with M0 macrophages, activated dendritic cells, and B-cell memory negatively correlated with T cells. In the results, PRF1 was the critical gene of ICDRM, as its correlation and *P* value with immune cells was the most significant (Fig. S 8A, B).

### PRF1 plays a crucial role as a functional mediator of immune regulation

**Fig. 11:**
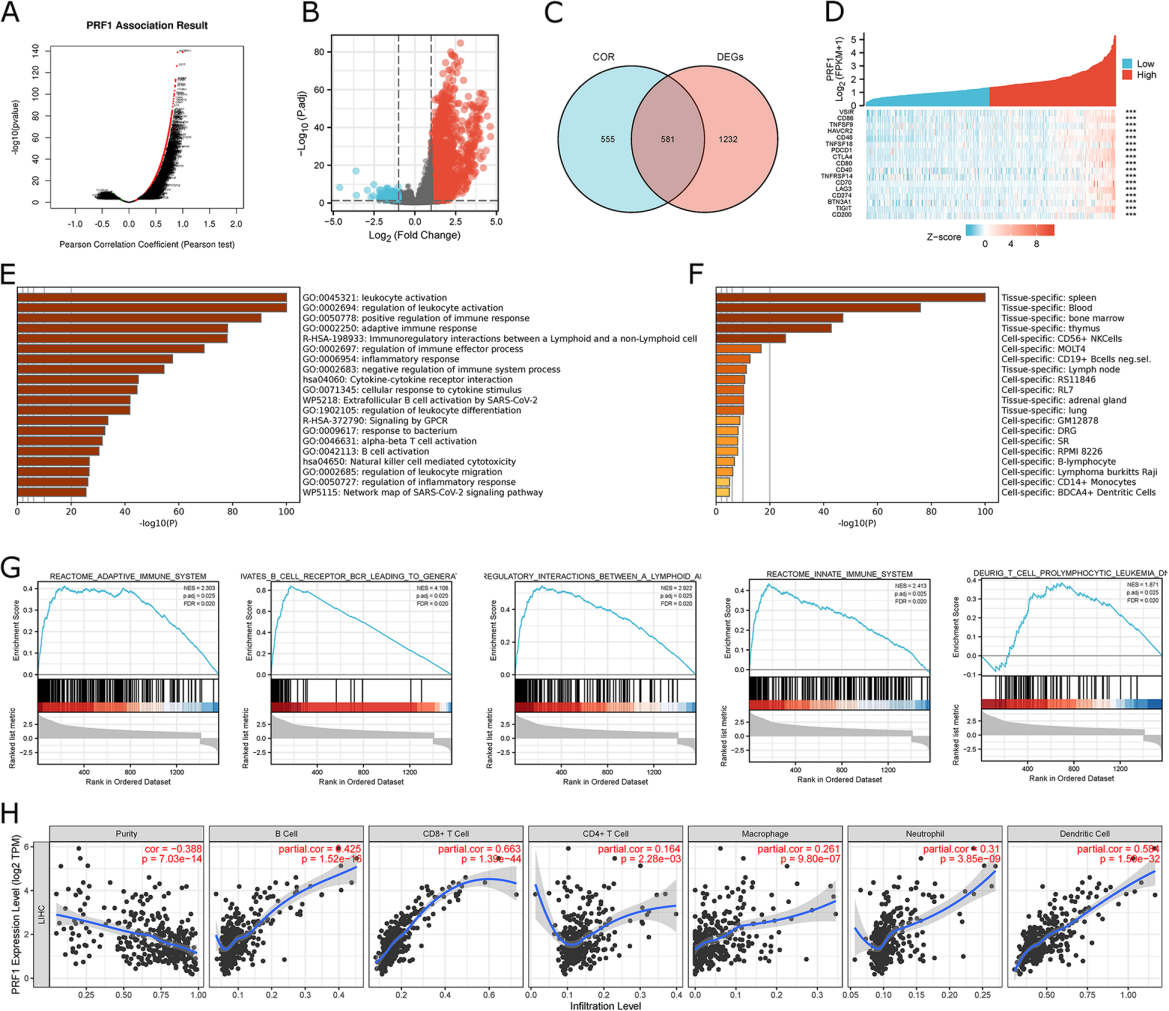
Genes associated with PRF1 and Functional enrichment analysis in TCGA**. A** Volcano plot for the co-expressed genes related to PRF1, analyzed by LinkedOmics. **B** Volcano plot for DEGs between PRF1 high and PRF1 low groups. **C** Venn diagram for the overlapping genes between co-expressed genes and DEGs. **D** Co-expression of PRF1 and immune-related genes. **E** GO and KEGG enrichment analysis of the overlapping genes by Metascape. **F** Tissues and cells enrichment analysis of the overlapping genes by Metascape. **G** GSEA analyses of the overlapping genes. **H** The correlation of PRF1 and immune cells in HCC was analyzed by TIMER.

We found that PRF1 (Perforin 1) was significantly differentially expressed in most cancer types (Fig. S 9A), and PRF1 was highly accurate as a single factor in predicting tumor prognosis in various cancers in the TCGA cohort (Fig. S 9B). Therefore, we further investigated the characterization of PRF1 in the development of HCC. First, genes co-expressed with PRF1 in the TCGA-HCC cohort were investigated in the LinkedOmics database (Fig. 11A). The results revealed 1136 co-expressed genes that were significantly related to PRF1 (|COR|≥0.3 and *P* < 0.05, Supplementary Table S 7). Among these 1136 genes, 1135 were positively associated with PRF1 expression, and 1 was negatively associated with PRF1 expression. The DEGs of the PRF1 high and PRF1 low expression subpopulations were then calculated. 1813 DEGs were identified (|log2 FC| ≥ 1 and *P* < 0.05) as shown in Supplementary Table S 8. Among them, 1600 upregulated and 213 downregulated genes were identified in the PRF1 high-expression group (Fig. 11B). The 1813 DEGs were intersected with 1136 co-expressed genes to identify 581 overlapping genes for further functional analysis (Fig. 11C, Supplementary Table S 9). Figure 11D shows the co-expression relationship of PRF1 with immune-related genes, including VSIR, CD86, LGALS9, and CD200.etc. To study the biological features of the 581 overlapping genes, based on the Metascape database, enrichment analyses for GO and KEGG were performed. Figure 11E lists the top 20 enrichment analysis results, including the adaptive immune system, innate immune system, immunoregulatory interactions, and activates B cell receptors, and the data suggest that PRF1 is an important functional mediator of immune regulation. On the other hand, as shown in Fig. 11F, overlapping genes were enriched in the spleen, blood, and bone marrow (CD56 + NK cells, MOLT4, CD14 + monocytes, and BDCA4 + dendritic cells), additional evidence for an immunomodulatory role of PRF1 in the pathogenesis of HCC is provided. In the TCGA-HCC cohort, molecular pathways significantly altered by PRF1 high expression versus PRF1 low expression were analyzed by GSEA soft tools. The results showed that PRF1 mainly regulates immune-related processes or pathways, including the adaptive and innate immune systems. Antigen activates B cell receptor (BCR), leading to T cell prolymphocytic leukemia (T-PLL) and so on (Fig. 11G). This further suggests an immunomodulatory function of PRF1 in the carcinogenesis of HCC. The TIMER database showed a significant co-expression of PRF1 with immune cells. In particular, CD8 T cells, B cells, and DC cells. (Fig. 11H).

The ICBatlas database was used to compare pre-treatment and post-treatment differences in PRF1 expression in several immunotherapy cohorts (Fig. S 10A). PRF1 expression differences between responders and non-responders were compared across various immunotherapy cohorts (Fig. S 10B). The results showed that PRF1 levels were elevated after treatment or in the response group, suggesting that PRF1 may play an essential role in tumor immunotherapy.

**Fig. 12:**
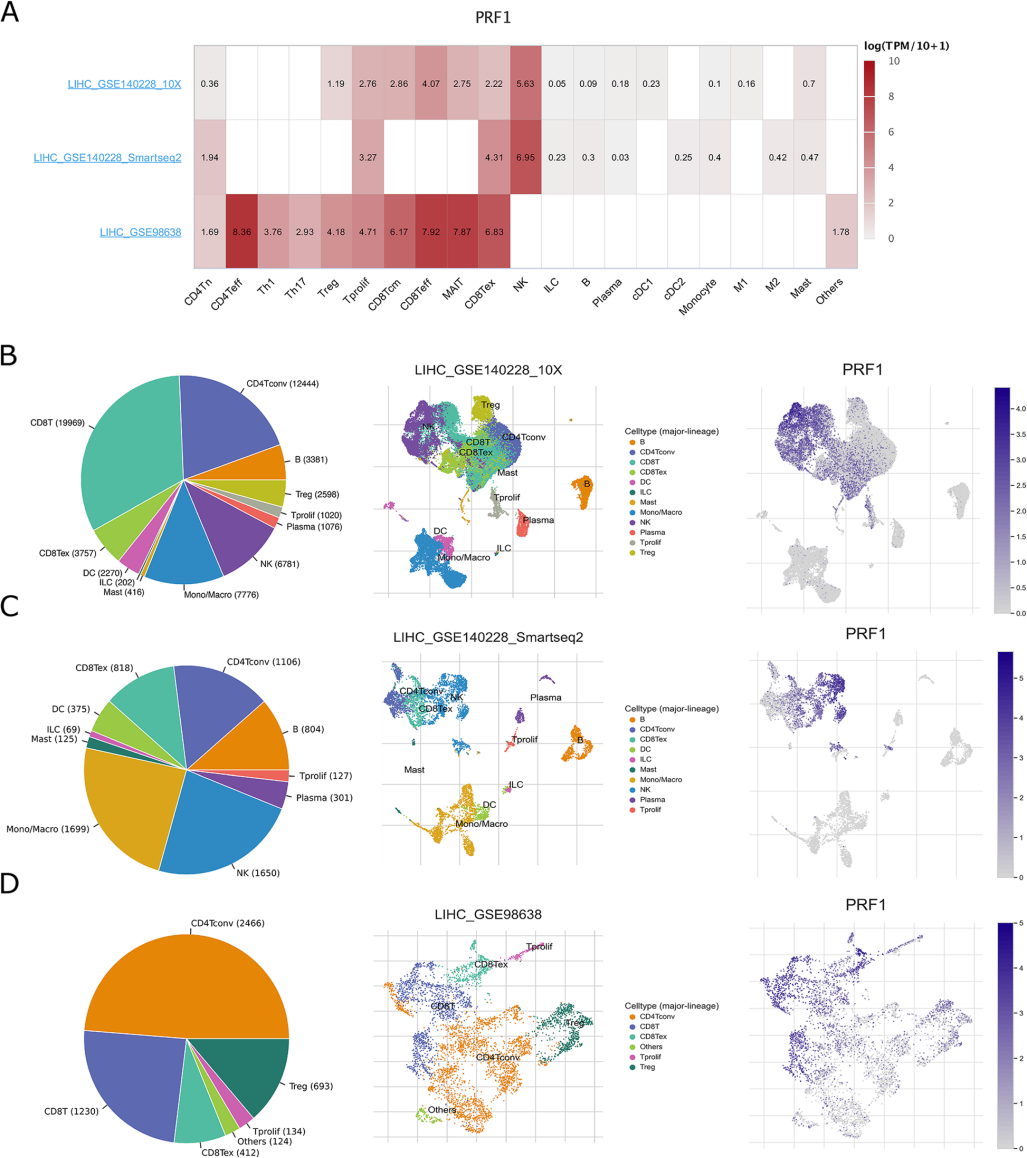
Single-cell annotation of PRF1 in HCC by TISCH.** A** Correlation of PRF1 with cell subpopulations. **B** The cell types and their distribution in the HCC_GSE140228_10X dataset and the distribution of PRF1. **C** The cell types and their distribution in the HCC_GSE140228_Smartseq2 dataset, and the distribution of PRF1. **D** The cell types and their distribution in the HCC_GSE98638 dataset and the distribution of PRF1.

Single-cell RNA sequencing provides greater insight into cellular behavior in complex tumor microenvironments [49]. We further investigated the expression of PRF1 at the single-cell level. The distribution of PRF1 expression in different datasets is shown using the TISCH database (Fig. 12A). PRF1 was expressed in CD4Tn, Treg, Tprolif, CD8Tcm, CD8Teff, MAIT, CD8Tex, and NK cell types in HCC_GSE140228_10X (Fig. 12B). PRF1 was expressed in CD4Tn, Tprolif, CD8Tex, and NK cell types in HCC_GSE140228_Smartseq2 (Fig. 12C). PRF1 was expressed in CD4Tn, CD4Teff, Th1, Th17, Treg, Tprolif, CD8Tcm, CD8Teff, MAIT, CD8Tex and NK cell types in HCC_GSE98638 (Fig. 12D). The results indicate that the functional annotation of PRF1 at the single-cell level is associated with T cells and NK cells. Cell trajectory analysis is used to reconstruct the process of cellular change over time by constructing intercellular change trajectories. Cell trajectory analysis allows the verification of known cellular differentiation relationships at single-cell resolution and the mining of cellular subpopulations and marker genes critical for differentiation [50]. The expression distribution of PRF1 in different cell types in HCC_GSE125449_10x was explored with TIGER (Fig. S 11A), and the results indicate that PRF1 is primarily expressed in NK/NKT cell types (Fig. S 11B-C) and was a marker gene of NK/NKT cells. It is suggested that PRF1 expression is associated with NK/NKT cell immune infiltration. Here, we reiterated the importance of immune infiltration of T cells and NK cells in immunogenic cell death [51] and that PRF1 might be an essential marker of immune cell infiltration of T cells and NK cells.

## Discussion

Immunogenic cell death has been identified as a functionally unique form of stress-mediated cell death (RCD) [52], and a growing number of studies strongly emphasize the ability of ICDs to induce specific anticancer immune responses [53]. ICDs trigger a complete adaptive immune response by releasing danger signals to reshape the tumor immune microenvironment, which would contribute to immunotherapy [54]. The combination of immunogenic therapy and new immunotherapy regimens holds great promise for treating malignancies, and cancer biology is focusing more and more on this area [55–58]. Among the clinically known ICD inducers, only a few drugs, including adriamycin and anthracyclines, epirubicin, idarubicin, mitoxantrone, bleomycin, Vanco bortezomib, and oxaliplatin, have been shown to induce true ICD. However, these inducers remain suboptimal due to cytotoxicity. It is crucial to find a drug that can directly cause ICD in tumors [59]. The effects of ICDs on the tumor immune microenvironment, invasive tumor migration, and their prognostic role in HCC are poorly understood.

In our study, we comprehensively understood the association between the differential and clinical characteristics of ICDs expression in HCC. Two ICD clusters were identified by consensus clustering, and ICDs were relatively highly expressed in the ICD-high clusters and relatively low expressed in the ICD-low clusters. Analyzing and evaluating both groups, we found that patients in the ICD-high clusters had significantly longer OS, considerably higher levels of immune infiltration, significantly higher immune function scores, and significantly higher expression levels of immune activator-related genes. Therefore, we defined ICD-high as the immune-activated hot tumor phenotype and ICD-low as the immune-suppressed cold tumor phenotype. As the tumor becomes immunogenic, its immune microenvironment changes, which mediates the body’s production of an antitumor immune response when tumor cells undergo immunogenic death by external stimulation [60]. We hypothesize that this change is related to the expression of ICDs. We then constructed ICDRM, including 5-ICD genes using the Cox-Lasso algorithm. ICDRM was used to classify patients into high-risk and low-risk subpopulations. ICDRM showed a high predictive value for OS and could be used as an independent prognostic factor for patients with HCC. We validated the validity and stability of ICDRM to predict prognosis in datasets of three different platforms (TCGA, ICGC, and GEO).

Additionally, we compared the infiltration of immune cells and immune-related gene expression among the two subpopulations. We found that ICDRM could discriminate ICD clusters and thus further assess TME differences in HCC patients, distinguishing whether the immune phenotype was immunosuppressed cold tumors or immune-activated hot tumors. Use ICDRM to predict tumor immunotherapy’s efficacy and identify patients more likely to benefit from immunotherapy. Early diagnosis and treatment have always been the optimal choice in the fight against cancer [61]. The development of circulating tumor DNA (ctDNA) technology has helped identify therapeutic targets and combination treatment strategies [62]. Calculating the patient’s ICDRM risk score can provide valuable information for real-world cancer treatment decisions and realize the clinical value of predictive models [63].

ICDs interact and influence each other; specifically, in the ICDRM we constructed, the downregulation of ATG5, CASP8, and HMGB1 positively influenced patient survival, while the opposite was true for CD4 and PRF1. There is increasing evidence that ICDs is strongly associated with the tumor immune microenvironment and aggressive migration. Studies suggest that HMGB1 is closely associated with tumor proliferation [64]. When cells develop ICD, they release HMGB1 extracellularly. Cancer eradication requires HMGB1 to bind to Toll-like receptor 4, activating immune cells through signaling pathways. Blockade of HMGB1 binding to TLR4 is associated with early recurrence in breast cancer patients [65]. Bax is a crucial regulator of cell death and plays a pivotal role in mitochondrial dysfunction [66]. Its activation leads to mitochondrial membrane permeabilization, releasing cytochrome c and, ultimately, cancer cell death. Surprisingly, our study revealed that the knockdown of Bax inhibited hepatocellular carcinoma cells’ proliferation and metastatic capacity. Studies have demonstrated that aggressive diseases are linked to pro-apoptotic Bax expression or caspase activation, whereas a less aggressive state is associated with anti-apoptotic Bcl-2 [67, 68]. The process of cell death can potentially promote genomic instability and create ecological niches that may lead to the repopulation of more aggressive tumor cell clones in the context of neoplastic and progressive tumors [69, 70]. HSP90AA1, an oncoprotein, regulates protein conformation, stability, and degradation. It is highly expressed in various tumor tissues, including hepatocellular carcinoma, lung cancer, gastric cancer, breast cancer, and esophageal cancer. It is significantly associated with tumor infiltration depth, lymph node metastasis, staging, and grading [71–74]. In hepatocellular carcinoma, HSP90AA1 promotes the growth of HCC cells by enhancing glycolysis and proliferation and reducing apoptosis through pyruvate kinase M2 (PKM2) [40].Studying the characteristics of ICDs in HCC will improve our knowledge of tumor aggressiveness and help develop more individualized and accurate immunotherapy protocols.

Immunotherapy appears beneficial for patients with HCC based on a large body of evidence; however, a poor understanding of the TME and immune infiltration in HCC leads to variable outcomes with the same immunotherapy in patients, which may be related to immune escape [75]. Therefore, a biomarker is urgently needed to predict patients’ responses to immunotherapy. Our study shows that the characterization of ICDRM in the TCGA-HCC cohort can provide stratification of the patient’s tumor microenvironment, distinguishing between cold and hot tumors, and has significant potential for cancer vaccine development [76]. Although ICDs have been used in some preclinical models, there is currently insufficient evidence that ICDRM can be used in clinical practice [77]. In our study, ICDRM has been demonstrated on three different platform datasets, and a consistent prognosis can be predicted for patients with HCC with ICDRM. When we examined treatment outcomes by ICDRM risk score, we found an association between drug sensitivity and ICDRM risk score. Results from clinical trials showed that sorafenib, a drug-sensitive to the high-risk subpopulations, prolonged survival by 2.8 months compared to placebo groups in advanced HCC [78]; and axitinib, a drug-sensitive to the low-risk subpopulations, allowed patients with advanced HCC to survive up to 20.1 months [79], consistent with results predicted by the TCIA database, low-risk subpopulations had higher IPS. There is a possibility that immunotherapy will work better for them and that it will have better results.

In addition, drugs that are more sensitive to low-risk subpopulations of ICDRM may be more beneficial for survival. In addition, based on ICDRM we constructed, patients may benefit more from immunotherapy by changing the immune phenotype of tumor patients so that cold tumors become hot tumors. Whether the three drugs (Camptothecin, Vorinostat, and Etoposide) predicted to be effective for treating HCC based on ICDRM can be used to develop better ICD inducers needs further investigation.

Immunogenic cell death is closely associated with antitumor immune activity [80]. Our further exploration of the critical gene of ICDRM, PRF1, revealed that it could serve as a stable prognostic marker for many tumors and is significantly differentially expressed across cancer types. Single-cell level annotation revealed that PRF1 is expressed in multiple immune cell types, particularly NK and T cells. In addition, PRF1 is a signature gene of NK/NKT cells and a marker for various immune cells. PRF1 has been shown to play a role in allowing tumor cells to perforate and rupture the membrane, releasing more DAMPs and leading to more immune cells recognizing and engulfing tumor cells, an essential feature in activating complete adaptive immunity [81]. Cytotoxic T lymphocytes (CTL) are capable of killing infected cells as well as tumor cells. CTL cytosolic spit out enzymes soluble granzyme, and these granules contain perforin (pore-forming protein) and granzyme (granzyme) and enter the cytotoxic immune synapse between CTL and target cells. Among them, PRF1 can punch holes in the cytosolic membrane of target cells to expose more antigens, allowing granzyme to enter the cytoplasm of target cells rapidly. At the same time, effector cytotoxic T cells can accumulate toward infected cells or tumor sites under the action of chemokines. Thus, cytotoxic T cells secrete effector molecules at high local concentrations, selectively killing target cells without harming adjacent normal cells. These effector molecules enter the cytoplasm and cause target cell apoptosis by activating apoptosis-associated apoptosis enzyme systems [82]. PRF1 plays a perforation-breaking role and may be essential in inducing more immune cells to exert joint antitumor immunity. Therefore, the mechanism of PRF1, an important gene of ICDRM, in the antitumor immune infiltration of NK cells and T cells deserves in-depth investigation.

However, because our analysis was derived from limited data analysis, our study has obvious limitations, namely, the need for large-scale prospective studies and functional or mechanistic experiments to explain the mechanism of ICDs in HCC.

## Conclusion

This study systematically evaluated ICDs, then constructed ICDRM including 5-ICDs, which could be used as a stable and accurate prognostic biomarker of HCC. Our study confirms that the properties of ICDs provide a theoretical basis for their use in developing better ICD inducers with significant potential for cancer vaccine development. ICDRM could be applied to characterize the TME and immune infiltration in patients with HCC, as well as provide new ideas and approaches for developing better and more personalized immunotherapy protocols by determining the immune phenotype of the tumor.

## Supplementary Information

**Additional file 1: Figure S1.** A. Frequenciesof CNV gain, loss, and non-CNV among ICDs in ICD-low clusters. B. Frequenciesof CNV gain, loss, and non-CNV among ICDs in ICD-high clusters. **Figure S2.** A. mRNAlevels of BAX in THLE-2 and HCC cells. B. mRNA levels of BAX in HepG2 and Huh7HCC cells after BAX was knocked down. C-D. A colony formation assay was used toexplore the function of BAX in HCC cells. Their representative images are shownin C. E-F. Knockdown of BAX inhibits HCC cell migration. Wound healing assayswere used to assess the migration of HepG2 and Huh7 cells after the BAXknockdown. Representative images are shown in E (* *P*<0.05, ** *P*<0.01, ***P<0.001).All experiments were repeated at least three times. **Figure S3.** A. Differencesin the expression of RNA modification genes between ICD-low and ICD-highclusters. B. Differences in the expression of chemokine genes between ICD-lowand ICD-high clusters. C. Differences in the expression of receptor genesbetween ICD-low and ICD-high clusters. D. Differences in the expression of HLAgenes between ICD-low and ICD-high clusters. **Figure S4.** A. Frequencies of CNVgain, loss, and non-CNV among ICDs in Risk-high clusters. B. Frequencies of CNVgain, loss, and non-CNV among ICDs in Risk-low clusters. **Figure S5.** A.Prognostic differences according to high or low TMB scores in TCGA. B.Comparison of ICDRM and TMB in predicting prognosis. C. Heatmap of immuneinfiltration differences between ICDRM subpopulations and ICD clusters in TCGA.**Figure S6.** A. Differences in the expression of RNA modification genes between ICDRMRsk-low and Risk-high subpopulations. B. Differences in the expression ofchemokine genes between ICDRM Risk-low and Risk-high subpopulations. C.Differences in the expression of receptor genes between ICDRM Risk-low and Risk-highsubpopulations. D. Differences in the expression of HLA genes between ICDRM Risk-lowand Risk-high subpopulations. **Figure S7.** Analysis of drug sensitivity between ICDRMRisk-low and Risk-high subpopulations about clinically used Chemotherapeuticand targeted drugs through R package "pRRophetic". **Figure S8.** A. Thecorrelation of ICDRM with immune cells. B. Scatter plots show the correlationof ICDRM with the infiltration of activated CD4+ T memory, CD8+ T, T follicularhelper, and gd T cells. **Figure S9.** A. Expression differences of PRF1 between normaland tumor samples in TCGA. B. ROC curves of PRF1 in predicting the diagnosticvalue in TCGA. **Figure S10.** A. Expression differences of PRF1 betweenPre-treatment and On-treatment samples in each immunotherapy dataset. B. Expressiondifferences of PRF1 between Response and Non-response based on Pre-treatmentsamples in all immunotherapy datasets. **Figure S11.** A. The cell types and theirdistribution in HCC GSE125449 10x dataset. B. Relationship between PRF1expression and immune cells in HCC GSE125449 10x dataset. C. The distributionof PRF1 in NK/NKT cells was analyzed using single-cell resolution. **SupplementaryTable S1.** The sequences of qRT-PCR primers and shRNA sequences of BAX and HSP90AA1.**Supplementary Table S2.** DEGs between ICD-low and ICD-high clusters. **SupplementaryTable S3.** GSEA enrichment analysis of the DEGs between ICD-low and ICD-highclusters. **Supplementary Table S4.** DEGs between high-risk and low-risksubpopulations. **Supplementary Table S5.** GSEA enrichment analysis of the DEGs betweenhigh-risk and low-risk subpopulations. **Supplementary Table S6.** The intersectionof the drugs was predicted using the R package "pRRophetic" and the cMAPwebsite. **Supplementary Table S7.** 1136 Co-expressed genes that weresignificantly related to PRF1. **Supplementary Table S8.** 1813 DEGs of the PRF1high and PRF1 low expression subpopulations. **Supplementary Table S9.** 581 overlappinggenes between 1813 DEGs and 1136 co-expressed genes.

## Data Availability

The TCGA data analyzed in the current study are available from the NCI Genomic Data Commons Data Portal (https://portal.gdc.cancer.gov/). The ICGC data are available from the ICGC Data Portal (https://dcc.icgc.org/projects/LIRI-JP). The NCBI GEO data are available from the NCBI Data Portal (https://www.ncbi.nlm.nih.gov/geo/query/acc.cgi?acc=GSE76427).

## Abbreviations

BAX: BCL2 Associated X, Apoptosis Regulator
CCK-8: Cell Counting Kit-8
CTL: Cytotoxic T lymphocytes
DAMPs: Damage-associated molecular patterns
GEO: Gene Expression Omnibus
GO: Genetics Ontology
GSEA: Gene set enrichment analyses
GSVA: Gene set variance analysis
HCC: Hepatocellular carcinoma
HMGB1: High Mobility Histone 1
HSP90AA1: Heat Shock Protein 90 Alpha Family Class A Member 1
ICD: Immunogenic cell death
ICDRM: ICD-related risk model
ICGC: International Cancer Genome Consortium
KEGG: Kyoto Encyclopedia of Genetics and Genomes
MSigDB: Molecular Signature Database
OS: Overall survival
PKM2: Pyruvate Kinase M2
PRF1: Perforin 1
RCD: Regulated cell death
ssGSEA: single sample GSEA
TCGA: The Cancer Genome Atlas
TME: Tumour microenvironment

## References

[1] Vogel A, Meyer T, Sapisochin G, Salem R, Saborowski A (2022). Hepatocellular carcinoma. Lancet.

[2] Zheng R, Qu C, Zhang S, Zeng H, Sun K, Gu X (2018). Liver cancer incidence and mortality in China: temporal trends and projections to 2030. Chin J Cancer Res.

[3] Bruix J, Sherman M (2005). Management of hepatocellular carcinoma. Hepatology (Baltimore MD).

[4] Chen Y-L, Chang M-C, Cheng W-F (2017). Metronomic chemotherapy and immunotherapy in cancer treatment. Cancer Lett.

[5] Krysko DV, Garg AD, Kaczmarek A, Krysko O, Agostinis P, Vandenabeele P (2012). Immunogenic cell death and DAMPs in cancer therapy. Nat Rev Cancer.

[6] Garg AD, Nowis D, Golab J, Vandenabeele P, Krysko DV, Agostinis P (2010). Immunogenic cell death, DAMPs and anticancer therapeutics: an emerging amalgamation. Biochim Biophys Acta.

[7] Oura K, Morishita A, Tani J, Masaki T. Tumor Immune Microenvironment and Immunosuppressive Therapy in Hepatocellular Carcinoma: A Review. International Journal of Molecular Sciences. 2021;22(11):5801. 10.3390/ijms22115801 PMC8198390 34071550

[8] Garg AD, De Ruysscher D, Agostinis P (2016). Immunological metagene signatures derived from immunogenic cancer cell death associate with improved survival of patients with lung, breast or ovarian malignancies: a large-scale meta-analysis. Oncoimmunology.

[9] Hudson TJ, Anderson W, Artez A, Barker AD, Bell C, Bernabé RR (2010). International network of cancer genome projects. Nature.

[10] Liu Y, Wang J, Li L, Qin H, Wei Y, Zhang X (2022). AC010973.2 promotes cell proliferation and is one of six stemness-related genes that predict overall survival of renal clear cell carcinoma. Sci Rep.

[11] Wilkerson MD, Hayes DN (2010). ConsensusClusterPlus: a class discovery tool with confidence assessments and item tracking. Bioinf (Oxford England).

[12] Jiang S, Ren X, Liu S, Lu Z, Xu A, Qin C, Wang Z (2021). Integrated Analysis of the Prognosis-Associated RNA-Binding protein genes and candidate drugs in renal papillary cell carcinoma. Front Genet.

[13] Wu D, Yin Z, Ji Y, Li L, Li Y, Meng F (2021). Identification of novel autophagy-related lncRNAs associated with a poor prognosis of colon adenocarcinoma through bioinformatics analysis. Sci Rep.

[14] Yu L, Shen H, Ren X, Wang A, Zhu S, Zheng Y, Wang X (2021). Multi-omics analysis reveals the interaction between the complement system and the coagulation cascade in the development of endometriosis. Sci Rep.

[15] Goel MK, Khanna P, Kishore J (2010). Understanding survival analysis: Kaplan-Meier estimate. Int J Ayurveda Res.

[16] Subramanian A, Tamayo P, Mootha VK, Mukherjee S, Ebert BL, Gillette MA (2005). Gene set enrichment analysis: a knowledge-based approach for interpreting genome-wide expression profiles. Proc Natl Acad Sci USA.

[17] Hänzelmann S, Castelo R, Guinney J (2013). GSVA: gene set variation analysis for microarray and RNA-seq data. BMC Bioinformatics.

[18] Chen B, Khodadoust MS, Liu CL, Newman AM, Alizadeh AA (2018). Profiling Tumor infiltrating Immune cells with CIBERSORT. Methods In Molecular Biology (Clifton NJ).

[19] Hu F-F, Liu C-J, Liu L-L, Zhang Q, Guo A-Y. Expression profile of immune checkpoint genes and their roles in predicting immunotherapy response. Brief Bioinform. 2021;22(3):bbaa176. 10.1093/bib/bbaa176 32814346

[20] Mayakonda A, Lin D-C, Assenov Y, Plass C, Koeffler HP (2018). Maftools: efficient and comprehensive analysis of somatic variants in cancer. Genome Res.

[21] Gu Z, Eils R, Schlesner M (2016). Complex heatmaps reveal patterns and correlations in multidimensional genomic data. Bioinf (Oxford England).

[22] Park SY, Nomogram (2018). An analogue tool to deliver digital knowledge. J Thorac Cardiovasc Surg.

[23] Zanfardino M, Pane K, Mirabelli P, Salvatore M, Franzese M. TCGA-TCIA Impact on Radiogenomics Cancer Research: A Systematic Review. International Journal of Molecular Sciences. 2019;20(23):6033. 10.3390/ijms20236033 PMC6929079 31795520

[24] Hugo W, Zaretsky JM, Sun L, Song C, Moreno BH, Hu-Lieskovan S (2016). Genomic and transcriptomic features of response to Anti-PD-1 therapy in metastatic melanoma. Cell.

[25] Van Allen EM, Miao D, Schilling B, Shukla SA, Blank C, Zimmer L (2015). Genomic correlates of response to CTLA-4 blockade in metastatic melanoma. Science.

[26] Geeleher P, Cox N, Huang RS (2014). pRRophetic: an R package for prediction of clinical chemotherapeutic response from tumor gene expression levels. PLoS ONE.

[27] Hafner M, Niepel M, Chung M, Sorger PK (2016). Growth rate inhibition metrics correct for confounders in measuring sensitivity to cancer drugs. Nat Methods.

[28] Yang K, Dinasarapu AR, Reis ES, Deangelis RA, Ricklin D, Subramaniam S, Lambris JD (2013). CMAP: complement map database. Bioinf (Oxford England).

[29] Kim S, Chen J, Cheng T, Gindulyte A, He J, He S (2021). PubChem in 2021: new data content and improved web interfaces. Nucleic Acids Res.

[30] Shen W, Song Z, Zhong X, Huang M, Shen D, Gao P (2022). Sangerbox: a comprehensive, interaction-friendly clinical bioinformatics analysis platform. iMeta.

[31] Vasaikar SV, Straub P, Wang J, Zhang B (2018). LinkedOmics: analyzing multi-omics data within and across 32 cancer types. Nucleic Acids Res.

[32] Zhou Y, Zhou B, Pache L, Chang M, Khodabakhshi AH, Tanaseichuk O (2019). Metascape provides a biologist-oriented resource for the analysis of systems-level datasets. Nat Commun.

[33] Li T, Fan J, Wang B, Traugh N, Chen Q, Liu JS (2017). TIMER: a web server for Comprehensive Analysis of Tumor-Infiltrating Immune cells. Cancer Res.

[34] Sun D, Wang J, Han Y, Dong X, Ge J, Zheng R (2021). TISCH: a comprehensive web resource enabling interactive single-cell transcriptome visualization of tumor microenvironment. Nucleic Acids Res.

[35] Chen Z, Luo Z, Zhang D, Li H, Liu X, Zhu K, et al. TIGER: A Web Portal of Tumor Immunotherapy Gene Expression Resource. Genomics Proteomics Bioinformatics. 2022;S1672-0229(22)00099-7.10.1016/j.gpb.2022.08.00436049666

[36] Yang M, Miao Y-R, Xie G-Y, Luo M, Hu H, Kwok HF (2022). ICBatlas: a Comprehensive Resource for depicting Immune Checkpoint Blockade Therapy characteristics from Transcriptome Profiles. Cancer Immunol Res.

[37] Gentleman RC, Carey VJ, Bates DM, Bolstad B, Dettling M, Dudoit S (2004). Bioconductor: open software development for computational biology and bioinformatics. Genome Biol.

[38] Huber W, Carey VJ, Gentleman R, Anders S, Carlson M, Carvalho BS (2015). Orchestrating high-throughput genomic analysis with Bioconductor. Nat Methods.

[39] Pan Z, Bao Y, Hu M, Zhu Y, Tan C, Fan L (2023). Role of NAT10-mediated ac4C-modified HSP90AA1 RNA acetylation in ER stress-mediated metastasis and lenvatinib resistance in hepatocellular carcinoma. Cell Death Discov.

[40] Xu Q, Tu J, Dou C, Zhang J, Yang L, Liu X (2017). HSP90 promotes cell glycolysis, proliferation and inhibits apoptosis by regulating PKM2 abundance via Thr-328 phosphorylation in hepatocellular carcinoma. Mol Cancer.

[41] Liu L, Deng Y, Zheng Z, Deng Z, Zhang J, Li J (2021). Hsp90 inhibitor STA9090 sensitizes Hepatocellular Carcinoma to Hyperthermia-Induced DNA damage by suppressing DNA-PKcs protein Stability and mRNA transcription. Mol Cancer Ther.

[42] Zhao S, Zhang Y, Lu X, Ding H, Han B, Song X (2021). CDC20 regulates the cell proliferation and radiosensitivity of P53 mutant HCC cells through the Bcl-2/Bax pathway. Int J Biol Sci.

[43] Gai X, Tu K, Li C, Lu Z, Roberts LR, Zheng X (2015). Histone acetyltransferase PCAF accelerates apoptosis by repressing a GLI1/BCL2/BAX axis in hepatocellular carcinoma. Cell Death Dis.

[44] Hall BG, Clarke ND (1977). Regulation of newly evolved enzymes. III evolution of the ebg repressor during selection for enhanced lactase activity. Genetics.

[45] Jardim DL, Goodman A, de Melo Gagliato D, Kurzrock R (2021). The Challenges of Tumor Mutational Burden as an Immunotherapy Biomarker. Cancer Cell.

[46] Newman AM, Liu CL, Green MR, Gentles AJ, Feng W, Xu Y (2015). Robust enumeration of cell subsets from tissue expression profiles. Nat Methods.

[47] Newman AM, Steen CB, Liu CL, Gentles AJ, Chaudhuri AA, Scherer F (2019). Determining cell type abundance and expression from bulk tissues with digital cytometry. Nat Biotechnol.

[48] Charoentong P, Finotello F, Angelova M, Mayer C, Efremova M, Rieder D (2017). Pan-cancer immunogenomic analyses reveal genotype-immunophenotype Relationships and Predictors of response to checkpoint blockade. Cell Rep.

[49] Papalexi E, Satija R (2018). Single-cell RNA sequencing to explore immune cell heterogeneity. Nat Rev Immunol.

[50] Hwang B, Lee JH, Bang D. Single-cell RNA sequencing technologies and bioinformatics pipelines. Exp Mol Med. 2018;50(8):1-14. 10.1038/s12276-018-0071-8 PMC6082860 30089861

[51] Tang R, Xu J, Zhang B, Liu J, Liang C, Hua J (2020). Ferroptosis, necroptosis, and pyroptosis in anticancer immunity. J Hematol Oncol.

[52] Galluzzi L, Buqué A, Kepp O, Zitvogel L, Kroemer G. Immunogenic cell death in cancer and infectious disease. Nat Rev Immunol. 2017;17(2):97-111. 10.1038/nri.2016.107 27748397

[53] Ahmed A, Tait SWG (2020). Targeting immunogenic cell death in cancer. Mol Oncol.

[54] Kroemer G, Galassi C, Zitvogel L, Galluzzi L (2022). Immunogenic cell stress and death. Nat Immunol.

[55] Yang W, Zhang F, Deng H, Lin L, Wang S, Kang F (2020). Smart nanovesicle-mediated immunogenic cell death through Tumor Microenvironment Modulation for effective photodynamic immunotherapy. ACS Nano.

[56] Minnie SA, Hill GR (2020). Immunotherapy of multiple myeloma. J Clin Invest.

[57] Li Y, Zhang H, Li Q, Zou P, Huang X, Wu C, Tan L (2020). CDK12/13 inhibition induces immunogenic cell death and enhances anti-PD-1 anticancer activity in breast cancer. Cancer Lett.

[58] Duan X, Chan C, Lin W (2019). Nanoparticle-mediated immunogenic cell death enables and potentiates Cancer Immunotherapy. Angewandte Chemie (International Ed in English)..

[59] Fucikova J, Kepp O, Kasikova L, Petroni G, Yamazaki T, Liu P (2020). Detection of immunogenic cell death and its relevance for cancer therapy. Cell Death Dis.

[60] Dai E, Zhu Z, Wahed S, Qu Z, Storkus WJ, Guo ZS (2021). Epigenetic modulation of antitumor immunity for improved cancer immunotherapy. Mol Cancer.

[61] Moding EJ, Nabet BY, Alizadeh AA, Diehn M (2021). Detecting liquid remnants of solid tumors: circulating Tumor DNA minimal residual disease. Cancer Discov.

[62] Zhang Y, Yao Y, Xu Y, Li L, Gong Y, Zhang K (2021). Pan-cancer circulating tumor DNA detection in over 10,000 chinese patients. Nat Commun.

[63] Cheng ML, Pectasides E, Hanna GJ, Parsons HA, Choudhury AD, Oxnard GR (2021). Circulating tumor DNA in advanced solid tumors: clinical relevance and future directions. CA Cancer J Clin.

[64] Palumbo R, Sampaolesi M, De Marchis F, Tonlorenzi R, Colombetti S, Mondino A (2004). Extracellular HMGB1, a signal of tissue damage, induces mesoangioblast migration and proliferation. J Cell Biol.

[65] Apetoh L, Ghiringhelli F, Tesniere A, Criollo A, Ortiz C, Lidereau R (2007). The interaction between HMGB1 and TLR4 dictates the outcome of anticancer chemotherapy and radiotherapy. Immunol Rev.

[66] Liu Z, Ding Y, Ye N, Wild C, Chen H, Zhou J (2016). Direct activation of bax protein for Cancer Therapy. Med Res Rev.

[67] Bairey O, Zimra Y, Shaklai M, Okon E, Rabizadeh E (1999). Bcl-X, Bax, and bak expression in short- and long-lived patients with diffuse large B-cell lymphomas. Clin Cancer Res.

[68] Sierra A, Lloveras B, Castellsagué X, Moreno L, García-Ramirez M, Fabra A (1995). Bcl-2 expression is associated with lymph node metastasis in human ductal breast carcinoma. Int J Cancer.

[69] Gavathiotis E, Reyna DE, Bellairs JA, Leshchiner ES, Walensky LD (2012). Direct and selective small-molecule activation of proapoptotic BAX. Nat Chem Biol.

[70] Reyna DE, Garner TP, Lopez A, Kopp F, Choudhary GS, Sridharan A, et al. Direct Activation of BAX by BTSA1 Overcomes Apoptosis Resistance in Acute Myeloid Leukemia. Cancer Cell. 2017;32(4):490-505.e10. 10.1016/j.ccell.2017.09.001 PMC5793879 29017059

[71] Birbo B, Madu EE, Madu CO, Jain A, Lu Y. Role of HSP90 in Cancer. International Journal of Molecular Sciences. 2021;22(19):10317. 10.3390/ijms221910317 PMC8508648 34638658

[72] Trepel J, Mollapour M, Giaccone G, Neckers L (2010). Targeting the dynamic HSP90 complex in cancer. Nat Rev Cancer.

[73] Condelli V, Crispo F, Pietrafesa M, Lettini G, Matassa DS, Esposito F, et al. HSP90 Molecular Chaperones, Metabolic Rewiring, and Epigenetics: Impact on Tumor Progression and Perspective for Anticancer Therapy. Cells. 2019;8(6):532. 10.3390/cells8060532 PMC6627532 31163702

[74] Wang L, Zhang Q, You Q (2022). Targeting the HSP90-CDC37-kinase chaperone cycle: a promising therapeutic strategy for cancer. Med Res Rev.

[75] Yarchoan M, Hopkins A, Jaffee EM (2017). Tumor mutational burden and response rate to PD-1 inhibition. N Engl J Med.

[76] Green DR, Ferguson T, Zitvogel L, Kroemer G (2009). Immunogenic and tolerogenic cell death. Nat Rev Immunol.

[77] Kroemer G, Galluzzi L, Kepp O, Zitvogel L (2013). Immunogenic cell death in cancer therapy. Annu Rev Immunol.

[78] Bruix J, Takayama T, Mazzaferro V, Chau G-Y, Yang J, Kudo M (2015). Adjuvant sorafenib for hepatocellular carcinoma after resection or ablation (STORM): a phase 3, randomised, double-blind, placebo-controlled trial. Lancet Oncol.

[79] Adachi Y, Kamiyama H, Ichikawa K, Fukushima S, Ozawa Y, Yamaguchi S (2022). Inhibition of FGFR reactivates IFNγ Signaling in Tumor cells to enhance the combined antitumor activity of Lenvatinib with Anti-PD-1 antibodies. Cancer Res.

[80] Bindea G, Mlecnik B, Tosolini M, Kirilovsky A, Waldner M, Obenauf AC (2013). Spatiotemporal dynamics of intratumoral immune cells reveal the immune landscape in human cancer. Immunity.

[81] Bassez A, Vos H, Van Dyck L, Floris G, Arijs I, Desmedt C (2021). A single-cell map of intratumoral changes during anti-PD1 treatment of patients with breast cancer. Nat Med.

[82] Bálint Å, Müller S, Fischer R, Kessler BM, Harkiolaki M, Valitutti S, Dustin ML (2020). Supramolecular attack particles are autonomous killing entities released from cytotoxic T cells. Science.

