# Supplementary material for: Analyzing molecular typing and clinical application of immunogenic cell death-related genes in hepatocellular carcinoma
Source: BMC Cancer. 2023 Jun 8;23:522. doi: 10.1186/s12885-023-10992-2 (PMC10249577; doi:10.1186/s12885-023-10992-2)
Supplement: Supplementary file 1 — Additional file 1: Figure S1. A. Frequenciesof CNV gain, loss, and non-CNV among ICDs in ICD-low clusters. B. Frequenciesof CNV gain, loss, and non-CNV among ICDs in ICD-high clusters. Figure S2. A. mRNAlevels of BAX in THLE-2 and HCC cells. B. mRNA levels of BAX in HepG2 and Huh7HCC cells after BAX was knocked down. C-D. A colony formation assay was used toexplore the function of BAX in HCC cells. Their representative images are shownin C. E-F. Knockdown of BAX inhibits HCC cell migration. Wound healing assayswere used to assess the migration of HepG2 and Huh7 cells after the BAXknockdown. Representative images are shown in E (* P<0.05, ** P<0.01, ***P<0.001).All experiments were repeated at least three times. Figure S3. A. Differencesin the expression of RNA modification genes between ICD-low and ICD-highclusters. B. Differences in the expression of chemokine genes between ICD-lowand ICD-high clusters. C. Differences in the expression of receptor genesbetween ICD-low and ICD-high clusters. D. Differences in the expression of HLAgenes between ICD-low and ICD-high clusters. Figure S4. A. Frequencies of CNVgain, loss, and non-CNV among ICDs in Risk-high clusters. B. Frequencies of CNVgain, loss, and non-CNV among ICDs in Risk-low clusters. Figure S5. A.Prognostic differences according to high or low TMB scores in TCGA. B.Comparison of ICDRM and TMB in predicting prognosis. C. Heatmap of immuneinfiltration differences between ICDRM subpopulations and ICD clusters in TCGA.Figure S6. A. Differences in the expression of RNA modification genes between ICDRMRsk-low and Risk-high subpopulations. B. Differences in the expression ofchemokine genes between ICDRM Risk-low and Risk-high subpopulations. C.Differences in the expression of receptor genes between ICDRM Risk-low and Risk-highsubpopulations. D. Differences in the expression of HLA genes between ICDRM Risk-lowand Risk-high subpopulations. Figure S7. Analysis of drug sensitivity between ICDRMRisk-low and Risk-high subpo [file 12885_2023_10992_MOESM1_ESM.zip › Supplementary material.docx]

Supplementary Material

Analyzing molecular typing and clinical application of immunogenic cell death-related genes in hepatocellular carcinoma

Cai-Feng Lin^1†,2^, Zhi-Wen Chen^1†^, Feng-Ping Kang^1†^,Jian-Fei Hu^1^, Long Huang^1^, Cheng-Yu Liao^1^, Jian-Lin Lai^1^ , Yi Huang ^1,3^ , Zu-Wei Wang^1*^, Yi-Feng Tian^1,2*^, Shi Chen^1,2*^

^1^ Shengli Clinical Medical College of Fujian Medical University, Fuzhou 350001, Fujian Province, People's Republic of China.

^2^ Department of Hepatopancreatobiliary Surgery, Fujian Provincial Hospital, Fujian Medical University, No. 134, East Street, Fuzhou 350001, Fujian Province, People's Republic of China.

^3^ Center for Experimental Research in Clinical Medicine, Fujian Provincial Hospital, Fuzhou 350001, Fujian Province, People's Republic of China.

**† These authors contributed equally.**

*** Correspondence:*** Corresponding author: Shi Chen, Professor, Department of Hepatobiliary Surgery, Shengli Clinical Medical College of Fujian Medical University, No. 134, East Street, Fuzhou, 350001, Fujian Province, PR China. Email: wawljwalj@163.com

** Corresponding author: Yi-Feng Tian, Department of Hepatobiliary Surgery， Shengli Clinical Medical College of Fujian Medical University, No. 134, East Street, Fuzhou, 350001, Fujian Province, PR China. Email: yifeng0887@126.com

*** Corresponding author: Zu-Wei Wang, Shengli Clinical Medical College of Fujian Medical University, Fujian Medical University, Fuzhou 350001, China. Email addresses: drzuwei123@163.com

## Supplementary Figures


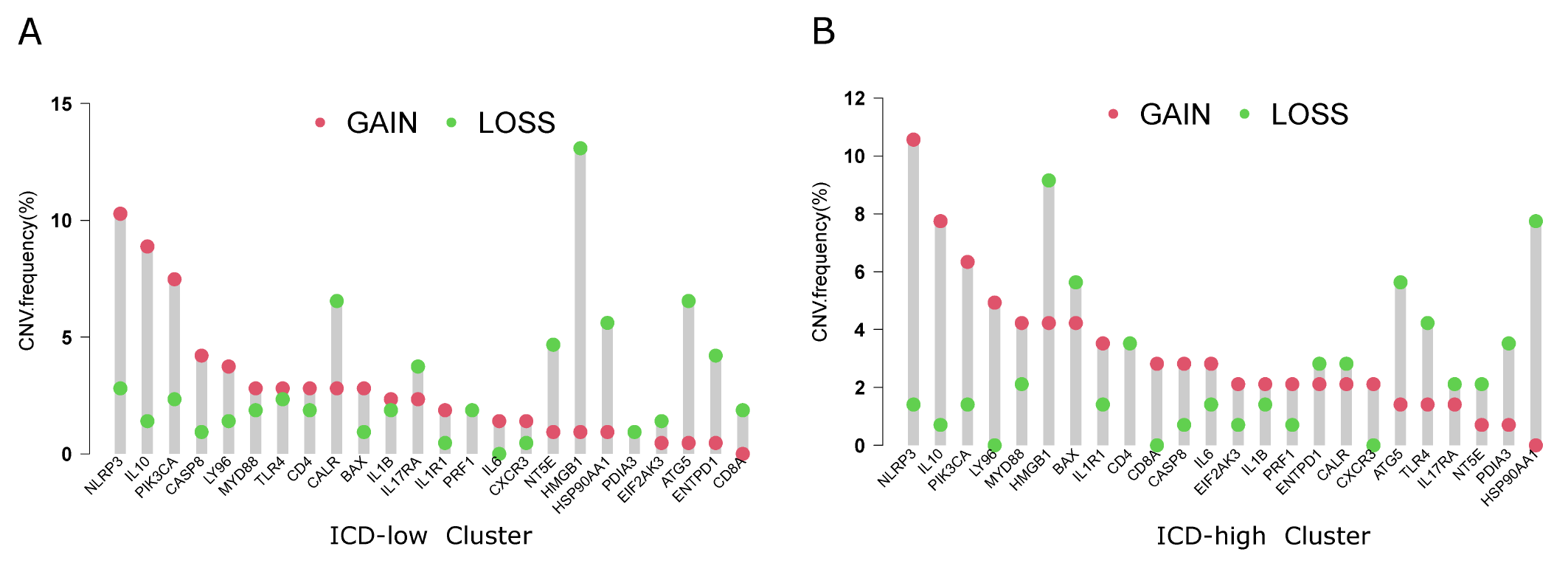
Figure. S1. A. Frequencies of CNV gain, loss, and non-CNV among ICDs in ICD-low clusters. B. Frequencies of CNV gain, loss, and non-CNV among ICDs in ICD-high clusters.


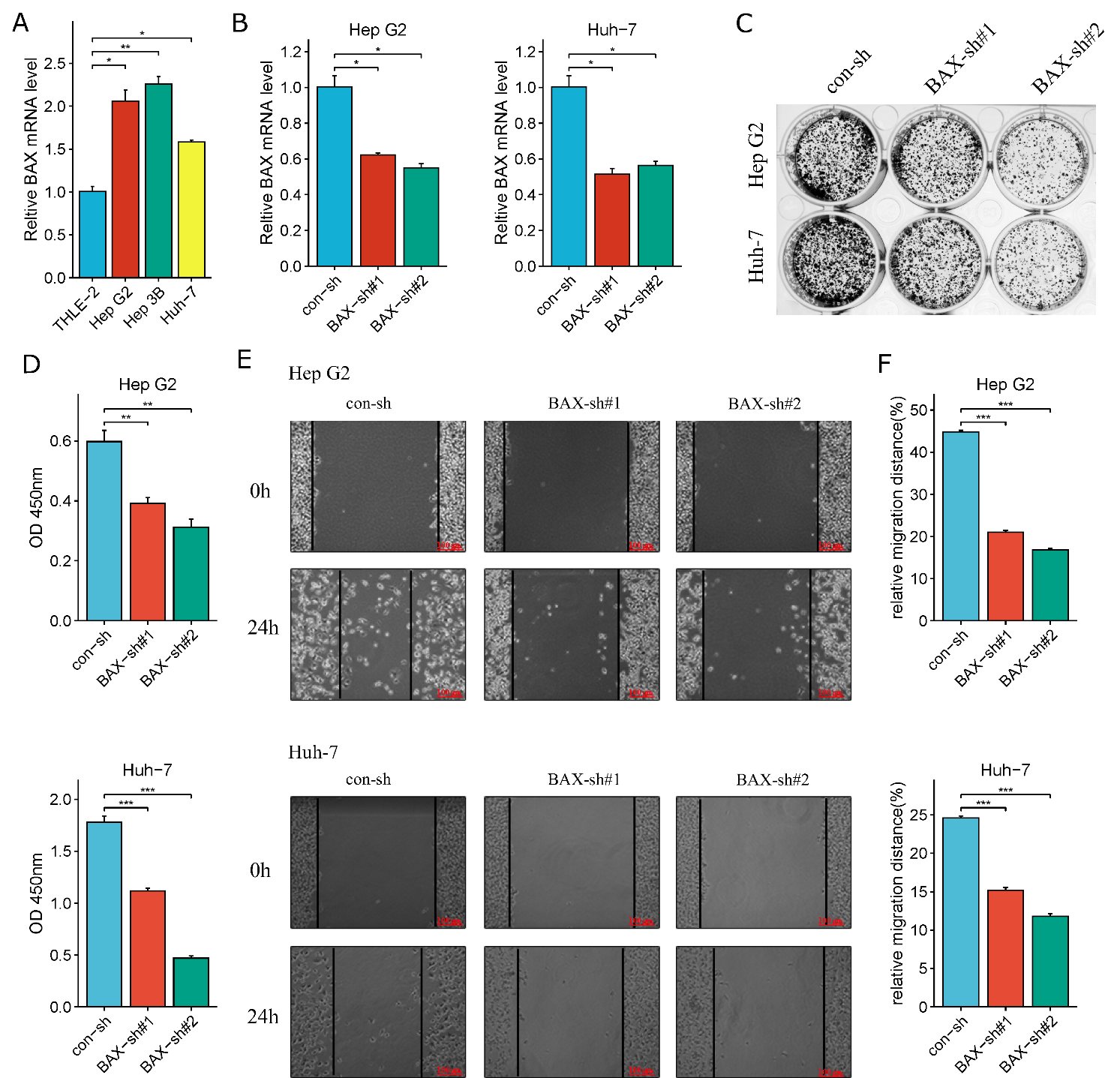


Figure S2. A. mRNA levels of BAX in THLE-2 and HCC cells. B. mRNA levels of BAX in HepG2 and Huh7 HCC cells after BAX was knocked down. C-D. A colony formation assay was used to explore the function of BAX in HCC cells. Their representative images are shown in C. E-F. Knockdown of BAX inhibits HCC cell migration. Wound healing assays were used to assess the migration of HepG2 and Huh7 cells after the BAX knockdown. Representative images are shown in E (* *P*<0.05, ** *P*<0.01, ****P*<0.001). All experiments were repeated at least three times.


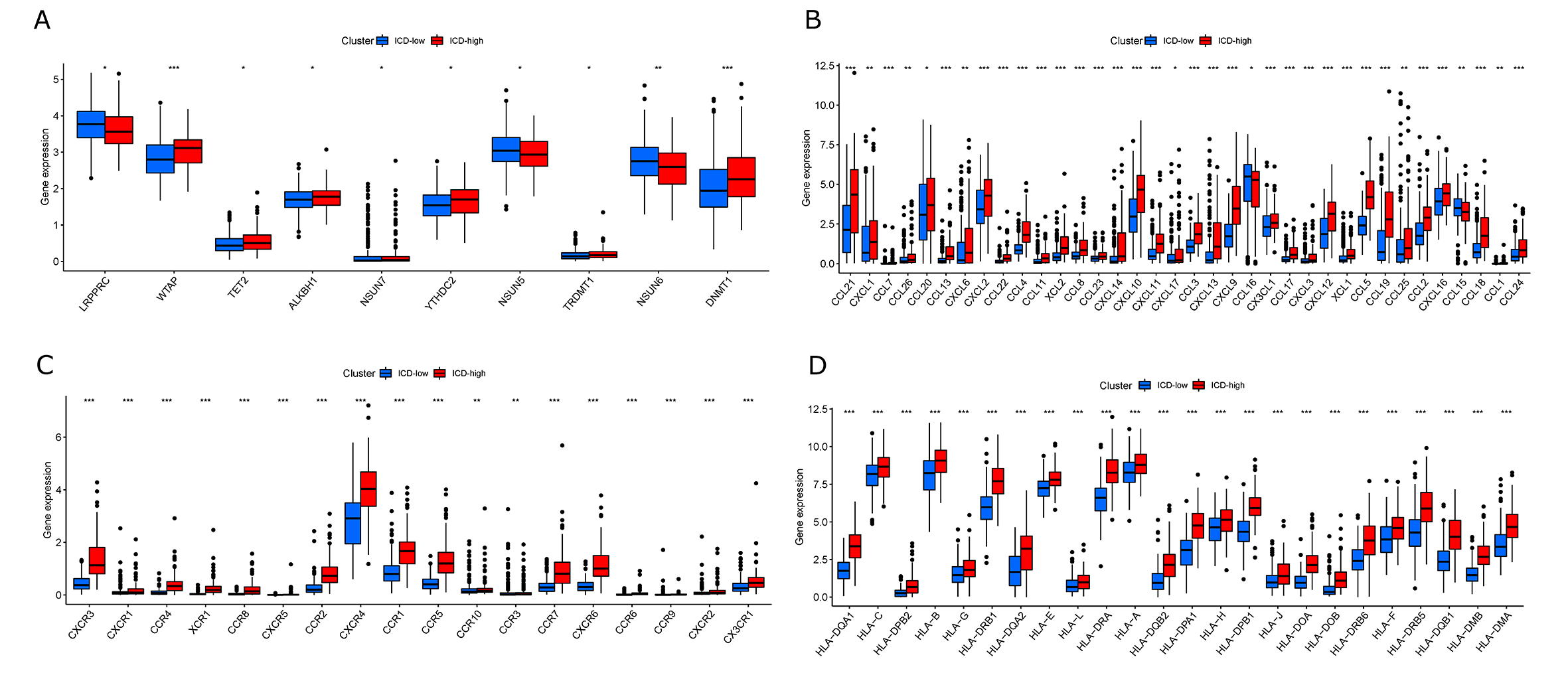


**Figure. S3.** **A.** Differences in the expression of RNA modification genes between ICD-low and ICD-high clusters. **B.** Differences in the expression of chemokine genes between ICD-low and ICD-high clusters. **C.** Differences in the expression of receptor genes between ICD-low and ICD-high clusters. **D.** Differences in the expression of HLA genes between ICD-low and ICD-high clusters.


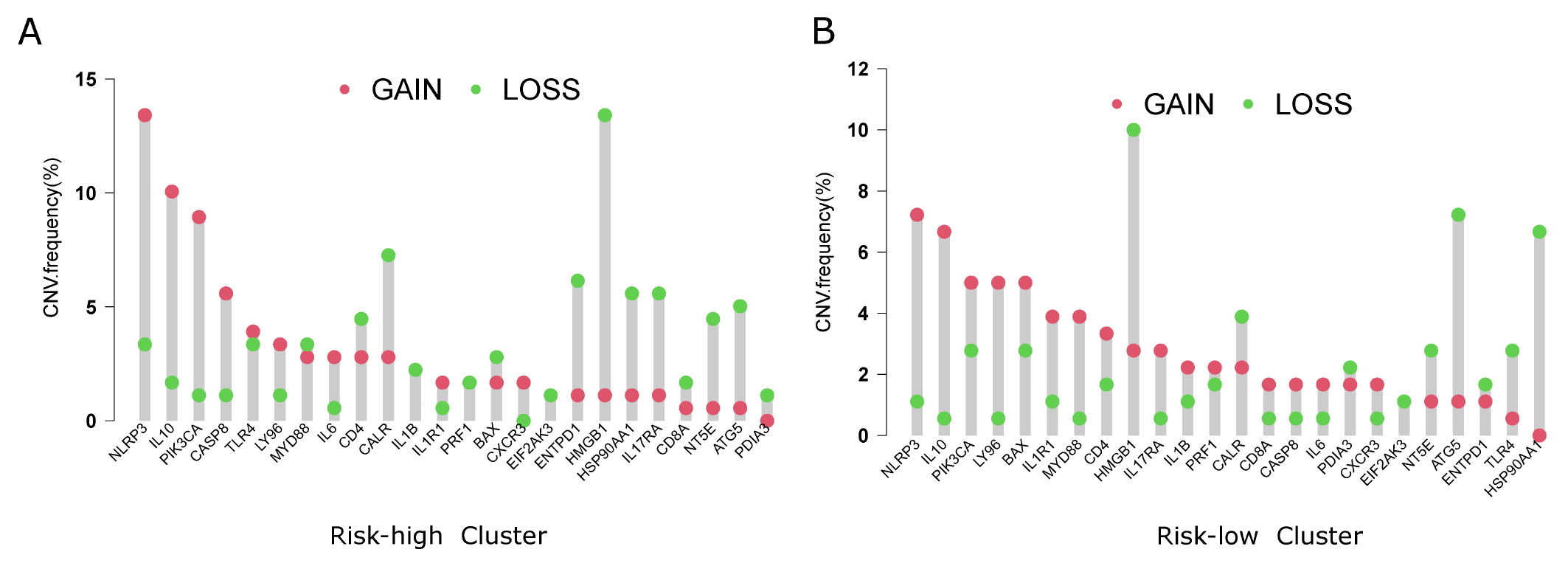
**Figure. S4. A.** Frequencies of CNV gain, loss, and non-CNV among ICDs in Risk-high clusters. **B.** Frequencies of CNV gain, loss, and non-CNV among ICDs in Risk-low clusters.


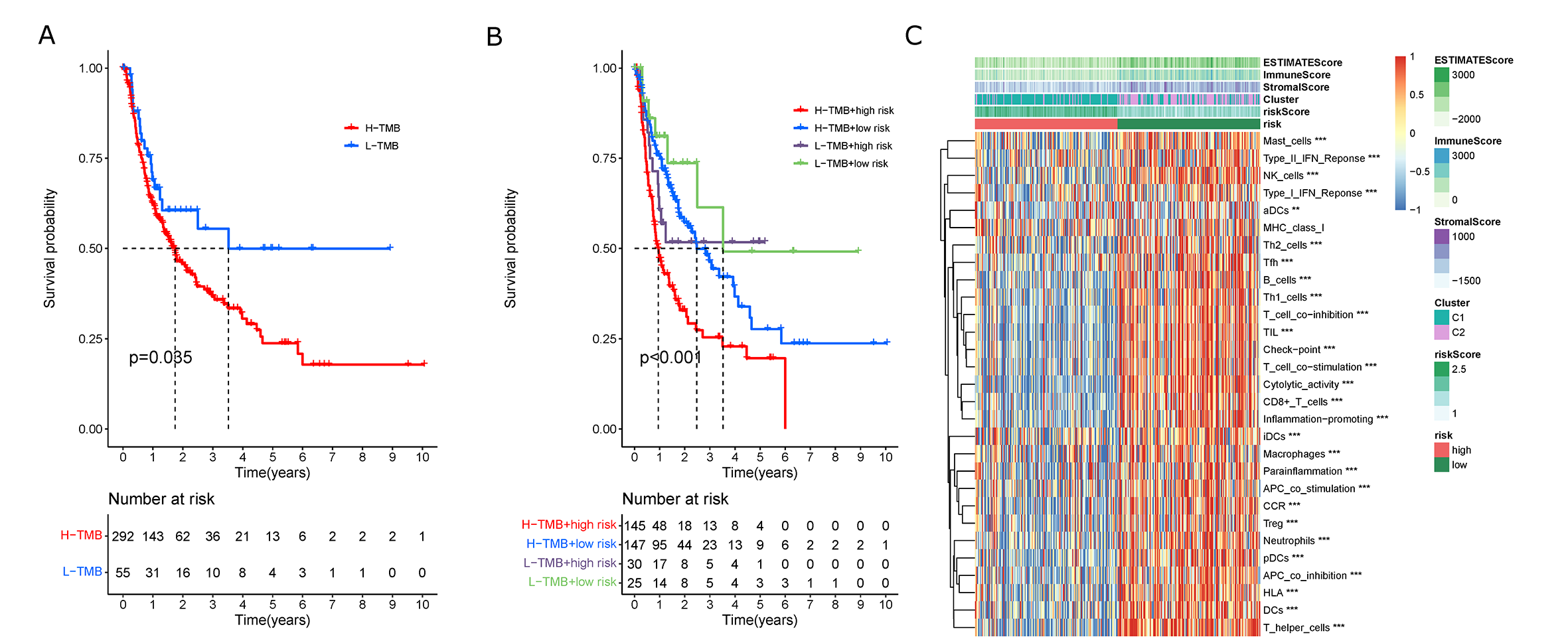


**Figure. S5.** **A.** Prognostic differences according to high or low TMB scores in TCGA. **B.** Comparison of ICDRM and TMB in predicting prognosis. **C.** Heatmap of immune infiltration differences between ICDRM subpopulations and ICD clusters in TCGA.


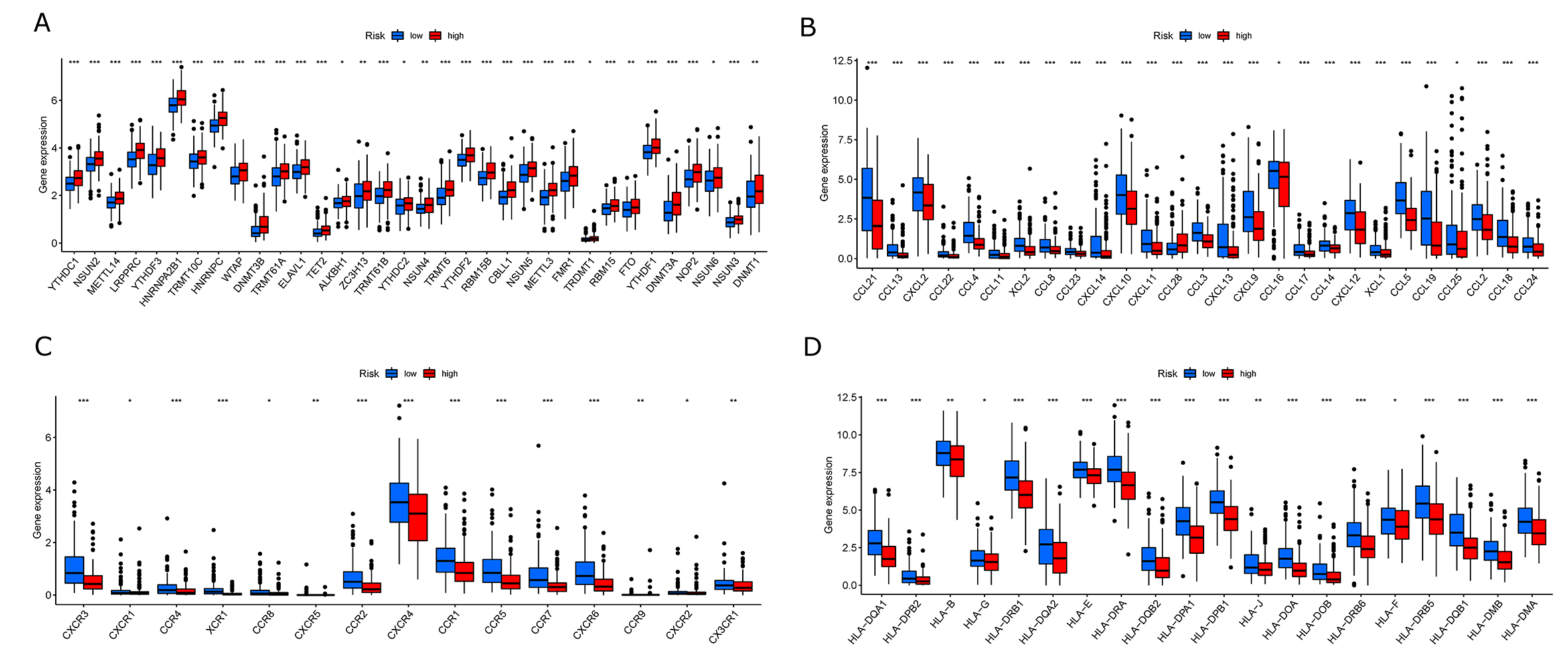


**Figure. S6. A.** Differences in the expression of RNA modification genes between ICDRM Rsk-low and Risk-high subpopulations. **B.** Differences in the expression of chemokine genes between ICDRM Risk-low and Risk-high subpopulations. **C.** Differences in the expression of receptor genes between ICDRM Risk-low and Risk-high subpopulations. **D.** Differences in the expression of HLA genes between ICDRM Risk-low and Risk-high subpopulations.


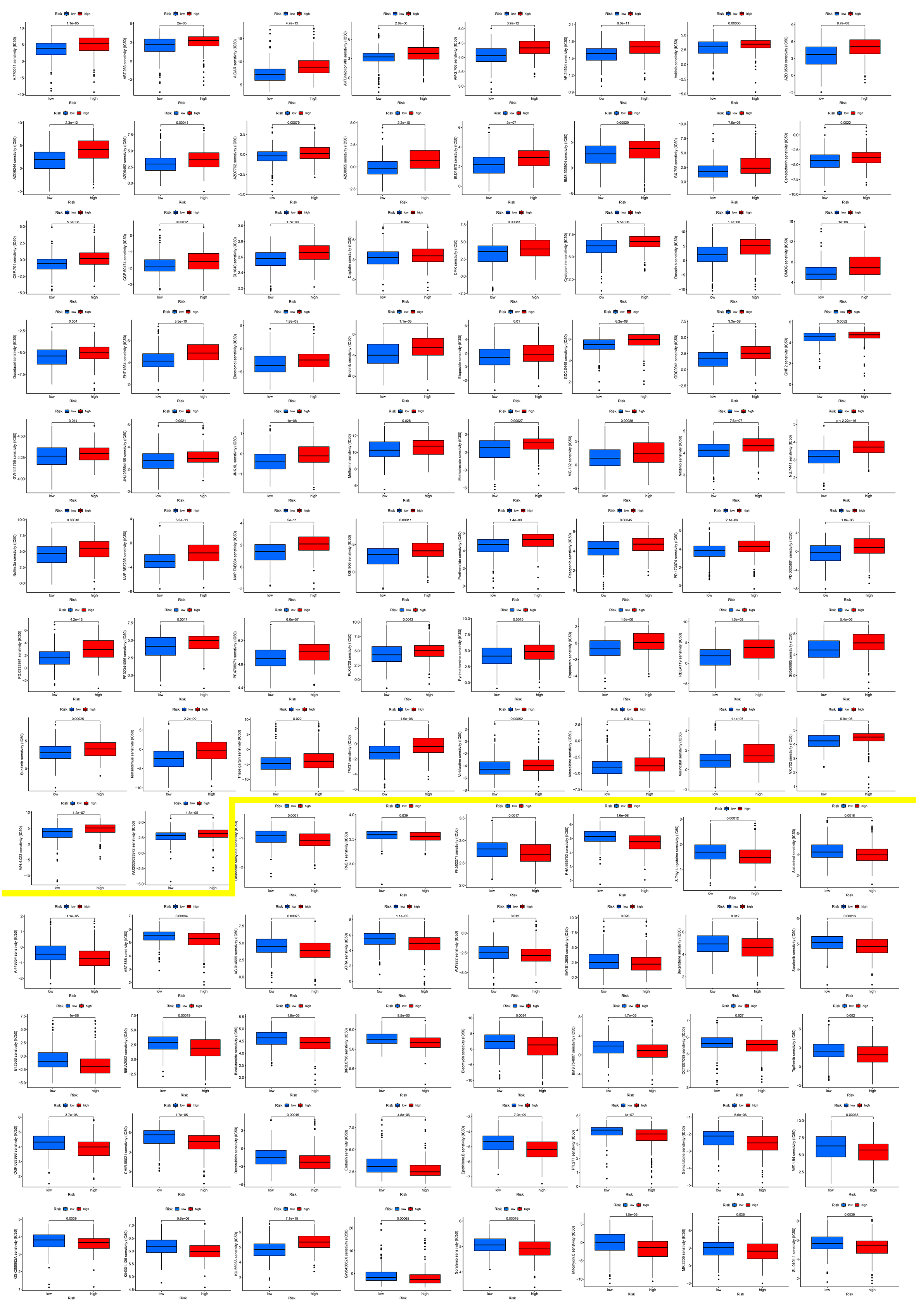


**Figure. S7.** Analysis of drug sensitivity between ICDRM Risk-low and Risk-high subpopulations about clinically used Chemotherapeutic and targeted drugs through R package "pRRophetic".


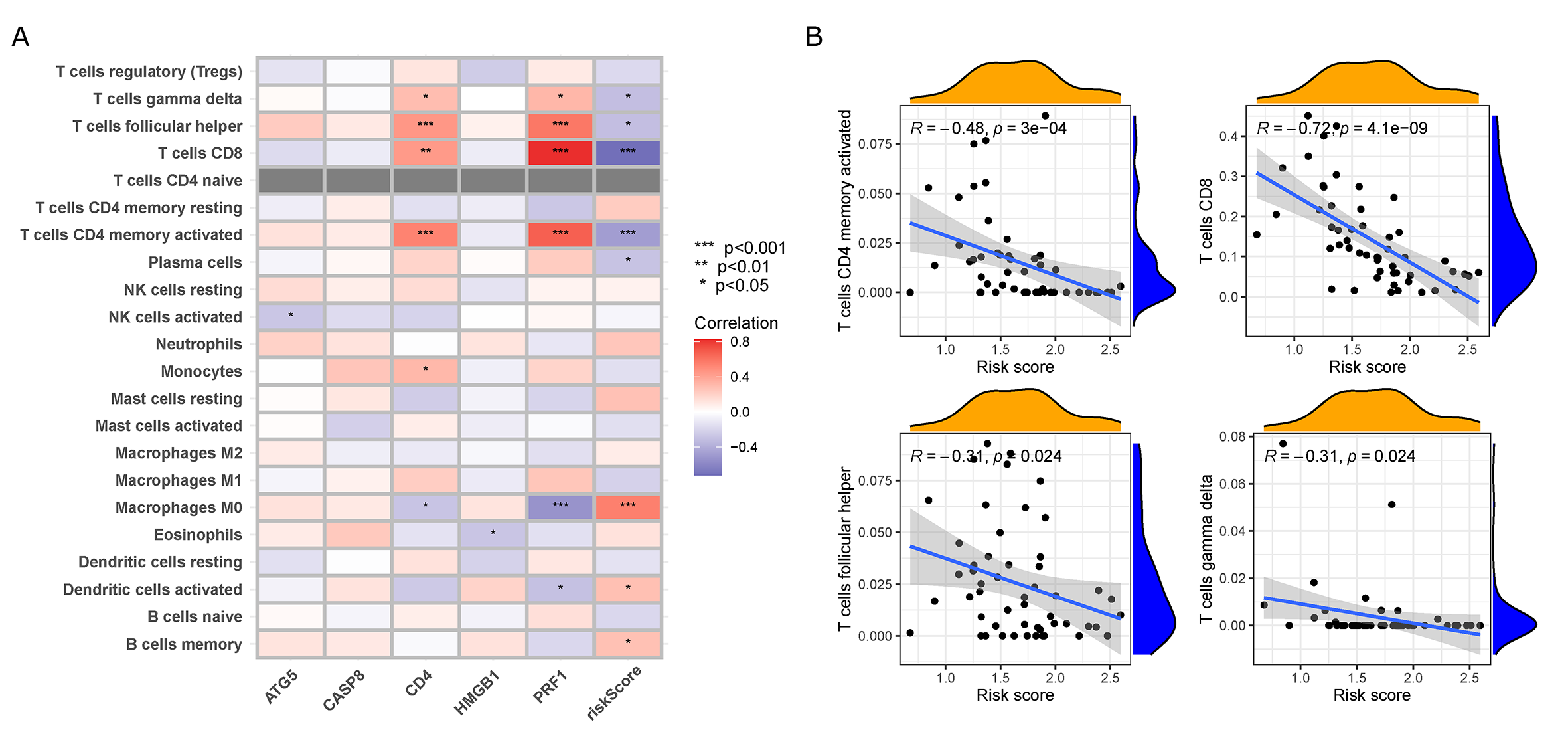


**Figure. S8.** **A.** The correlation of ICDRM with immune cells. **B.** Scatter plots show the correlation of ICDRM with the infiltration of activated CD4^+^ T memory, CD8^+^ T, T follicular helper, and γδ T cells.


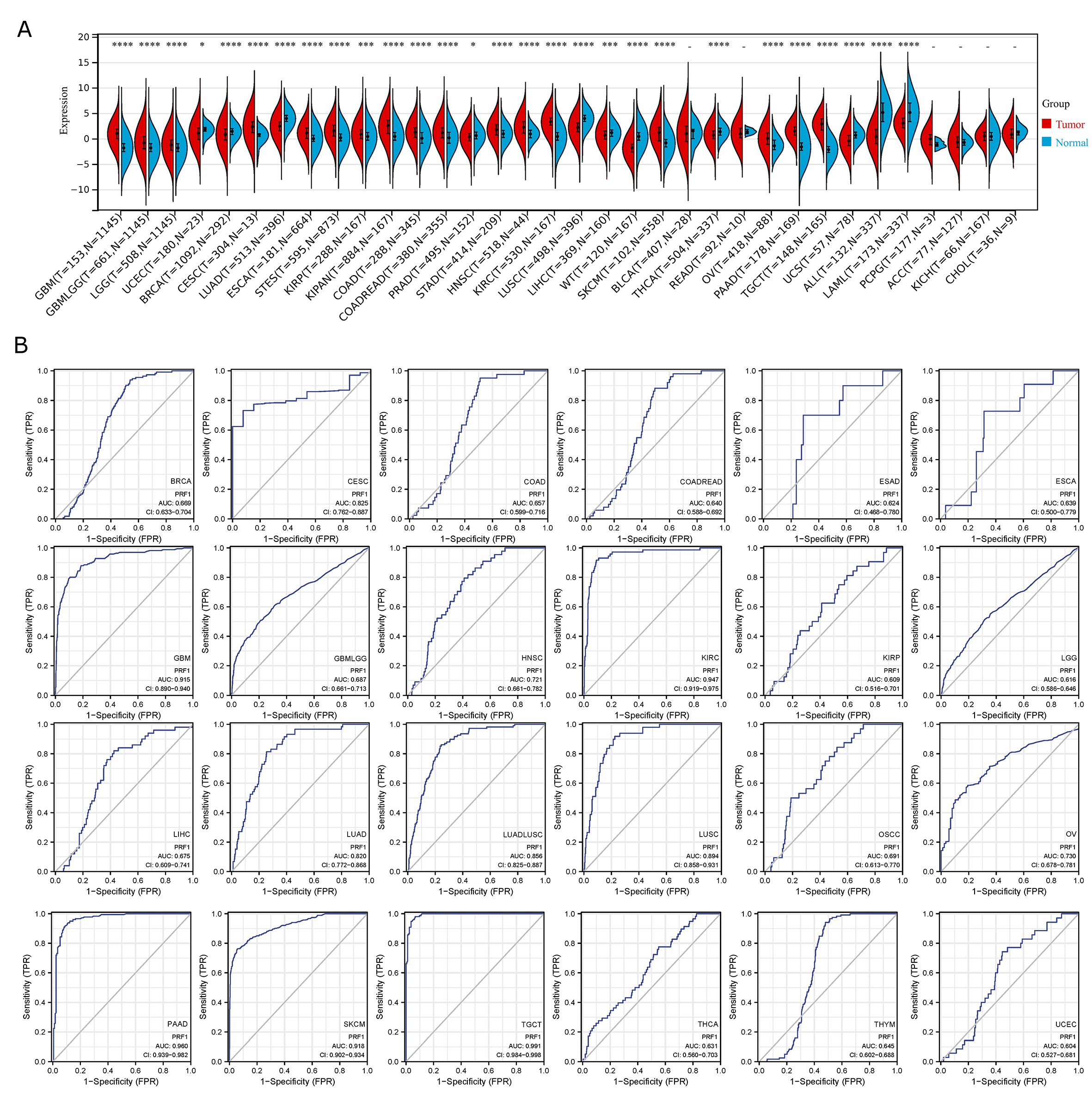


**Figure. S9. A.** Expression differences of PRF1 between normal and tumor samples in TCGA. **B.** ROC curves of PRF1 in predicting the diagnostic value in TCGA.


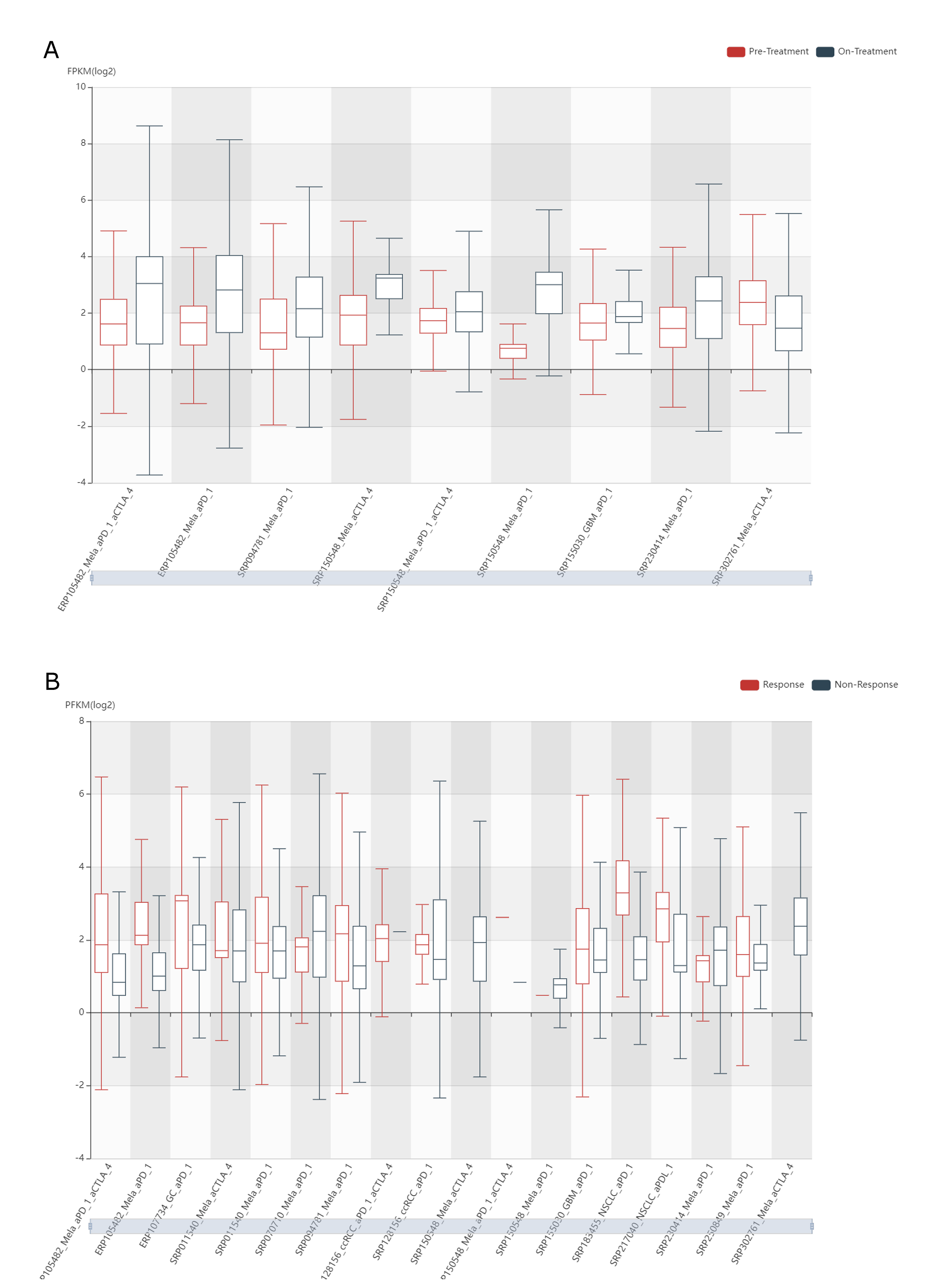


**Figure. S10.** **A.** Expression differences of PRF1 between Pre-treatment and On-treatment samples in each immunotherapy dataset. **B.** Expression differences of PRF1 between Response and Non-response based on Pre-treatment samples in all immunotherapy datasets.


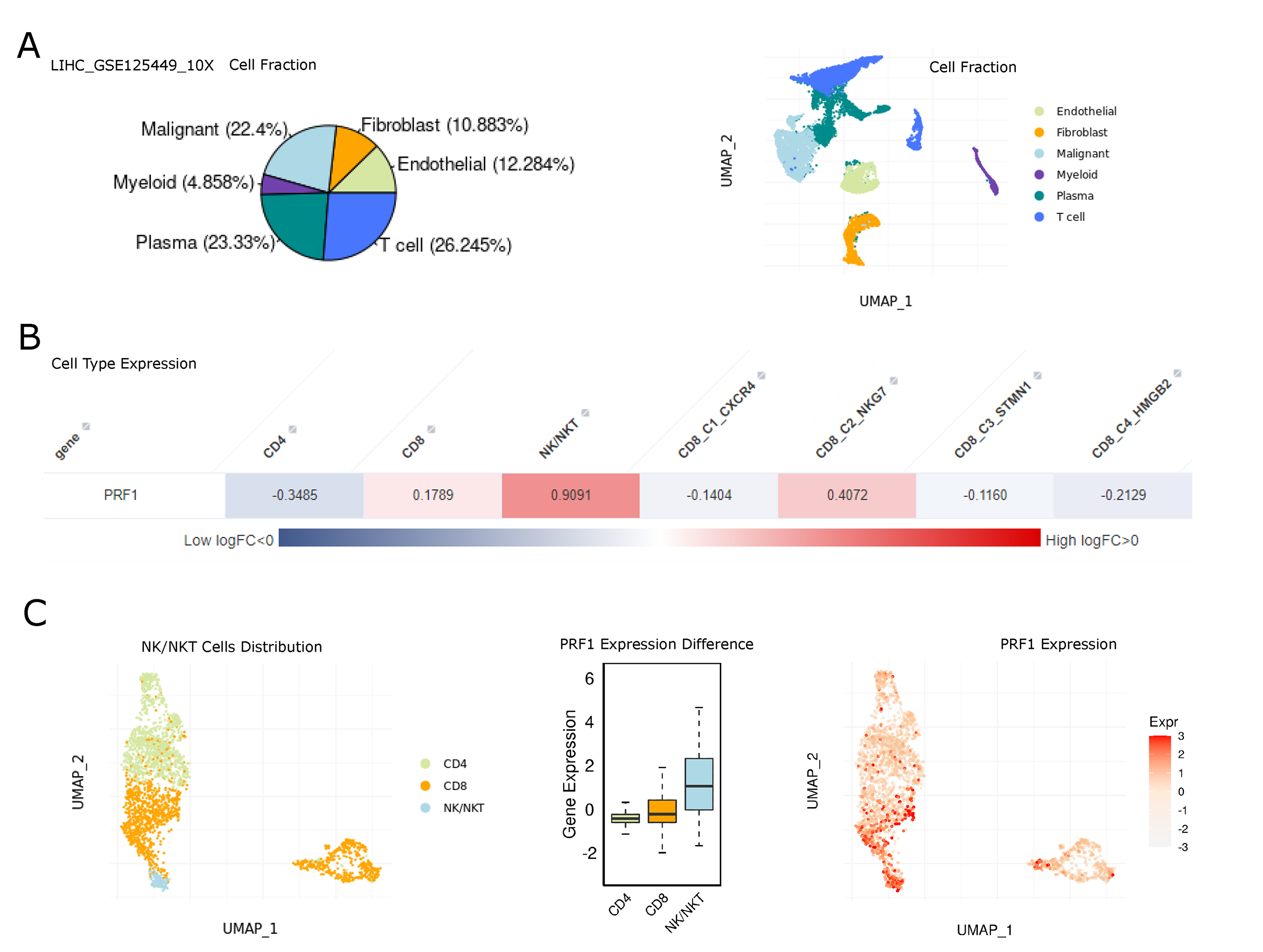


**Figure. S11.** **A.** The cell types and their distribution in HCC GSE125449 10x dataset. **B.** Relationship between PRF1 expression and immune cells in HCC GSE125449 10x dataset. **C.** The distribution of PRF1 in NK/NKT cells was analyzed using single-cell resolution.

## Supplementary Tables Legends

**Supplementary Table S1.** The sequences of qRT-PCR primers and shRNA sequences of BAX and HSP90AA1.

**Supplementary Table S2.** DEGs between ICD-low and ICD-high clusters.

**Supplementary Table S3.** GSEA enrichment analysis of the DEGs between ICD-low and ICD-high clusters.

**Supplementary Table S4.** DEGs between high-risk and low-risk subpopulations.

**Supplementary Table S5.** GSEA enrichment analysis of the DEGs between high-risk and low-risk subpopulations.

**Supplementary Table S6.** The intersection of the drugs was predicted using the R package "pRRophetic" and the cMAP website.

**Supplementary Table S7.** 1136 Co-expressed genes that were significantly related to PRF1.

**Supplementary Table S8.** 1813 DEGs of the PRF1 high and PRF1 low expression subpopulations.

**Supplementary Table S9.** 581 overlapping genes between 1813 DEGs and 1136 co-expressed genes.
