# Supplementary figures and images for: Analyzing molecular typing and clinical application of immunogenic cell death-related genes in hepatocellular carcinoma
Source: BMC Cancer. 2023 Jun 8;23:522. doi: 10.1186/s12885-023-10992-2 (PMC10249577; doi:10.1186/s12885-023-10992-2)

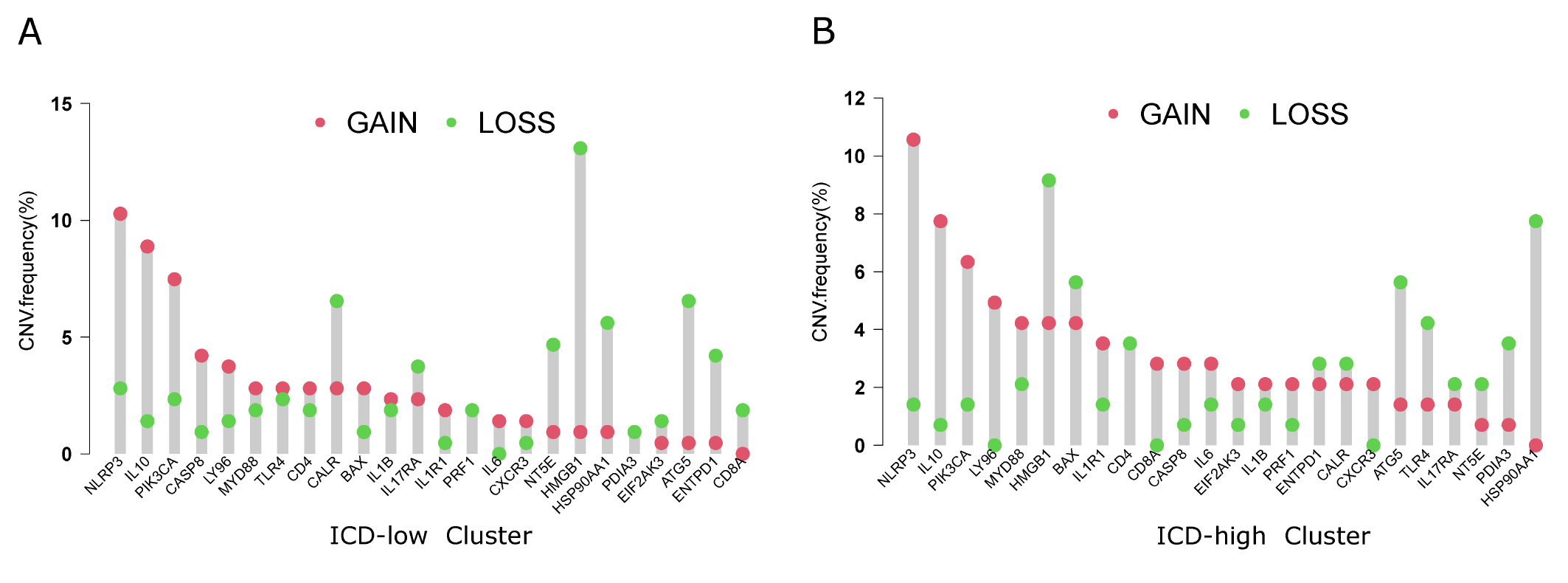

Supplement: Supplementary file 1 — Additional file 1: Figure S1. A. Frequenciesof CNV gain, loss, and non-CNV among ICDs in ICD-low clusters. B. Frequenciesof CNV gain, loss, and non-CNV among ICDs in ICD-high clusters. Figure S2. A. mRNAlevels of BAX in THLE-2 and HCC cells. B. mRNA levels of BAX in HepG2 and Huh7HCC cells after BAX was knocked down. C-D. A colony formation assay was used toexplore the function of BAX in HCC cells. Their representative images are shownin C. E-F. Knockdown of BAX inhibits HCC cell migration. Wound healing assayswere used to assess the migration of HepG2 and Huh7 cells after the BAXknockdown. Representative images are shown in E (* P<0.05, ** P<0.01, ***P<0.001).All experiments were repeated at least three times. Figure S3. A. Differencesin the expression of RNA modification genes between ICD-low and ICD-highclusters. B. Differences in the expression of chemokine genes between ICD-lowand ICD-high clusters. C. Differences in the expression of receptor genesbetween ICD-low and ICD-high clusters. D. Differences in the expression of HLAgenes between ICD-low and ICD-high clusters. Figure S4. A. Frequencies of CNVgain, loss, and non-CNV among ICDs in Risk-high clusters. B. Frequencies of CNVgain, loss, and non-CNV among ICDs in Risk-low clusters. Figure S5. A.Prognostic differences according to high or low TMB scores in TCGA. B.Comparison of ICDRM and TMB in predicting prognosis. C. Heatmap of immuneinfiltration differences between ICDRM subpopulations and ICD clusters in TCGA.Figure S6. A. Differences in the expression of RNA modification genes between ICDRMRsk-low and Risk-high subpopulations. B. Differences in the expression ofchemokine genes between ICDRM Risk-low and Risk-high subpopulations. C.Differences in the expression of receptor genes between ICDRM Risk-low and Risk-highsubpopulations. D. Differences in the expression of HLA genes between ICDRM Risk-lowand Risk-high subpopulations. Figure S7. Analysis of drug sensitivity between ICDRMRisk-low and Risk-high subpo [file 12885_2023_10992_MOESM1_ESM.zip › Supplementary Figure/Supplementary FIG 1.tif]

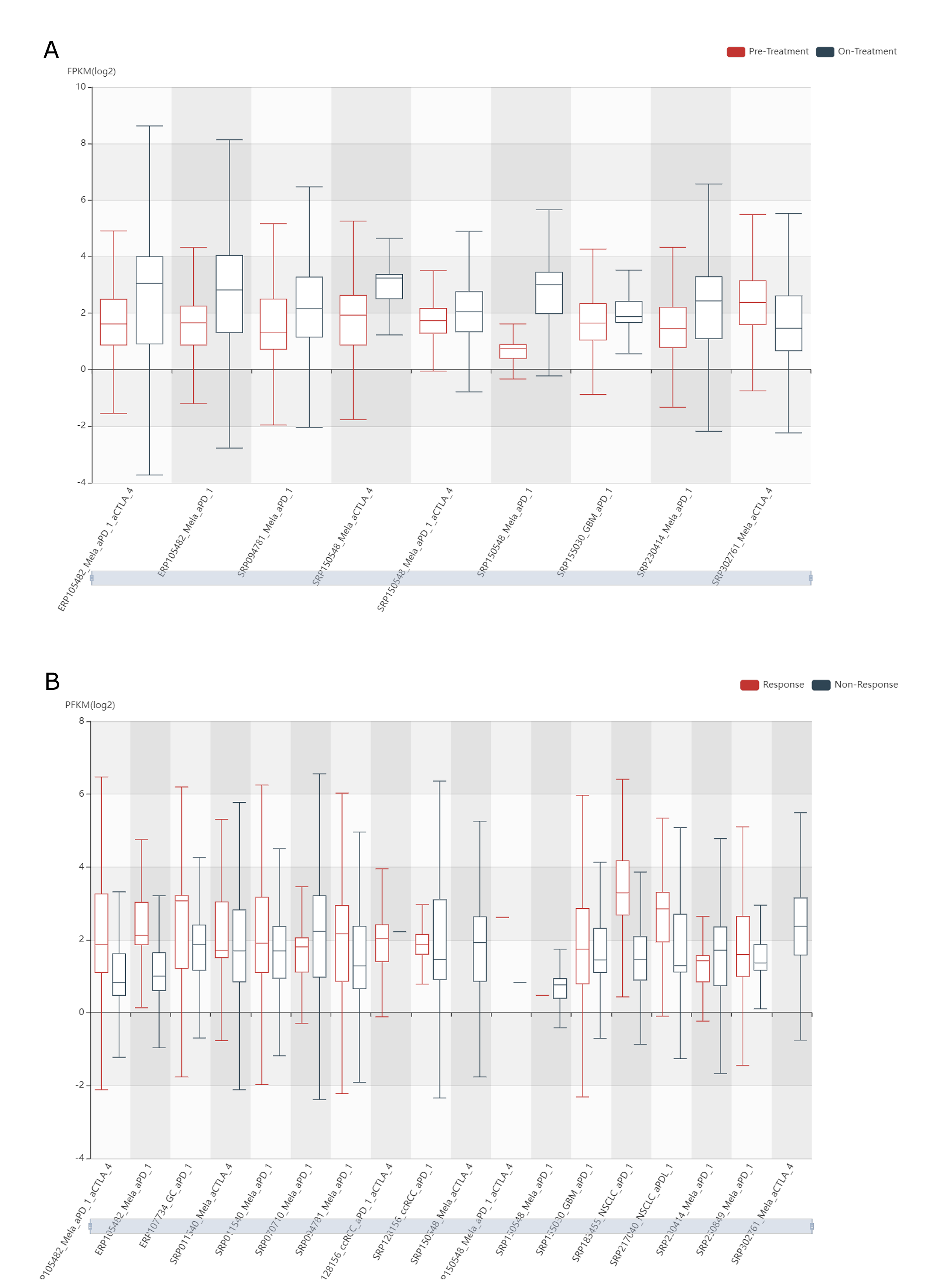

Supplement: Supplementary file 1 — Additional file 1: Figure S1. A. Frequenciesof CNV gain, loss, and non-CNV among ICDs in ICD-low clusters. B. Frequenciesof CNV gain, loss, and non-CNV among ICDs in ICD-high clusters. Figure S2. A. mRNAlevels of BAX in THLE-2 and HCC cells. B. mRNA levels of BAX in HepG2 and Huh7HCC cells after BAX was knocked down. C-D. A colony formation assay was used toexplore the function of BAX in HCC cells. Their representative images are shownin C. E-F. Knockdown of BAX inhibits HCC cell migration. Wound healing assayswere used to assess the migration of HepG2 and Huh7 cells after the BAXknockdown. Representative images are shown in E (* P<0.05, ** P<0.01, ***P<0.001).All experiments were repeated at least three times. Figure S3. A. Differencesin the expression of RNA modification genes between ICD-low and ICD-highclusters. B. Differences in the expression of chemokine genes between ICD-lowand ICD-high clusters. C. Differences in the expression of receptor genesbetween ICD-low and ICD-high clusters. D. Differences in the expression of HLAgenes between ICD-low and ICD-high clusters. Figure S4. A. Frequencies of CNVgain, loss, and non-CNV among ICDs in Risk-high clusters. B. Frequencies of CNVgain, loss, and non-CNV among ICDs in Risk-low clusters. Figure S5. A.Prognostic differences according to high or low TMB scores in TCGA. B.Comparison of ICDRM and TMB in predicting prognosis. C. Heatmap of immuneinfiltration differences between ICDRM subpopulations and ICD clusters in TCGA.Figure S6. A. Differences in the expression of RNA modification genes between ICDRMRsk-low and Risk-high subpopulations. B. Differences in the expression ofchemokine genes between ICDRM Risk-low and Risk-high subpopulations. C.Differences in the expression of receptor genes between ICDRM Risk-low and Risk-highsubpopulations. D. Differences in the expression of HLA genes between ICDRM Risk-lowand Risk-high subpopulations. Figure S7. Analysis of drug sensitivity between ICDRMRisk-low and Risk-high subpo [file 12885_2023_10992_MOESM1_ESM.zip › Supplementary Figure/Supplementary FIG 10.tiff]

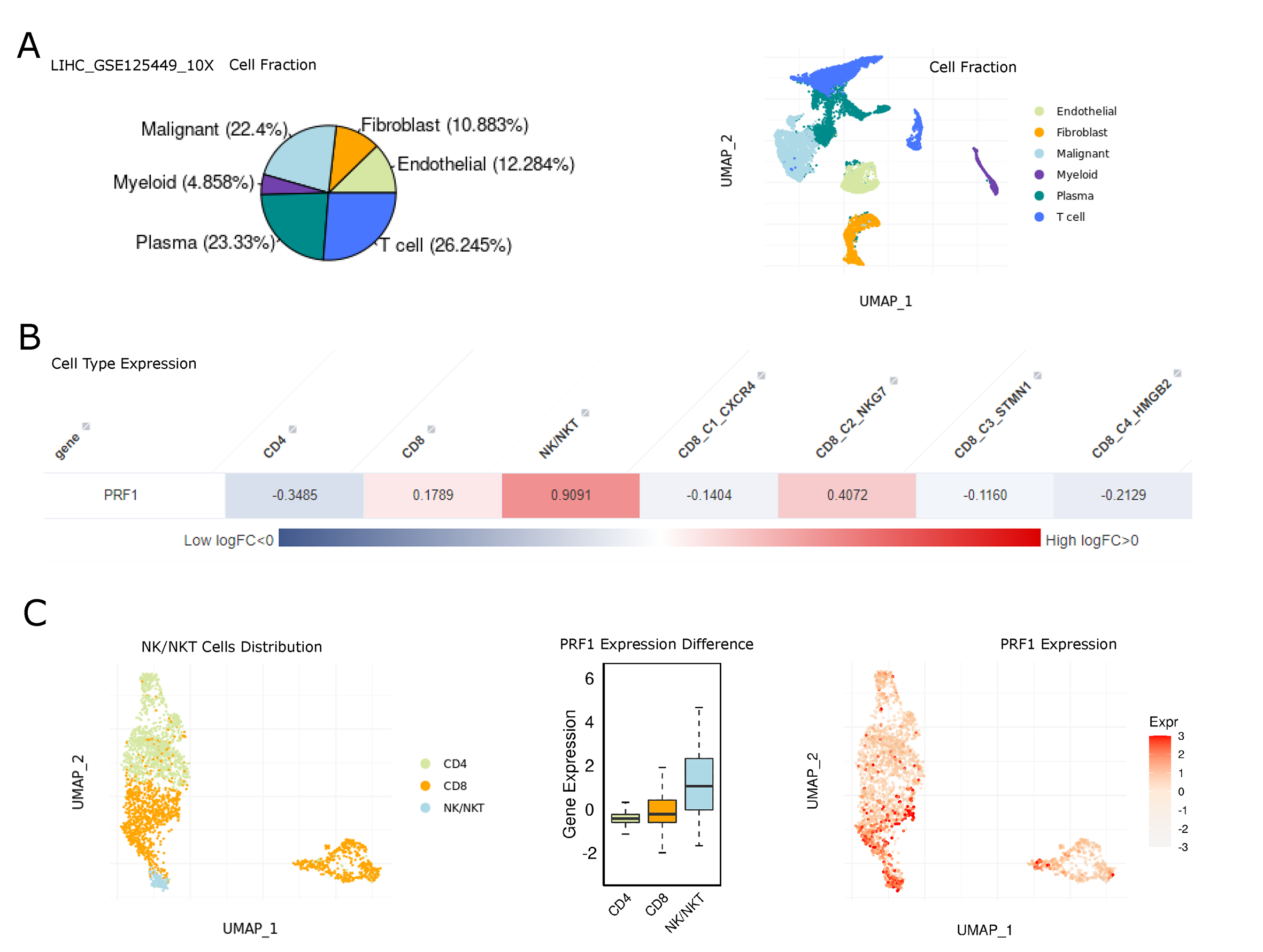

Supplement: Supplementary file 1 — Additional file 1: Figure S1. A. Frequenciesof CNV gain, loss, and non-CNV among ICDs in ICD-low clusters. B. Frequenciesof CNV gain, loss, and non-CNV among ICDs in ICD-high clusters. Figure S2. A. mRNAlevels of BAX in THLE-2 and HCC cells. B. mRNA levels of BAX in HepG2 and Huh7HCC cells after BAX was knocked down. C-D. A colony formation assay was used toexplore the function of BAX in HCC cells. Their representative images are shownin C. E-F. Knockdown of BAX inhibits HCC cell migration. Wound healing assayswere used to assess the migration of HepG2 and Huh7 cells after the BAXknockdown. Representative images are shown in E (* P<0.05, ** P<0.01, ***P<0.001).All experiments were repeated at least three times. Figure S3. A. Differencesin the expression of RNA modification genes between ICD-low and ICD-highclusters. B. Differences in the expression of chemokine genes between ICD-lowand ICD-high clusters. C. Differences in the expression of receptor genesbetween ICD-low and ICD-high clusters. D. Differences in the expression of HLAgenes between ICD-low and ICD-high clusters. Figure S4. A. Frequencies of CNVgain, loss, and non-CNV among ICDs in Risk-high clusters. B. Frequencies of CNVgain, loss, and non-CNV among ICDs in Risk-low clusters. Figure S5. A.Prognostic differences according to high or low TMB scores in TCGA. B.Comparison of ICDRM and TMB in predicting prognosis. C. Heatmap of immuneinfiltration differences between ICDRM subpopulations and ICD clusters in TCGA.Figure S6. A. Differences in the expression of RNA modification genes between ICDRMRsk-low and Risk-high subpopulations. B. Differences in the expression ofchemokine genes between ICDRM Risk-low and Risk-high subpopulations. C.Differences in the expression of receptor genes between ICDRM Risk-low and Risk-highsubpopulations. D. Differences in the expression of HLA genes between ICDRM Risk-lowand Risk-high subpopulations. Figure S7. Analysis of drug sensitivity between ICDRMRisk-low and Risk-high subpo [file 12885_2023_10992_MOESM1_ESM.zip › Supplementary Figure/Supplementary FIG 11.tiff]

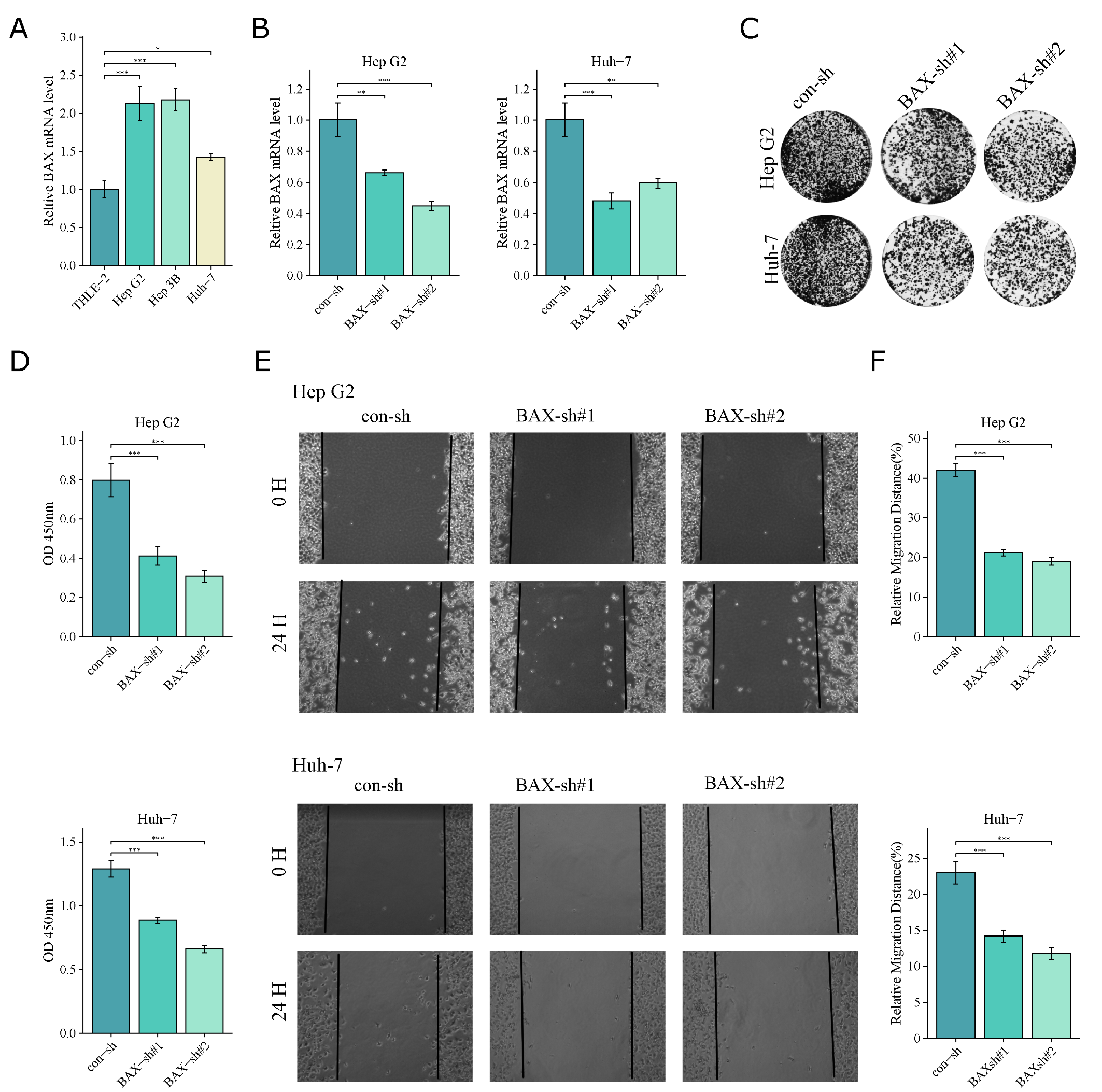

Supplement: Supplementary file 1 — Additional file 1: Figure S1. A. Frequenciesof CNV gain, loss, and non-CNV among ICDs in ICD-low clusters. B. Frequenciesof CNV gain, loss, and non-CNV among ICDs in ICD-high clusters. Figure S2. A. mRNAlevels of BAX in THLE-2 and HCC cells. B. mRNA levels of BAX in HepG2 and Huh7HCC cells after BAX was knocked down. C-D. A colony formation assay was used toexplore the function of BAX in HCC cells. Their representative images are shownin C. E-F. Knockdown of BAX inhibits HCC cell migration. Wound healing assayswere used to assess the migration of HepG2 and Huh7 cells after the BAXknockdown. Representative images are shown in E (* P<0.05, ** P<0.01, ***P<0.001).All experiments were repeated at least three times. Figure S3. A. Differencesin the expression of RNA modification genes between ICD-low and ICD-highclusters. B. Differences in the expression of chemokine genes between ICD-lowand ICD-high clusters. C. Differences in the expression of receptor genesbetween ICD-low and ICD-high clusters. D. Differences in the expression of HLAgenes between ICD-low and ICD-high clusters. Figure S4. A. Frequencies of CNVgain, loss, and non-CNV among ICDs in Risk-high clusters. B. Frequencies of CNVgain, loss, and non-CNV among ICDs in Risk-low clusters. Figure S5. A.Prognostic differences according to high or low TMB scores in TCGA. B.Comparison of ICDRM and TMB in predicting prognosis. C. Heatmap of immuneinfiltration differences between ICDRM subpopulations and ICD clusters in TCGA.Figure S6. A. Differences in the expression of RNA modification genes between ICDRMRsk-low and Risk-high subpopulations. B. Differences in the expression ofchemokine genes between ICDRM Risk-low and Risk-high subpopulations. C.Differences in the expression of receptor genes between ICDRM Risk-low and Risk-highsubpopulations. D. Differences in the expression of HLA genes between ICDRM Risk-lowand Risk-high subpopulations. Figure S7. Analysis of drug sensitivity between ICDRMRisk-low and Risk-high subpo [file 12885_2023_10992_MOESM1_ESM.zip › Supplementary Figure/Supplementary FIG 2.tif]

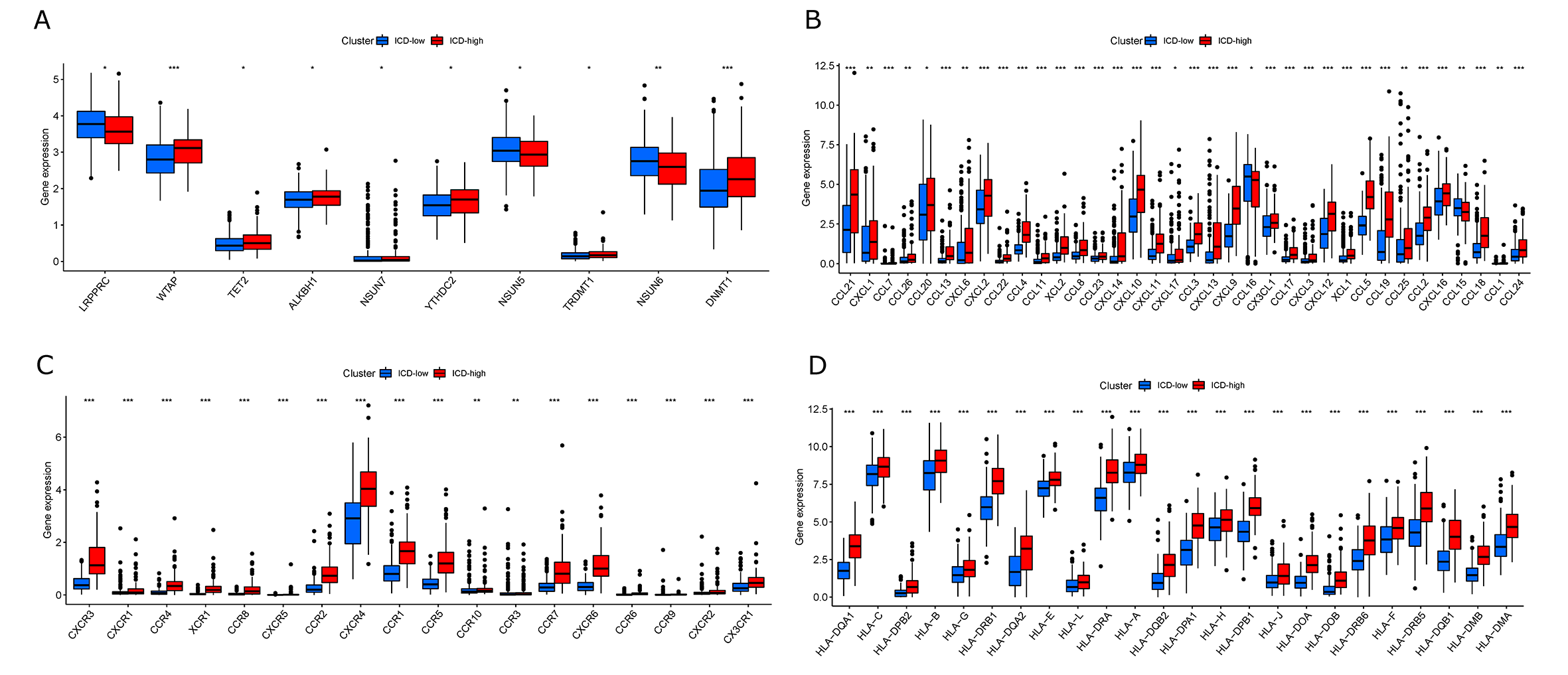

Supplement: Supplementary file 1 — Additional file 1: Figure S1. A. Frequenciesof CNV gain, loss, and non-CNV among ICDs in ICD-low clusters. B. Frequenciesof CNV gain, loss, and non-CNV among ICDs in ICD-high clusters. Figure S2. A. mRNAlevels of BAX in THLE-2 and HCC cells. B. mRNA levels of BAX in HepG2 and Huh7HCC cells after BAX was knocked down. C-D. A colony formation assay was used toexplore the function of BAX in HCC cells. Their representative images are shownin C. E-F. Knockdown of BAX inhibits HCC cell migration. Wound healing assayswere used to assess the migration of HepG2 and Huh7 cells after the BAXknockdown. Representative images are shown in E (* P<0.05, ** P<0.01, ***P<0.001).All experiments were repeated at least three times. Figure S3. A. Differencesin the expression of RNA modification genes between ICD-low and ICD-highclusters. B. Differences in the expression of chemokine genes between ICD-lowand ICD-high clusters. C. Differences in the expression of receptor genesbetween ICD-low and ICD-high clusters. D. Differences in the expression of HLAgenes between ICD-low and ICD-high clusters. Figure S4. A. Frequencies of CNVgain, loss, and non-CNV among ICDs in Risk-high clusters. B. Frequencies of CNVgain, loss, and non-CNV among ICDs in Risk-low clusters. Figure S5. A.Prognostic differences according to high or low TMB scores in TCGA. B.Comparison of ICDRM and TMB in predicting prognosis. C. Heatmap of immuneinfiltration differences between ICDRM subpopulations and ICD clusters in TCGA.Figure S6. A. Differences in the expression of RNA modification genes between ICDRMRsk-low and Risk-high subpopulations. B. Differences in the expression ofchemokine genes between ICDRM Risk-low and Risk-high subpopulations. C.Differences in the expression of receptor genes between ICDRM Risk-low and Risk-highsubpopulations. D. Differences in the expression of HLA genes between ICDRM Risk-lowand Risk-high subpopulations. Figure S7. Analysis of drug sensitivity between ICDRMRisk-low and Risk-high subpo [file 12885_2023_10992_MOESM1_ESM.zip › Supplementary Figure/Supplementary FIG 3.tiff]

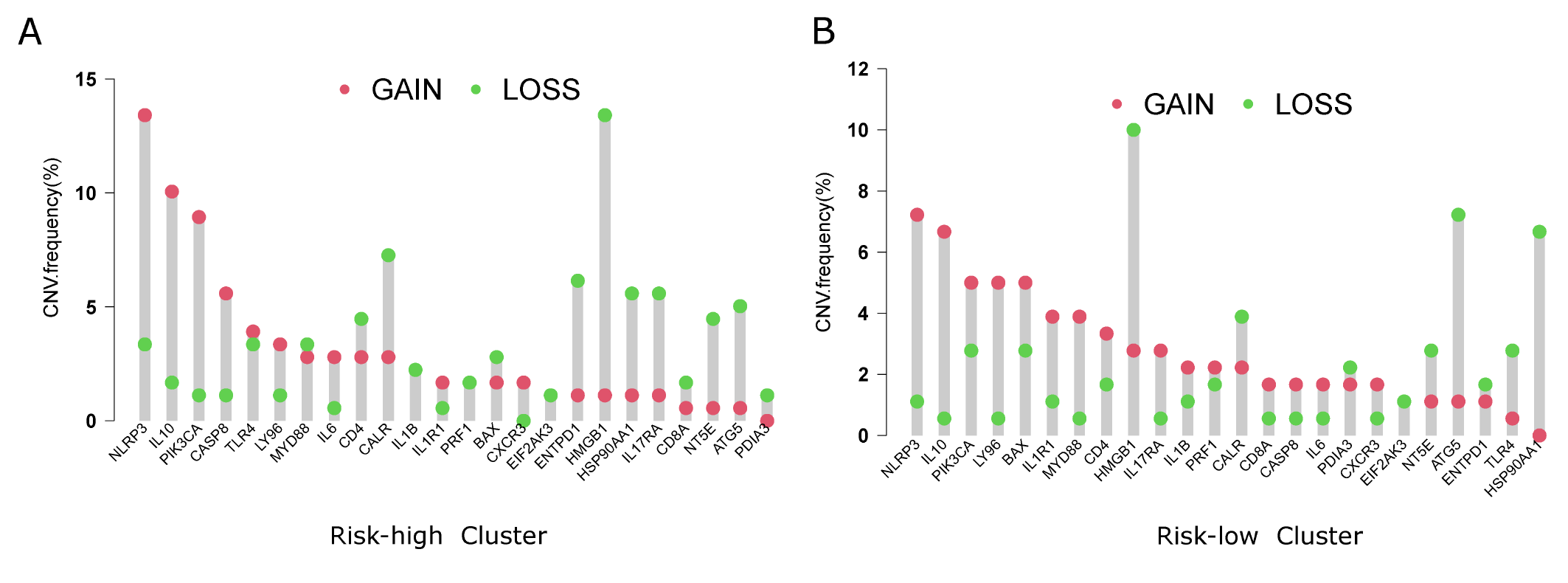

Supplement: Supplementary file 1 — Additional file 1: Figure S1. A. Frequenciesof CNV gain, loss, and non-CNV among ICDs in ICD-low clusters. B. Frequenciesof CNV gain, loss, and non-CNV among ICDs in ICD-high clusters. Figure S2. A. mRNAlevels of BAX in THLE-2 and HCC cells. B. mRNA levels of BAX in HepG2 and Huh7HCC cells after BAX was knocked down. C-D. A colony formation assay was used toexplore the function of BAX in HCC cells. Their representative images are shownin C. E-F. Knockdown of BAX inhibits HCC cell migration. Wound healing assayswere used to assess the migration of HepG2 and Huh7 cells after the BAXknockdown. Representative images are shown in E (* P<0.05, ** P<0.01, ***P<0.001).All experiments were repeated at least three times. Figure S3. A. Differencesin the expression of RNA modification genes between ICD-low and ICD-highclusters. B. Differences in the expression of chemokine genes between ICD-lowand ICD-high clusters. C. Differences in the expression of receptor genesbetween ICD-low and ICD-high clusters. D. Differences in the expression of HLAgenes between ICD-low and ICD-high clusters. Figure S4. A. Frequencies of CNVgain, loss, and non-CNV among ICDs in Risk-high clusters. B. Frequencies of CNVgain, loss, and non-CNV among ICDs in Risk-low clusters. Figure S5. A.Prognostic differences according to high or low TMB scores in TCGA. B.Comparison of ICDRM and TMB in predicting prognosis. C. Heatmap of immuneinfiltration differences between ICDRM subpopulations and ICD clusters in TCGA.Figure S6. A. Differences in the expression of RNA modification genes between ICDRMRsk-low and Risk-high subpopulations. B. Differences in the expression ofchemokine genes between ICDRM Risk-low and Risk-high subpopulations. C.Differences in the expression of receptor genes between ICDRM Risk-low and Risk-highsubpopulations. D. Differences in the expression of HLA genes between ICDRM Risk-lowand Risk-high subpopulations. Figure S7. Analysis of drug sensitivity between ICDRMRisk-low and Risk-high subpo [file 12885_2023_10992_MOESM1_ESM.zip › Supplementary Figure/Supplementary FIG 4.tif]

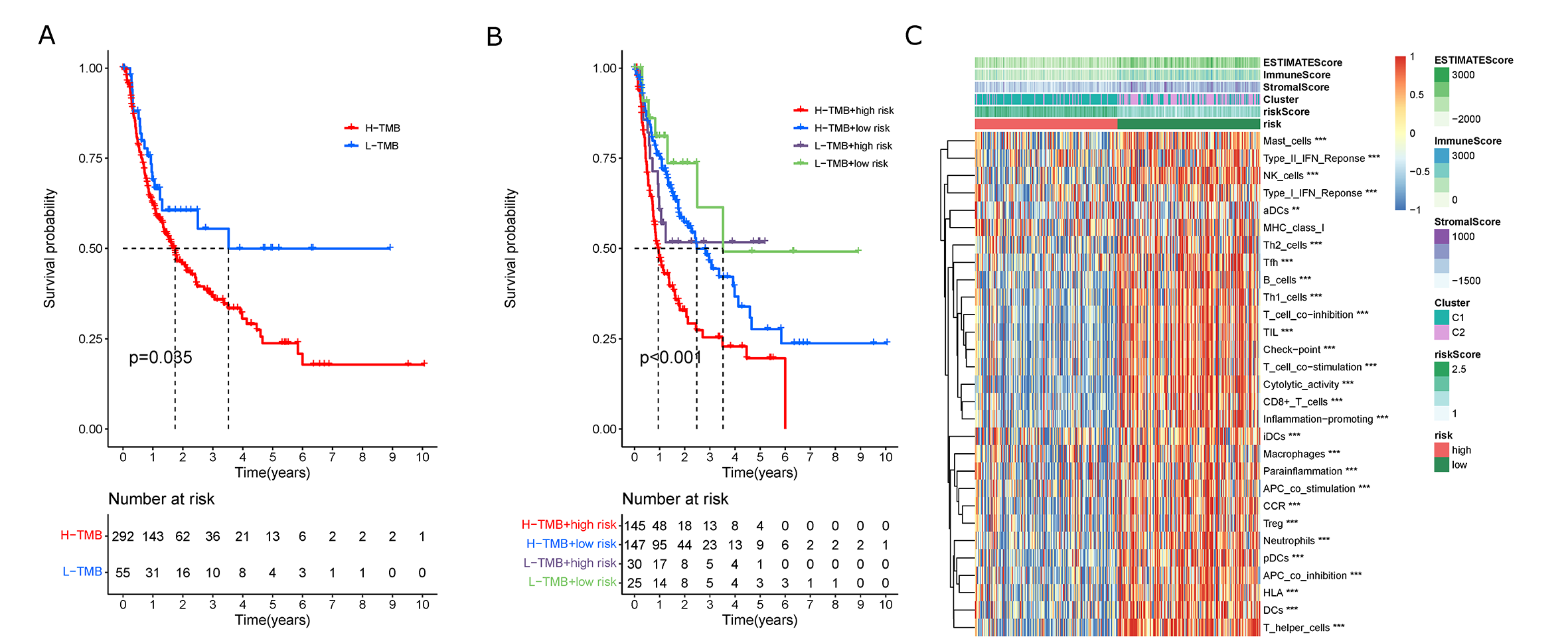

Supplement: Supplementary file 1 — Additional file 1: Figure S1. A. Frequenciesof CNV gain, loss, and non-CNV among ICDs in ICD-low clusters. B. Frequenciesof CNV gain, loss, and non-CNV among ICDs in ICD-high clusters. Figure S2. A. mRNAlevels of BAX in THLE-2 and HCC cells. B. mRNA levels of BAX in HepG2 and Huh7HCC cells after BAX was knocked down. C-D. A colony formation assay was used toexplore the function of BAX in HCC cells. Their representative images are shownin C. E-F. Knockdown of BAX inhibits HCC cell migration. Wound healing assayswere used to assess the migration of HepG2 and Huh7 cells after the BAXknockdown. Representative images are shown in E (* P<0.05, ** P<0.01, ***P<0.001).All experiments were repeated at least three times. Figure S3. A. Differencesin the expression of RNA modification genes between ICD-low and ICD-highclusters. B. Differences in the expression of chemokine genes between ICD-lowand ICD-high clusters. C. Differences in the expression of receptor genesbetween ICD-low and ICD-high clusters. D. Differences in the expression of HLAgenes between ICD-low and ICD-high clusters. Figure S4. A. Frequencies of CNVgain, loss, and non-CNV among ICDs in Risk-high clusters. B. Frequencies of CNVgain, loss, and non-CNV among ICDs in Risk-low clusters. Figure S5. A.Prognostic differences according to high or low TMB scores in TCGA. B.Comparison of ICDRM and TMB in predicting prognosis. C. Heatmap of immuneinfiltration differences between ICDRM subpopulations and ICD clusters in TCGA.Figure S6. A. Differences in the expression of RNA modification genes between ICDRMRsk-low and Risk-high subpopulations. B. Differences in the expression ofchemokine genes between ICDRM Risk-low and Risk-high subpopulations. C.Differences in the expression of receptor genes between ICDRM Risk-low and Risk-highsubpopulations. D. Differences in the expression of HLA genes between ICDRM Risk-lowand Risk-high subpopulations. Figure S7. Analysis of drug sensitivity between ICDRMRisk-low and Risk-high subpo [file 12885_2023_10992_MOESM1_ESM.zip › Supplementary Figure/Supplementary FIG 5.tiff]

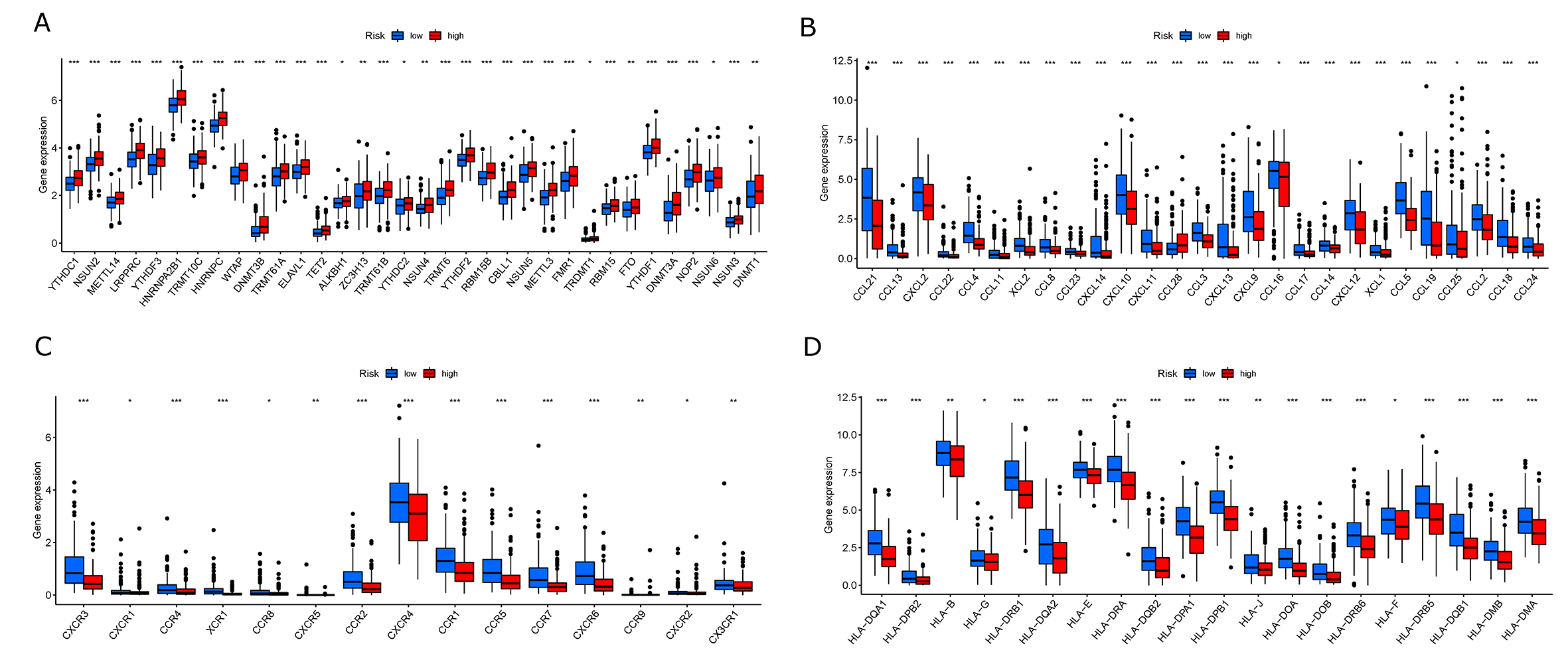

Supplement: Supplementary file 1 — Additional file 1: Figure S1. A. Frequenciesof CNV gain, loss, and non-CNV among ICDs in ICD-low clusters. B. Frequenciesof CNV gain, loss, and non-CNV among ICDs in ICD-high clusters. Figure S2. A. mRNAlevels of BAX in THLE-2 and HCC cells. B. mRNA levels of BAX in HepG2 and Huh7HCC cells after BAX was knocked down. C-D. A colony formation assay was used toexplore the function of BAX in HCC cells. Their representative images are shownin C. E-F. Knockdown of BAX inhibits HCC cell migration. Wound healing assayswere used to assess the migration of HepG2 and Huh7 cells after the BAXknockdown. Representative images are shown in E (* P<0.05, ** P<0.01, ***P<0.001).All experiments were repeated at least three times. Figure S3. A. Differencesin the expression of RNA modification genes between ICD-low and ICD-highclusters. B. Differences in the expression of chemokine genes between ICD-lowand ICD-high clusters. C. Differences in the expression of receptor genesbetween ICD-low and ICD-high clusters. D. Differences in the expression of HLAgenes between ICD-low and ICD-high clusters. Figure S4. A. Frequencies of CNVgain, loss, and non-CNV among ICDs in Risk-high clusters. B. Frequencies of CNVgain, loss, and non-CNV among ICDs in Risk-low clusters. Figure S5. A.Prognostic differences according to high or low TMB scores in TCGA. B.Comparison of ICDRM and TMB in predicting prognosis. C. Heatmap of immuneinfiltration differences between ICDRM subpopulations and ICD clusters in TCGA.Figure S6. A. Differences in the expression of RNA modification genes between ICDRMRsk-low and Risk-high subpopulations. B. Differences in the expression ofchemokine genes between ICDRM Risk-low and Risk-high subpopulations. C.Differences in the expression of receptor genes between ICDRM Risk-low and Risk-highsubpopulations. D. Differences in the expression of HLA genes between ICDRM Risk-lowand Risk-high subpopulations. Figure S7. Analysis of drug sensitivity between ICDRMRisk-low and Risk-high subpo [file 12885_2023_10992_MOESM1_ESM.zip › Supplementary Figure/Supplementary FIG 6.tiff]

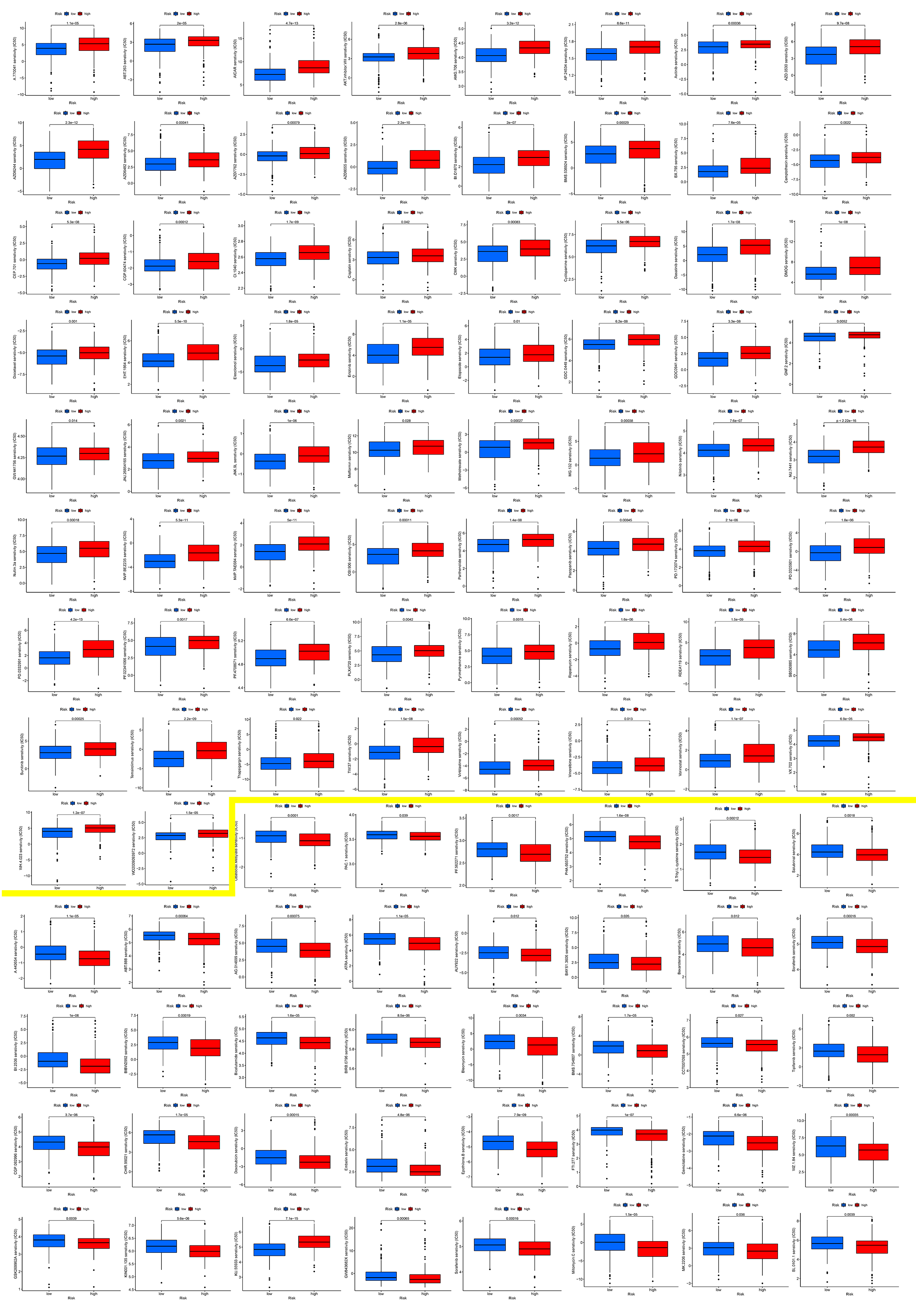

Supplement: Supplementary file 1 — Additional file 1: Figure S1. A. Frequenciesof CNV gain, loss, and non-CNV among ICDs in ICD-low clusters. B. Frequenciesof CNV gain, loss, and non-CNV among ICDs in ICD-high clusters. Figure S2. A. mRNAlevels of BAX in THLE-2 and HCC cells. B. mRNA levels of BAX in HepG2 and Huh7HCC cells after BAX was knocked down. C-D. A colony formation assay was used toexplore the function of BAX in HCC cells. Their representative images are shownin C. E-F. Knockdown of BAX inhibits HCC cell migration. Wound healing assayswere used to assess the migration of HepG2 and Huh7 cells after the BAXknockdown. Representative images are shown in E (* P<0.05, ** P<0.01, ***P<0.001).All experiments were repeated at least three times. Figure S3. A. Differencesin the expression of RNA modification genes between ICD-low and ICD-highclusters. B. Differences in the expression of chemokine genes between ICD-lowand ICD-high clusters. C. Differences in the expression of receptor genesbetween ICD-low and ICD-high clusters. D. Differences in the expression of HLAgenes between ICD-low and ICD-high clusters. Figure S4. A. Frequencies of CNVgain, loss, and non-CNV among ICDs in Risk-high clusters. B. Frequencies of CNVgain, loss, and non-CNV among ICDs in Risk-low clusters. Figure S5. A.Prognostic differences according to high or low TMB scores in TCGA. B.Comparison of ICDRM and TMB in predicting prognosis. C. Heatmap of immuneinfiltration differences between ICDRM subpopulations and ICD clusters in TCGA.Figure S6. A. Differences in the expression of RNA modification genes between ICDRMRsk-low and Risk-high subpopulations. B. Differences in the expression ofchemokine genes between ICDRM Risk-low and Risk-high subpopulations. C.Differences in the expression of receptor genes between ICDRM Risk-low and Risk-highsubpopulations. D. Differences in the expression of HLA genes between ICDRM Risk-lowand Risk-high subpopulations. Figure S7. Analysis of drug sensitivity between ICDRMRisk-low and Risk-high subpo [file 12885_2023_10992_MOESM1_ESM.zip › Supplementary Figure/Supplementary FIG 7.tiff]

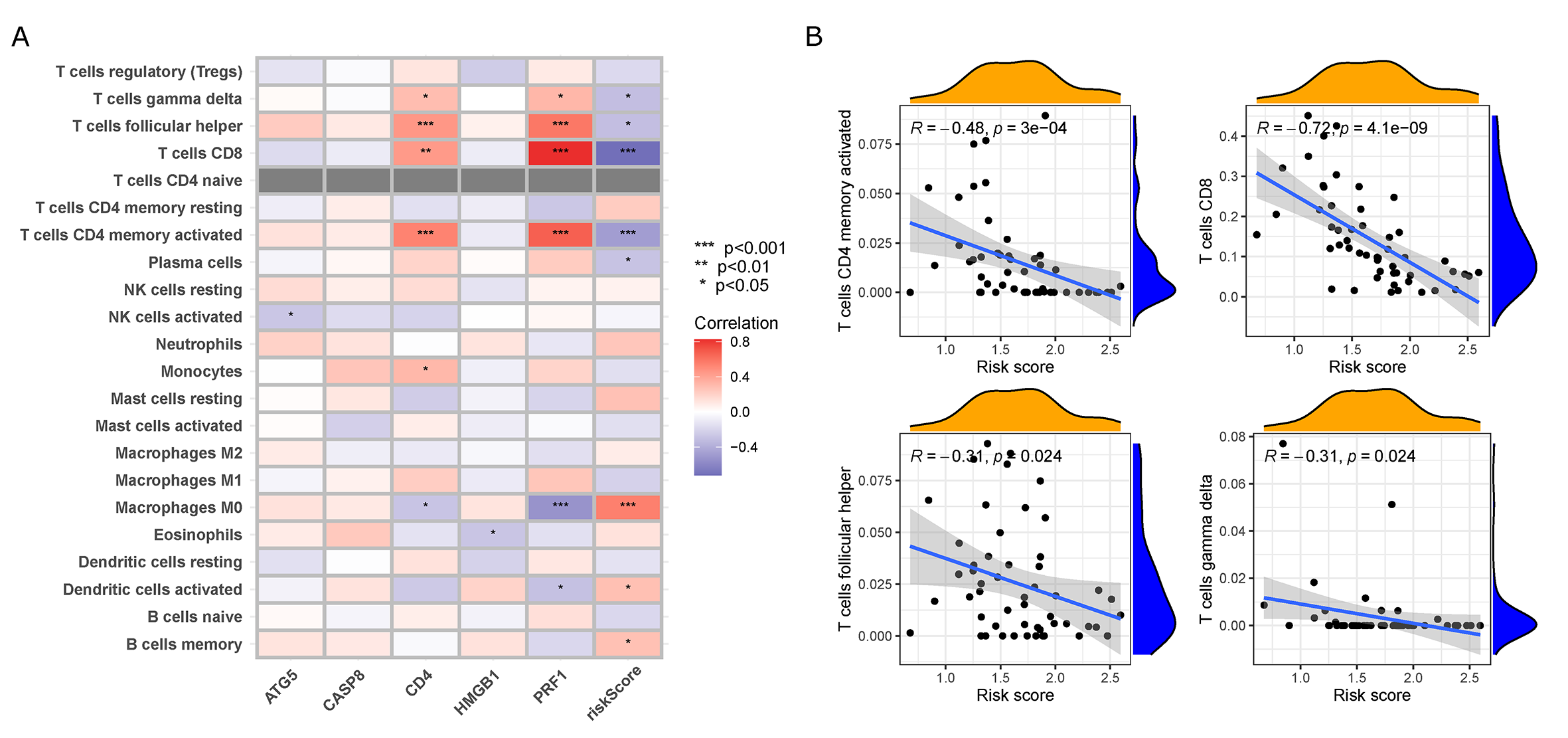

Supplement: Supplementary file 1 — Additional file 1: Figure S1. A. Frequenciesof CNV gain, loss, and non-CNV among ICDs in ICD-low clusters. B. Frequenciesof CNV gain, loss, and non-CNV among ICDs in ICD-high clusters. Figure S2. A. mRNAlevels of BAX in THLE-2 and HCC cells. B. mRNA levels of BAX in HepG2 and Huh7HCC cells after BAX was knocked down. C-D. A colony formation assay was used toexplore the function of BAX in HCC cells. Their representative images are shownin C. E-F. Knockdown of BAX inhibits HCC cell migration. Wound healing assayswere used to assess the migration of HepG2 and Huh7 cells after the BAXknockdown. Representative images are shown in E (* P<0.05, ** P<0.01, ***P<0.001).All experiments were repeated at least three times. Figure S3. A. Differencesin the expression of RNA modification genes between ICD-low and ICD-highclusters. B. Differences in the expression of chemokine genes between ICD-lowand ICD-high clusters. C. Differences in the expression of receptor genesbetween ICD-low and ICD-high clusters. D. Differences in the expression of HLAgenes between ICD-low and ICD-high clusters. Figure S4. A. Frequencies of CNVgain, loss, and non-CNV among ICDs in Risk-high clusters. B. Frequencies of CNVgain, loss, and non-CNV among ICDs in Risk-low clusters. Figure S5. A.Prognostic differences according to high or low TMB scores in TCGA. B.Comparison of ICDRM and TMB in predicting prognosis. C. Heatmap of immuneinfiltration differences between ICDRM subpopulations and ICD clusters in TCGA.Figure S6. A. Differences in the expression of RNA modification genes between ICDRMRsk-low and Risk-high subpopulations. B. Differences in the expression ofchemokine genes between ICDRM Risk-low and Risk-high subpopulations. C.Differences in the expression of receptor genes between ICDRM Risk-low and Risk-highsubpopulations. D. Differences in the expression of HLA genes between ICDRM Risk-lowand Risk-high subpopulations. Figure S7. Analysis of drug sensitivity between ICDRMRisk-low and Risk-high subpo [file 12885_2023_10992_MOESM1_ESM.zip › Supplementary Figure/Supplementary FIG 8.tiff]

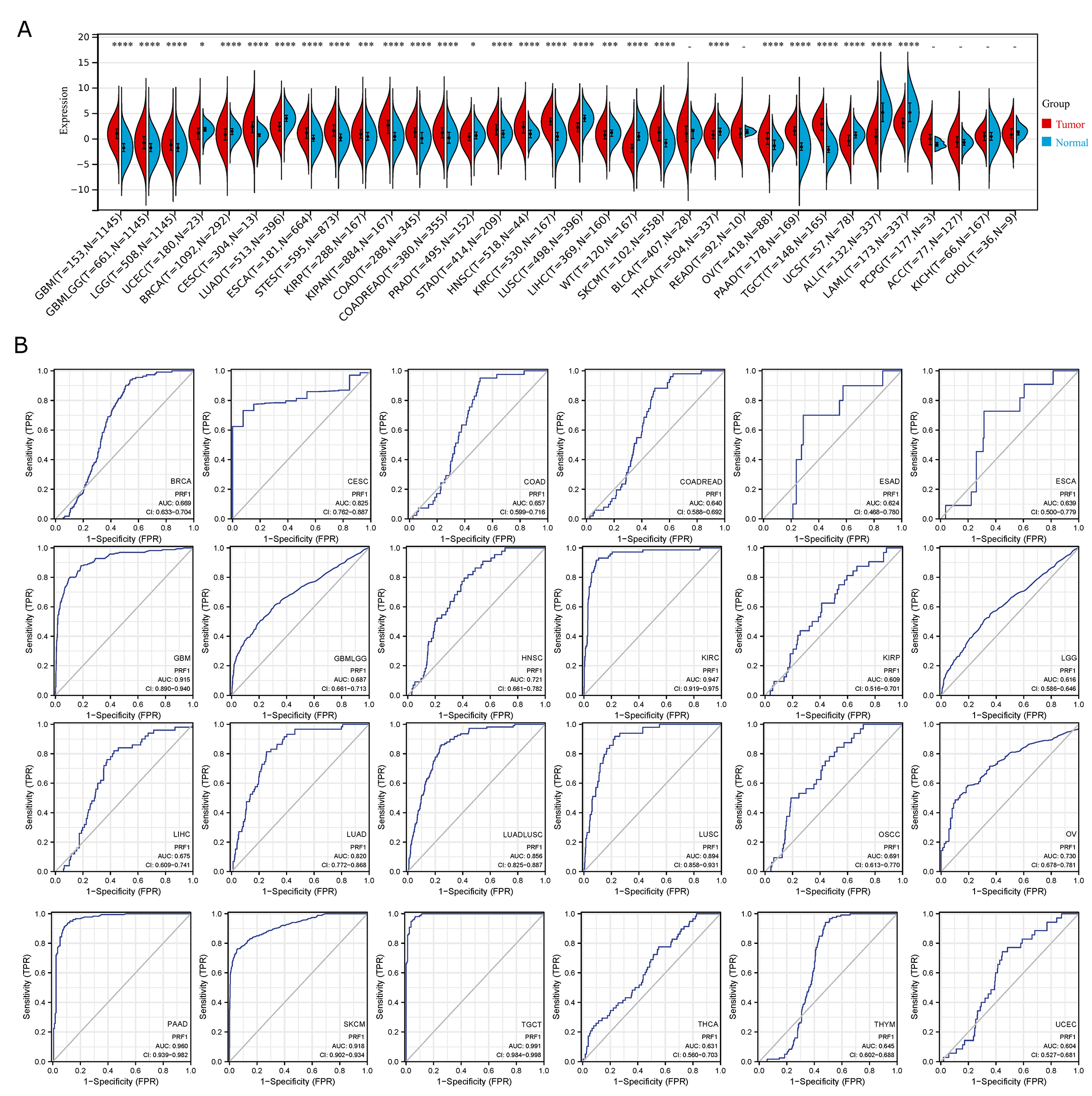

Supplement: Supplementary file 1 — Additional file 1: Figure S1. A. Frequenciesof CNV gain, loss, and non-CNV among ICDs in ICD-low clusters. B. Frequenciesof CNV gain, loss, and non-CNV among ICDs in ICD-high clusters. Figure S2. A. mRNAlevels of BAX in THLE-2 and HCC cells. B. mRNA levels of BAX in HepG2 and Huh7HCC cells after BAX was knocked down. C-D. A colony formation assay was used toexplore the function of BAX in HCC cells. Their representative images are shownin C. E-F. Knockdown of BAX inhibits HCC cell migration. Wound healing assayswere used to assess the migration of HepG2 and Huh7 cells after the BAXknockdown. Representative images are shown in E (* P<0.05, ** P<0.01, ***P<0.001).All experiments were repeated at least three times. Figure S3. A. Differencesin the expression of RNA modification genes between ICD-low and ICD-highclusters. B. Differences in the expression of chemokine genes between ICD-lowand ICD-high clusters. C. Differences in the expression of receptor genesbetween ICD-low and ICD-high clusters. D. Differences in the expression of HLAgenes between ICD-low and ICD-high clusters. Figure S4. A. Frequencies of CNVgain, loss, and non-CNV among ICDs in Risk-high clusters. B. Frequencies of CNVgain, loss, and non-CNV among ICDs in Risk-low clusters. Figure S5. A.Prognostic differences according to high or low TMB scores in TCGA. B.Comparison of ICDRM and TMB in predicting prognosis. C. Heatmap of immuneinfiltration differences between ICDRM subpopulations and ICD clusters in TCGA.Figure S6. A. Differences in the expression of RNA modification genes between ICDRMRsk-low and Risk-high subpopulations. B. Differences in the expression ofchemokine genes between ICDRM Risk-low and Risk-high subpopulations. C.Differences in the expression of receptor genes between ICDRM Risk-low and Risk-highsubpopulations. D. Differences in the expression of HLA genes between ICDRM Risk-lowand Risk-high subpopulations. Figure S7. Analysis of drug sensitivity between ICDRMRisk-low and Risk-high subpo [file 12885_2023_10992_MOESM1_ESM.zip › Supplementary Figure/Supplementary FIG 9.tiff]
